# Dynamic Stereochemistry of a Biphenyl‐Bisprolineamide Model Catalyst and its Imidazolidinone Intermediates

**DOI:** 10.1002/chem.202201317

**Published:** 2022-06-23

**Authors:** Tino P. Golub, Malte Feßner, Elric Engelage, Christian Merten

**Affiliations:** ^1^ Ruhr Universität Bochum Fakultät für Chemie und Biochemie Organische Chemie II Universitätsstraße 150 44801 Bochum Germany

**Keywords:** axial chirality, asymmetric catalysis, computational chemistry, chiroptical spectroscopy, vibrational spectroscopy

## Abstract

In this study, we characterize the dynamic stereochemistry of a biphenyl‐2,2’‐bis(proline amide) catalyst in chloroform and DMSO as representative weakly and strongly hydrogen bonding solvents. Using vibrational circular dichroism (VCD) spectroscopy and density functional theory (DFT) based spectra calculations, we show that the preferred axial stereochemistry of the catalyst is determined by solute‐solvent interactions. Explicitly considering solvation with DMSO molecules is found to be essential to correctly predict the conformational preferences of the catalyst. Furthermore, we investigate the stereochemistry of the corresponding enamines and imidazolidinones that are formed upon reaction with isovaleraldehyde. The enamines are found to rapidly convert to *endo*‐imidazolidinones and the thermodynamically favored *exo*‐imidazolidinones are formed only slowly. The present study demonstrates that the stereochemistry of these imidazolidinones can be deduced directly from the VCD spectra analysis without any further detailed analysis of NMR spectra. Hence, we herein exemplify the use of VCD spectroscopy for an in situ characterization of intermediates relevant in asymmetric catalysts.

## Graphical abstract

**The solvent‐dependent** dynamic stereochemistry of a biphenyl‐2,2’‐bisprolineamide is characterized by VCD spectroscopy. Furthermore, we demonstrate that in situ VCD spectroscopy enables the determination of the imidazolidinones without extensive NMR analysis. In combination with NMR reaction monitoring, even the absolute configuration of a minor imidazolidinone species can be determined.

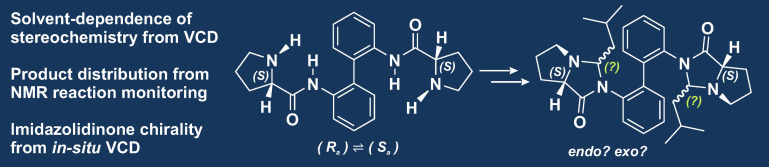

## Introduction

Vibrational circular dichroism (VCD) spectroscopy is the chiroptical version of infrared spectroscopy,^[1]^ which has become an established method for the assignment of absolute configurations.^[2]^ Furthermore, VCD spectra have also been found to be very sensitive to solvent^[3]^ or generally interaction‐induced^[4]^ conformational changes. In fact, as the analysis of an experimental VCD spectrum is based on the comparison with a computed spectrum,^[2b]^ detailed knowledge on the conformational preferences is required^[5]^ and missing an important conformation, even one that only becomes highly populated due to solute‐solvent interactions,^[6]^ may lead to wrong conclusions from the spectra analysis. In strongly hydrogen bonding solvents, for instance, solvation must thus often be accounted for explicitly to obtain a good match between experimental and computed spectra.^[7]^ We recently began to exploit the conformational sensitivity of VCD spectroscopy to gain new insights into the structural preferences of asymmetric catalysts.^[8]^ Besides examples from ion‐pairing^[9]^ and hydrogen bonding catalysis,^[10]^ we also investigated Jørgensen‐Hayashi‐type prolinol ethers^[11]^ and MacMillian's imidazolidinone catalysts^[12]^ as examples for covalent organocatalysts. The corresponding enamine and iminium ion species were generated in situ by reacting the catalysts with suitable aldehydes directly in the IR cuvette. VCD spectroscopy thus enabled a characterization of E/Z‐preferences and other key structural aspects.^[13]^

**Scheme 1:**
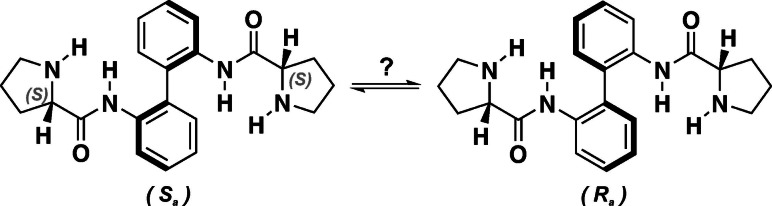
Structure of the biphenyl‐2,2’‐bis(prolineamide) **1**. Throughout the study, it is referred to the (*S*,*S*)‐enantiomer of **1**, while all discussed chiroptical data have also been obtained for (*R*,*R*)‐**1**.

While the chemical nature and the kinetics of the formation of enamines and iminium ions can be characterized by standard ^1^H NMR techniques, elucidation of stereochemical aspects typically requires more elaborate NMR methods.^[14]^ This becomes particularly important when additional dynamic axial chirality has to be considered.^[15]^ In fact, among others,^[16]^ axially chiral biphenyl‐ and binaphthyl‐bisprolineamide are frequently discussed as catalysts for a variety of asymmetric transformations.^[17]^ The simplest representative, the biphenyl‐2,2’‐bis(prolineamide) **1**, is often used for comparison with intrinsically axially chiral systems (Scheme 1). Compound **1** and its 6,6’‐dimethoxy derivative have been used as catalysts for the enantioselective synthesis of α‐hydroxy phosphonates, for instance.^[17a]^ The low enantiomeric excess (e.e.) of only 50 % obtained with **1** contrasted with about 70 % e.e. achieved with axially chiral derivatives under identical conditions. Likewise, for Michael additions of ketones and nitro‐olefines, a maximum e.e. of 30 % was achieved with **1**.^[17c]^ Aldol condensations of cyclohexanone and other ketones with different aldehydes in presence of water were possible in good yields but lower diastereoselectivity and especially lower enantioselectivity compared to axially chiral derivatives.^[17b]^ The comparison of **1** with 6,6’‐dimethoxy derivatives also revealed that only the stereochemistry of proline controls the stereochemical outcome of reactions catalyzed with these biphenyl‐bisprolineamide^[17a]^ while the configuration of the biphenyl axis appeared to play a negligible role. Hence, although the environment around the prolineamide changes rapidly due to the dynamic stereochemistry of the biphenyl unit, the generally smaller biphenyl angle is more likely to cause the inferior performance of **1** compared to axially chiral catalysts.

Herein we demonstrate the use of VCD spectroscopy as reliable method for the direct characterization of the dynamic stereochemistry of **1** and its intermediates in a reaction mixture. We first elucidate the role of solute‐solvent interactions in determining the preferences between (*R_a_*)‐ and (*S_a_*)‐chiral structures and thereby establish a reliable theoretical model for **1**. Afterwards, we investigate the intermediates obtained when **1** reacts with one or two equivalents of isovaleraldehyde. In this context, we show that the enamines of **1** do not have a long lifetime and basically immediately react towards the corresponding imidazolidinones. Finally, we demonstrate that VCD spectroscopy can help unraveling the stereochemistry of the various species present in the reaction mixtures.

## Experimental Section

**Materials**: The catalyst **1** was prepared according to literature procedures.^[17b]^ Isovaleraldehyde was freshly distilled before use, while CDCl_3_ and DMSO‐d_6_ were used without further purification.

**IR and VCD spectroscopy**: The IR and VCD spectra were recorded on a Bruker Vertex FTIR spectrometer equipped with a PMA 50 module for VCD measurements. Samples were held in a transmission cell with BaF_2_ windows and 100 μm path length. Concentration of **1** in all experiments was 0.09 M Spectra were recorded at room temperature with 4 cm^−1^ spectral resolution by accumulating 32 scans for the IR and ∼32000 scans (4 h accumulation time) for VCD. Baseline correction of the VCD spectra was done by subtraction of the spectra of the solvent recorded under identical conditions.

**NMR spectroscopy**: ^1^H NMR reaction monitoring was carried out on a 300 MHz Bruker Avance III NMR spectrometer. Concentrations of **1** was 0.066 M with equimolar CH_2_Br_2_ as internal standard. Reaction monitoring was carried out at room temperature. Spectra analysis and quantification was carried out using MestReNova 14.

**Computational details**: Details on the conformational analysis can be found in the Supporting Information. All geometry optimizations and frequency calculations were carried out at B3LYP/6‐31G+(2d,p) level of theory using the Gaussian 09 Rev. E software package.^[18]^ Solvent effects were taken into account implicitly by using the integral equation formalism of the polarizable continuum model (IEFPCM)^[19]^ of chloroform or DMSO. DMSO‐d_6_ was explicitly considered in all calculations, except for those on **1** for which it is not explicitly stated. Relative energy differences and Boltzmann populations generally refer to the zero‐point corrected energies of the conformers (Δ*E*_ZPC_), if not otherwise stated. Note that we regularly find Δ*E*_ZPC_‐derived populations to better explain the experimental signatures than Gibbs Free energies (for a detailed discussion see Refs. [4a, 6b, 7b, 20], for instance). Vibrational line broadening was simulated by assigning a Lorentzian band shape with half‐width at half‐height of 6 cm^−1^ to the calculated dipole and rotational strength. The calculated frequencies were scaled by 0.98 to account for anharmonic effects not captured by the harmonic approximation employed in the frequency calculations. Figures were prepared using CYLview.^[21]^

**Crystal structure analysis**: The single crystals of endo‐**6** and exo‐**6** were analysed on a Rigaku Synergy dual source device, with Cu micro focus sealed tube (Cu *K*_α_) using mirror monochromators and a HyPix‐6000HE: Hybrid photon counting X‐ray detector. The crystals were mounted in Hampton CryoLoops using GE/Bayer silicone grease. Data was recorded and reduced using the CrysalisPro^[22]^ software. The structure was solved using WinGX^[23]^ in combination with ShelXT^[24]^ and refined with shelXle^[25]^ and ShelXL. Deposition Number(s) 2166106 (for endo‐**6**) and 2168830 (for exo‐**6**) contain(s) the supplementary crystallographic data for this paper. These data are provided free of charge by the joint Cambridge Crystallographic Data Centre and Fachinformationszentrum Karlsruhe Access Structures service.

## Results and Discussion

### Dynamic stereochemistry of 1

At first, we investigated the dynamic stereochemistry of **1** and elucidated the conformational preferences in weakly polar CDCl_3_ and strongly hydrogen bonding DMSO‐d_6_. While the latter solvent was used throughout the study due to its proven capability to stabilize intermediates of enamine catalysis,[^14f^, ^14g^] CDCl_3_ served as benchmark solvent that does not show strong intermolecular interactions. From the ^1^H NMR spectrum of **1**, we noticed two sets of peaks for several hydrogens, which suggested the simultaneous presence of (*R_a_*)‐ and (*S_a_*)‐conformers (cf. Figures S6 and S7). Among others, the 3,3’ protons of the phenyl rings gave two clearly resolved doublets centered at 8.42 and 8.24 ppm in CDCl_3_ and 8.43 and 8.37 ppm in DMSO‐d_6_, respectively. Due to the symmetry of **1**, it is reasonable to assume that each doublet corresponds to one of the axial chiral conformers and that the ratio of the integrated peak areas corresponds to their populations. Interestingly, the ratio of the peak areas is about 63/37 in both solvents, but it is the high‐field peak that is more intense in CDCl_3_ and the low‐field doublet that is stronger in DMSO‐d_6_. Whether this change in the spectral signatures is a result of a change in the axial chiral preferences between diastereomeric (*R_a_*)‐ and (*S_a_*)‐**1** or simply due to (de‐)shielding effects in the two solvents cannot be explained based on the ^1^H NMR spectra alone.

**Figure 1:**
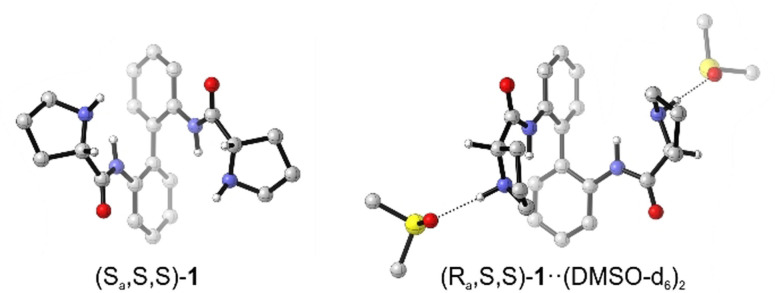
Computed lowest energy structures of isolated (*S*,*S*)‐**1** and the twofold solvated cluster (*S*,*S*)‐**1**⋅⋅(DMSO‐d_6_)_2_. Hydrogen atoms other than the polar N−H and those at the C^α^‐stereocenters are omitted for clarity.

In order to understand these changes in the ^1^H NMR spectra, we carried out a systematic conformational analysis of (*S*,*S*)‐**1**. For this purpose, starting structures for geometry optimizations were generated by manually evaluating various geometric parameters such as the torsional angle defining the biphenyl axis (*R_a_* and *S_a_* isomers), ring puckering of the pyrrolidine rings and the torsional angles N−C(=O)−C^α^−N and C(=O)−C^α^−N−H defining the side group conformations. The amide bonds were held in trans conformation with the N−H pointing towards each other. After performing the geometry optimizations at B3LYP/6‐31G+(2d,p)/IEFPCM(CHCl_3_) level of theory, we obtained a total of 80 conformers with 40 each having (*R_a_*)‐ or (*S_a_*)‐biphenyl configuration (cf. Tables S1–S4 for details on the geometries and relative energies of all computed conformers). Interestingly, according to the computed relative zero‐point corrected electronic energies (Δ*E*_ZPC_), only six conformers are found to be populated within an energy window of 0.5 kcal/mol from the global energetic minimum, while the seventh lowest energy conformer already possesses a much higher energy of 3.25 kcal/mol. Of these six structures, again three each have a (*R_a_*)‐ and three have an (*S_a_*)‐configuration (cf. Figure 1). Besides they only differ in the ring puckering conformation of the pyrrolidine rings.

With a (*R_a_*/*S_a_*)‐ratio of 33 : 67, the computed Δ*E*_ZPC_ pointed towards a clear preference of the (*S_a_*)‐enantiomers, while the relative Gibbs free energies, Δ*G*_298K_, suggested the opposite (*R_a_*/*S_a_*)‐ratio of 71 : 28 based on the same six structures. Re‐optimizing the 80 conformers of **1** in the IEFPCM of DMSO gave a consistent picture for both relative energies (43 : 57 according to Δ*E*_ZPC_, 33 : 62 from Δ*G*_298K_). Hence, given the similarity between the computed ratios and the experimental values from NMR integrations, it may be concluded that (*S_a_*)‐enantiomer dominates in DMSO‐d_6_. Assuming the ΔG_298K_‐energies to represent the experimental conformational distribution would thus suggest a shift in the conformational preferences towards (*R_a_*)‐configuration in CDCl_3_. Note that the subsequent VCD analysis of **1** in the two solvents proved both parts of this conclusion to be incorrect.

**Figure 2:**
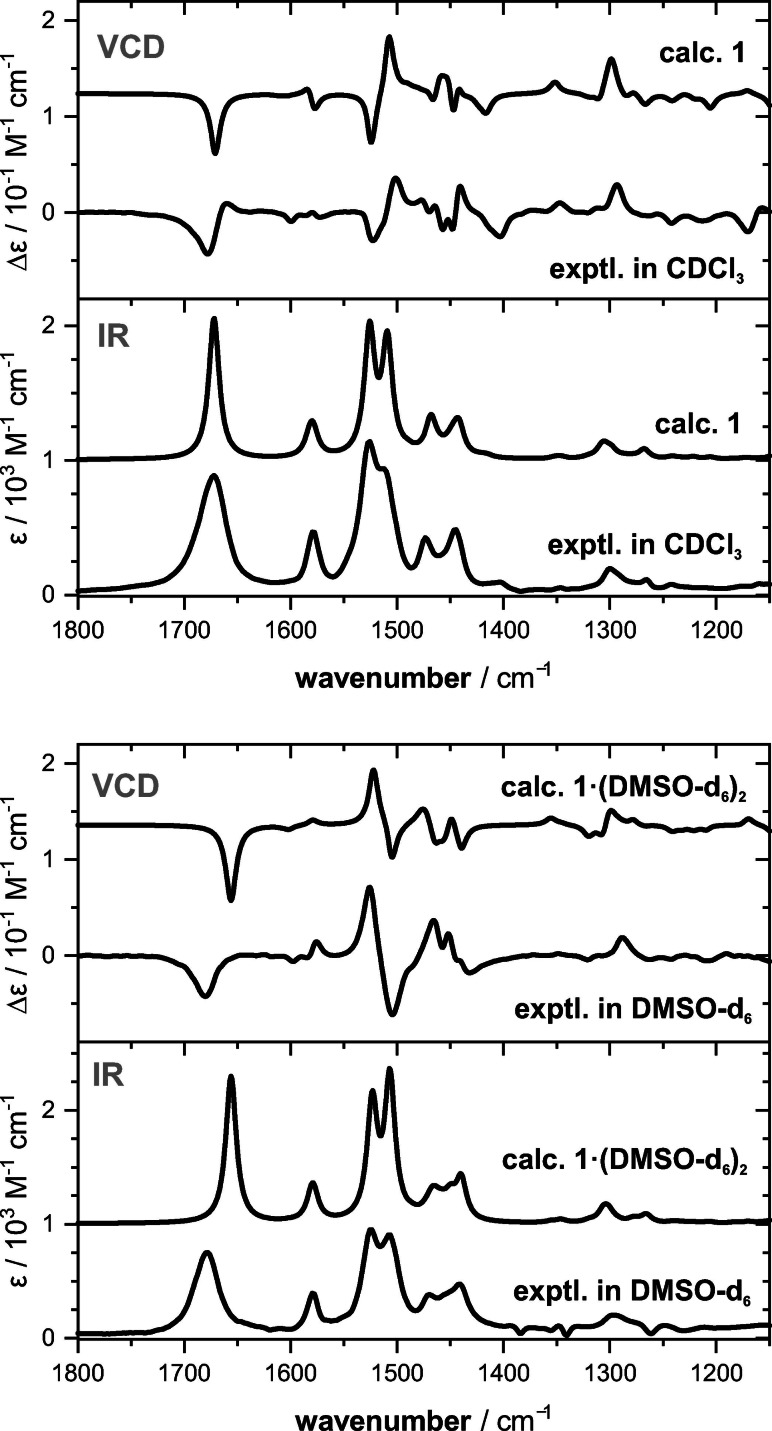
Comparison of the experimental IR and VCD spectra of (*S*,*S*)‐**1** in CDCl_3_ and DMSO‐d_6_ with those computed for an isolated molecule (*S*,*S*)‐**1** and a solute‐solvent cluster (*S*,*S*)‐**1**⋅⋅(DMSO‐d_6_)_2_ at B3LYP/6‐31G+(2d,p)/IEFPCM level of theory. Intensities of the computed IR spectra are scaled by 0.5.

We recorded the IR and VCD spectra of **1** in both CDCl_3_ and DMSO‐d_6_ solvents (Figure 2; a direct comparison showing also the other enantiomer is provided in Figure S1) to complement the characterization of its dynamic stereochemistry. While the fingerprint regions of the two experimental IR spectra did not differ much, the comparison of the VCD spectra revealed significant changes in band intensities and signs: Almost the entire spectral range from 1600–1350 cm^−1^ changed. The most notable difference is the inversion of the couplet at 1525/1500 cm^−^ from (−/+) for **1** in CDCl_3_ to (+/−) in DMSO‐d_6_. These spectral changes again suggest a shift in conformational preferences from one axial chirality to the other.

The final verification of the predominant axially chiral biphenyl structure in CDCl_3_ was obtained by computing the IR and VCD spectra comparing them to the experimental spectra. Interestingly, the spectra corresponding to the Δ*E*_ZPC_‐based conformational distribution were found to match nicely with the experimental spectra of **1** in CDCl_3_ (Figure 2a). Almost all experimental features were reproduced in sign and relative intensity. Based on the calculations, the characteristic feature 1550/1500 cm^−1^ was assigned to the in‐phase and out‐of‐phase amide N−H bending vibrations (also called amide II vibrations), which couple to in‐plane C−H bending modes of the biphenyl unit. In turn, several bands in the range 1500–1400 cm^−1^ arise from various C−H in‐plane bending modes of the biphenyl that couple to amide II vibrations. When computing the VCD signature of the (*R_a_*)‐ and (*S_a_*)‐conformers separately (Figure S3), the long‐range coupling of vibrational modes resulted in a mostly mirror‐image like appearance of the spectra. As a consequence, computing the spectra of (*S*,*S*)‐**1** based on Δ*G*_298K_ with inverted (*R_a_*/*S_a_*)‐ratio gave an almost inverted and thus non‐matching VCD spectrum. Accordingly, comparison of the experimental and computed spectra indeed confirmed the Δ*E*_ZPC_‐based conformational distribution of (*S*,*S*)‐**1** and the preference for (*S_a_*)‐axial chirality.

The almost mirror‐image relation between the computed VCD spectra of the (*R_a_*)‐ and (*S_a_*)‐diastereomers already indicated that the solvent change may indeed causes a conformational shift, but this shift would lead towards a preference of (*R_a_*)‐configuration and thus contradict the computed ratios. At this point it must be noted that explicit hydrogen bonding interactions between **1** and DMSO‐d_6_ have not been considered in the calculations yet. Considering that the N−H bonds of the pyrrolidine rings, which are better hydrogen bond donors than the amide N−H, are pointing towards each other in the (*S_a_*)‐isomers and outwards in the (*R_a_*)‐structures, such conformational shift could be explained by the better accessibility of the hydrogen bond donor groups. In order to substantiate this hypothesis, we added a molecule of DMSO‐d_6_ near each proline N−H bond of the conformers of **1**. This new set of geometries, now comprising two explicit solvent molecules, was re‐optimized at the B3LYP/6‐31G+(2d,p)/IEFPCM level. In line with the qualitative interpretation, the (*R_a_*/*S_a_*)‐ratio of solvated structures **1**⋅⋅(DMSO‐d_6_)_2_ changed towards 57 : 43 (53 : 47) based on Δ*E*_ZPC_ (Δ*G*_298K_). Furthermore, the predicted IR and VCD spectra well reproduced all experimentally observed changes in the spectral signatures (cf. Figure 2b).

From the VCD analysis of **1** in the two solvents, we can indeed conclude that solvation with DMSO‐d_6_ effects the conformational equilibrium. It must be stressed, however, that the correct preferences in CDCl_3_ were only predicted correctly based on Δ*E*_ZPC_ and that explicit solvation was required to correctly predict the preferences in DMSO‐d_6_. Considering the Δ*G*_298K_ energies without explicit solvation would have led to the exact opposite conclusion about the stereochemical preferences.

### Stable intermediates of the reaction with one equivalent of isovaleraldehyde

**Figure 3:**
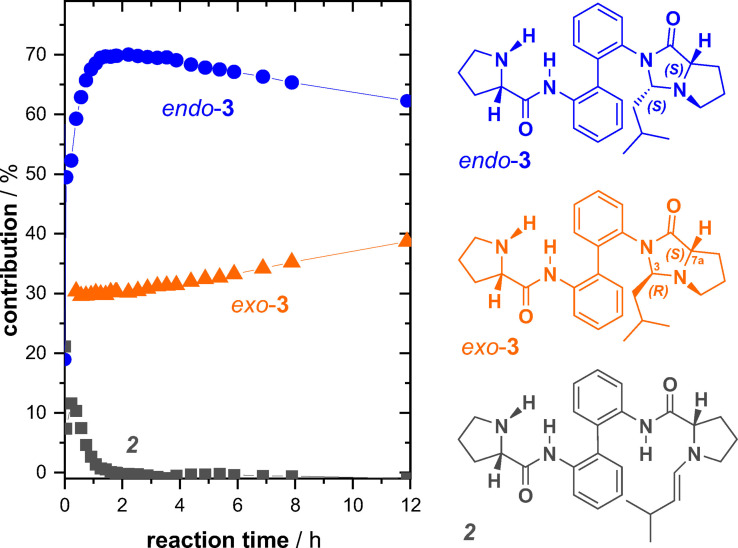
Kinetic profiles obtained from ^1^H NMR reaction monitoring of an equimolar mixture of **1** and isovaleraldehyde.

In the next step of our study, we investigated the intermediates obtained from reacting **1** with an equivalent of isovaleraldehyde. As we expected rapid formation of an enamine, we first conducted ^1^H NMR reaction monitoring of an equimolar mixture of **1** and isovaleraldehyde in DMSO‐d_6_ in order to elucidate the lifetime of the stable intermediates and thereby to determine the ideal time frame during which a VCD spectroscopic measurement should be performed. The formation of the enamine could be confirmed based on the characteristic doublet of the olefin proton (^3^ *J*≈14 Hz, E‐isomer).^[26]^ It should be noted that we observed two doublets, which we attributed to the presence of (*R_a_*)‐**2** and (*S_a_*)‐**2**, respectively (cf. Figure S9). We had to realized, however, that the enamine did not become the major species of the reaction mixture at any time. In fact, its concentration peaked after about 15–20 min of reaction time at about 10 % before it decayed and eventually vanished. We also noted that there are proton signals in the high‐field region (1.2–0.4 ppm), which showed the doublet splitting characteristic for methyl protons of an *i*Pr group. Based on their kinetic profiles and considering that any species in the reaction mixture will likely be present as (*R_a_*)‐ and a (*S_a_*)‐isomers, we identified two additional species besides the enamine (Figure 3): One of the products peaked in concentration after about one hour of reaction time before it slowly decreased, while the other one was slowly but constantly increasing in concentration without reaching the peak of the major product. The ratio between these two products after one hour of reaction time was about 70 : 30.

As enamines of proline‐amides can rapidly react to imidazolidinones,^[27]^ it seemed reasonable to assume that such reaction occurs also in the present case of enamine **2**. Notably, a new stereocenter is formed upon formation of the corresponding imidazolidinone **3** (cf. structures in Figure 3). Furthermore, according to studies on model compounds,^[27]^ the formation of *endo*‐imidazolidinone (3*S*,7a*S*)‐**3** should be kinetically favored and it should be able to convert back to the enamine in catalytic reactions. The *exo*‐isomer (3*R*,7a*S*)‐**3**, however, is supposed to be the thermodynamic product and a dead end for a hypothetical catalytic cycle. According to these studies, the kinetic profiles suggested that the major product should be endo‐**3**, while the minor product is exo‐**3**.

Instead of trying to further disentangle the complex NMR spectra, we again used VCD spectroscopy to elucidate the configurations of the products. To this end, a 1 : 1 mixture of **1** and aldehyde in DMSO‐d_6_ was prepared and the reaction of the sample was monitored using IR spectroscopy. In agreement with the ^1^H NMR reaction monitoring, a steady state was reached after about an hour. At this point, the sample was placed in the VCD instrument and a spectrum was recorded for an accumulation time of about 4 h. While the IR spectrum of the 1 : 1 mixture at the steady state showed only few changes compared to that of **1**, the VCD spectrum revealed several notable differences. A direct comparison of the experimental spectra can be found in Figure S2, but we highlight the new negative VCD feature in the carbonyl region and a significant change in the VCD pattern in the amide II range 1550–1400 cm^−1^.

**Figure 4:**
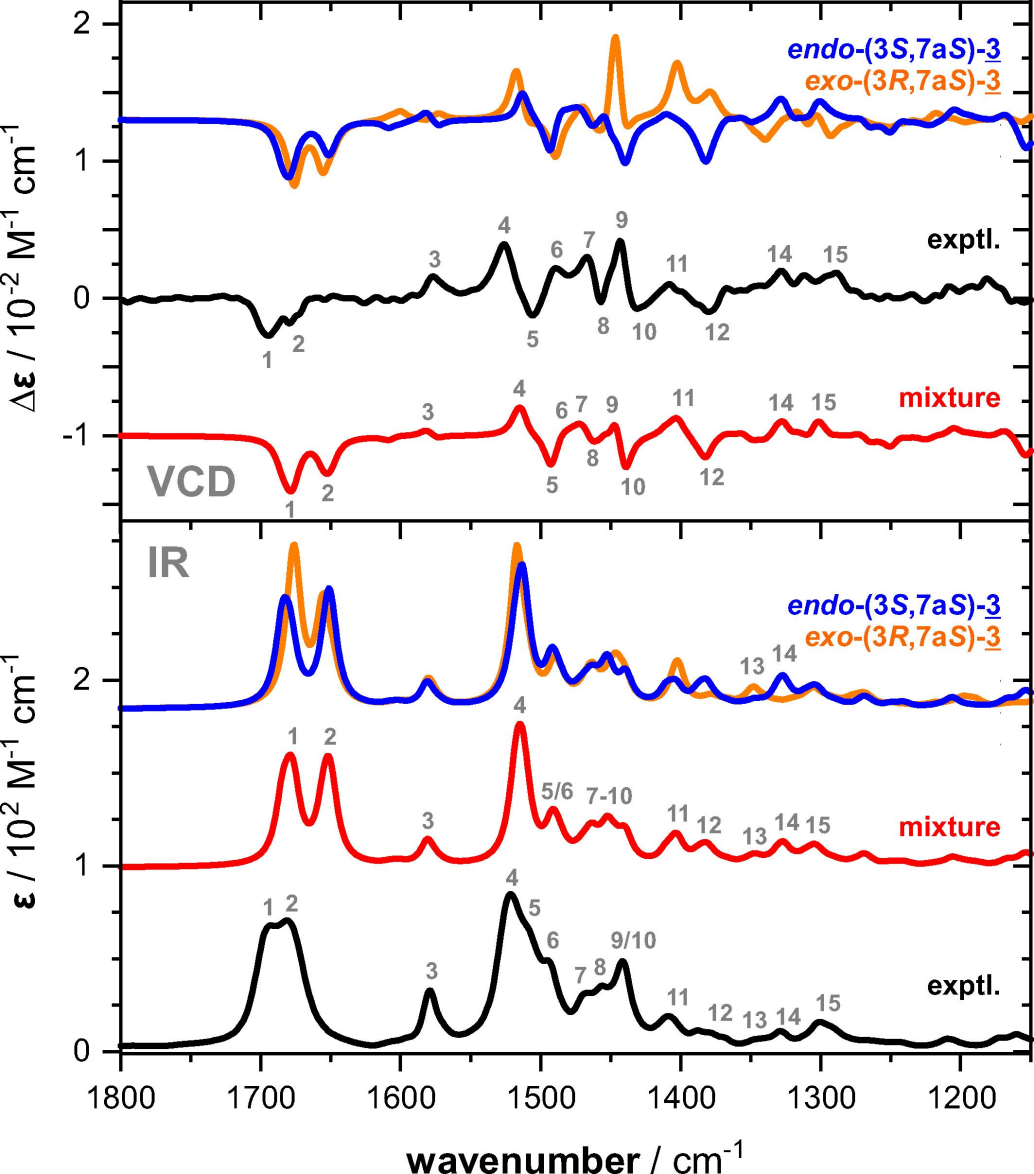
Comparison of the experimental spectra recorded for the 1 : 1 mixture of **1** and isovaleraldehyde in DMSO‐d_6_ (steady state) with the calculated spectra of *endo*‐(3*S*,7a*S*)‐ and *exo*‐(3*R*,7a*S*)‐**3**. The mixture spectrum corresponds to a 70 : 30 mixture of the two computed spectra (see text for details).

For the calculation of the IR and VCD spectra of *endo*‐(3*S*,7a*S*)‐ and *exo*‐(3*R*,7a*S*)‐**3**, explicit solvation with DMSO‐d_6_ was considered for the unreacted proline amide side (cf. Supporting Information for details on the analysis). As for **1**, we found only few conformers of each isomer of **3** to be notably populated. Both diastereomers of **3** favor the (*R_a_*)‐conformation, with (*R_a_*/*S_a_*)‐ratios of 62/38 for *endo*‐ and 77/23 for *exo*‐**3**. The corresponding Boltzmann‐averaged IR and VCD spectra are shown in Figure 4. The computed IR spectra of the diastereomers were very similar with minor and analytically not relevant differences. In contrast, only some bands in the VCD spectra of *endo*‐ and *exo*‐**3** were predicted with the same sign and relative intensities, namely the carbonyl bands and the (+/−) signature in the range 1550–1500 cm^−1^ (band 4/5), which we found to be characteristic for the axial chirality. In the region below 1450 cm^−1^, most bands were of opposite sign for the diastereomers and a band‐by‐band comparison with the experimental spectra suggested that the kinetically favored *endo*‐**3** was indeed the major isomer. The predicted spectrum resembled most experimentally observed signatures reasonably well. It is noted, however, that in particular the experimental VCD bands 9 and 11 were much more intense than predicted for *endo*‐**3**, while the computed VCD spectrum of the diastereomer *exo*‐**3** showed strong bands 9 and 11. Based on the kinetic profiles determined by ^1^H NMR spectroscopy, we computed a representative mixture spectrum with 70 % of *endo*‐ and 30 % of *exo*‐**3**. The resulting IR and VCD spectra are shown in Figure 4 and detailed bands assignments are given to further highlight the excellent resemblance of the experimental spectra. Simulating the spectra with opposite ratios leads to a worse match with the experimental spectra, as bands 10 and 12 would almost vanish. Hence, it can be concluded, that the major imidazolidinone **3** is indeed the *endo*‐(3*S*,7a*S*) species.

### Addition of two equivalents of isovaleraldehyde

Reacting **1** with two equivalents of aldehyde results in a potentially quite complex reaction mixture. The reaction proceeds either via the corresponding dienamine (**4**) or via the mono‐imidazolidinone **3** towards an enamine‐imidazolidinone intermediate **5**, which ultimately leads to the di‐imidazolidinone **6**. As either *endo*‐ or *exo*‐imidazolidinones may be formed, not only the number of chemical species is large, but it also has to be considered again that all constituents are likely to occur as both (*R_a_*)‐ and (*S_a_*)‐isomers. As a consequence, the ^1^H NMR spectra obtained during the reaction monitoring were rather crowded. Nonetheless, three nicely separated sets of doublets of the olefin protons allowed us to monitor the fast decay of enamine **2** and the build‐up and decay of the dienamine **4** and enamine‐imidazolidinone **5**. Analysis of the ^1^H NMR signals in the methyl group region lead to the identification of further two species: One species steadily increased in concentration during the reaction time, while the other one jumped to a concentration of about 30 % within the first hour, where it resided before it began to slowly decay after about four hours.

The major species of the reaction mixture revealed itself by crystallization in the NMR tube after about 20 h. In fact, crystals suitable for X‐ray crystallographic analysis could be obtained repeatedly from DMSO‐d_6_ solution. As anticipated from the results obtained for **3**, the kinetically favoured product was *endo*‐**6** ((3*S*,7a*S*,3’*S*,7a’*S*)‐**6**; cf. Supporting Information for details on the crystallographic analysis). With the knowledge on the structure of the main product, we tentatively assigned an *endo*‐configuration to **5**, as this is the precursor to *endo*‐**6**. The nature of the second‐most abundant species initially remained unclear. Based on the kinetic profiles of other ^1^H NMR signals, another enamine species could be excluded. We noted, however, that there were peaks in the range of the amide‐NH protons that followed the same profile as the methyl protons. Assuming that the species still features an amide functionality and given the similarity in concentration and kinetic profile, we thus tentatively assigned the structure *exo*‐**3** to the side‐product.

In light of the results for the equimolar mixtures of **1** and aldehyde discussed above, we envisioned that VCD spectroscopy could again help in elucidating the structures of the intermediates and to eventually confirm the stereochemical assignments. To this end, we recorded the IR and VCD spectra of 1 : 2 mixtures after about four hours of equilibration in the IR cuvette. Furthermore, we computed the conformational preferences and vibrational spectra of all missing species, i. e., dienamine **4**, endo‐**5** and endo/exo‐**6** (cf. Supporting Information for details on the analysis). For simplicity, we focused in the following analysis only on the spectra of the relevant species and show the computed spectra of all components in the Supporting Information (Figure S4). Likewise, a comparison of the experimental spectra of the 1 : 2‐mixtures with those of pure **1** and of the 1 : 1‐mixture is provided in Figure S2.

**Figure 5:**
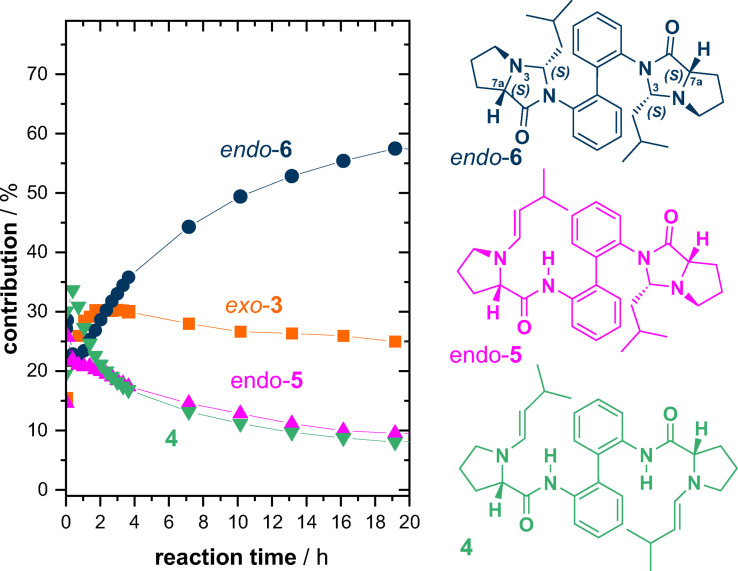
Kinetic profiles obtained from ^1^H NMR reaction monitoring of a 1 : 2‐mixture of **1** and isovaleraldehyde.

**Figure 6:**
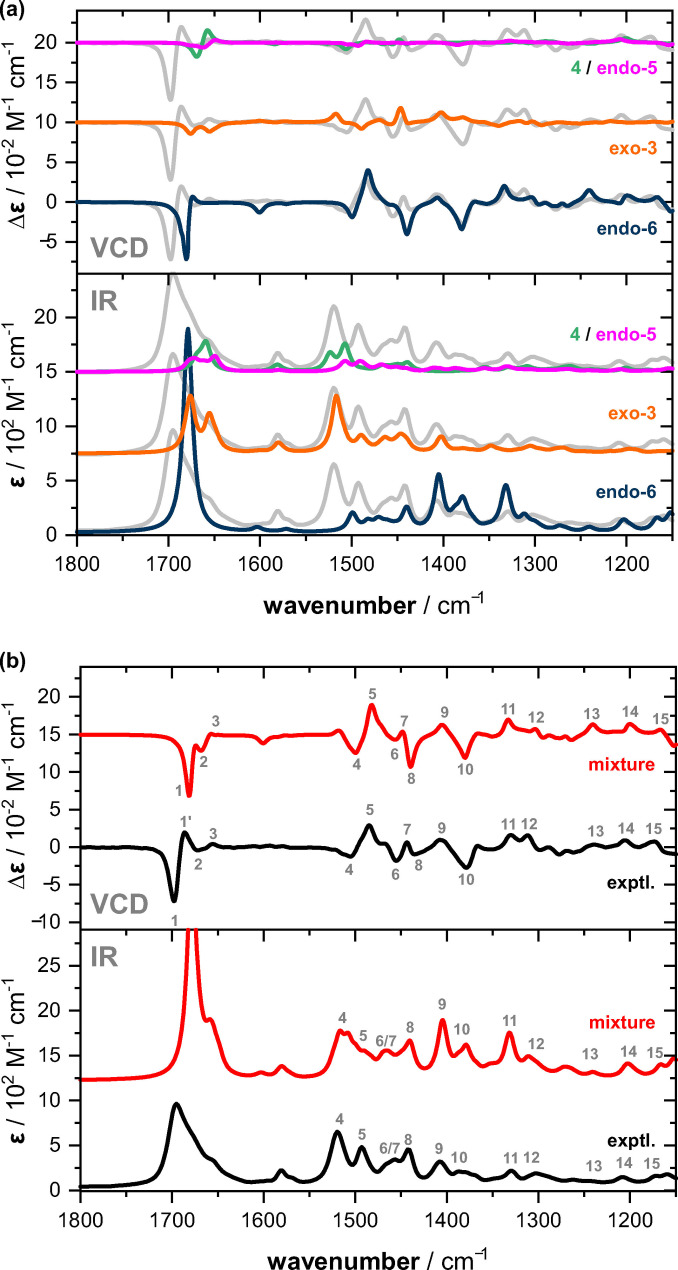
a) Comparison of the experimental spectra recorded for the 1 : 2 mixture of **1** and isovaleraldehyde in DMSO‐d_6_ (steady state) with the intensity‐scaled calculated spectra of *endo*‐**6**, *exo*‐**3** and the enamines **4** and *endo*‐**5**. b) Comparison of the same experimental spectra with the mixture spectra obtained as sum of the scaled single components spectra shown in (a).

Figure 6(a) shows an overlap of the experimental spectra of the 1 : 2‐mixture with the computed IR and VCD spectra of the proposed key components. Note that the computed spectral intensities are already scaled to the respective percentage contributions of the species (cf. kinetic profiles in Figure 5). It can immediately be noticed that the predicted VCD spectrum of endo‐**6** resembled most of the experimental VCD signatures. Solely the (−/+)‐couplet centered at 1660 cm^−1^ was missing and the (−/+)‐signature at 1450 cm^−1^ appeared to be inverted. In the IR spectra, however, discrepancies were obvious and especially the missing intense band at 1520 cm^−1^ stressed that there had to be another species with strong contribution to the spectra. Exactly this missing IR band was nicely reproduced by *exo*‐**3**, which also seemed to be the origin of the (−/+)‐signature in the VCD spectrum at 1450 cm^−1^. Note that the IR spectrum of endo‐**3** was largely identical to that of *exo*‐**3** (cf. Figure 4), but its VCD spectrum did not show the missing 1450 cm^−1^ signature. Finally, the only notable contribution of the enamine species to the VCD spectrum was the (−/+)‐couplet at 1660 cm^−1^. Hence, all tentative assignments made based on the kinetic profiles obtained from ^1^H NMR reaction monitoring can be confirmed through the analysis of the IR and VCD signatures of the reaction mixture.

Based on the weighted single component spectra, we constructed the total IR and VCD spectra of the mixture. As highlighted with band assignments in Figure 6(b), these mixture spectra resembled the experimental ones very well and basically all experimental signatures were reproduced. Solely the positive feature of band 1 is missing, as the C=O stretching modes of endo‐**6** were predicted at slightly too low frequency, so that they overlap with bands of the other species occurring in this region.

Our spectroscopic characterizations have confirmed that the formation of *endo*‐imidazolidinones are kinetically strongly favored. Whether *endo*‐**3** is actually formed in the 1 : 2‐reaction mixture cannot be deduced from the available data. The thermodynamically favored *exo*‐**3**, however, is obviously formed and appeared to be a dead end from which subsequent reactions to *exo*‐**5** or even further do occur very slowly, if at all. A quantitative explanation for the low reactivity of *exo*‐**3** would require further in‐depth computational investigations involving the calculation of transition state energies, which go far beyond the scope of this study. An interesting difference between the conformers of *endo*‐ and *exo*‐**3**, which did not appear particularly worth noting in the discussion of the conformational preferences before, may nonetheless give at least a hint: The biphenyl angle is ±110° for the highly populated (*R_a_*)‐ and (*S_a_*)‐conformers of *endo*‐**3**, while it is in the range −85° to −90° for the highly populated (*R_a_*)‐conformers of *exo*‐**3**, that make up for ∼77 % of the overall Boltzmann weight. As a result of the smaller opening angle, the proline lone pair may be more shielded than in the *endo*‐**3** structures (cf. Figure S5), which in turn could lead to lower reactivity.

Note that heating at 100 °C for 2 h resulted in full conversion to **6**. However, in contrast to reactions at room temperature, for which formation of *endo*‐**6** was observed, the harsher conditions yield *exo*‐**6** ((3*R*,7a*S*,3’*R*,7a’*S*)‐**6**), as confirmed by X‐ray crystallography (cf. Supporting Information for details on the crystallographic analysis).

Finally, we note that there is another diastereomer of **6** with one imidazolidinone moiety possessing *endo*‐ and the other one having an *exo*‐configuration, which is an intermediate in the transition from *endo*‐ to *exo*‐**6**. While we did not find any experimental evidence for its presence, a brief computational survey confirmed that its energy lays in‐between the *endo*‐**6** and *exo*‐**6** diastereomers.

## Conclusion

In the first part of this study, we investigated the dynamic stereochemistry of **1** in different solvents. We found (*S*,*S*)‐**1** to prefer (*S_a_*)‐chiral conformations in weakly polar CDCl_3_ and observed a shift towards the (*R_a_*)‐chiral structures in DMSO‐d_6_. This change was explained by hydrogen bonding interactions of the solvent with the proline‐NHs, which had to be considered explicitly in order to compute the correct conformational preferences. In this regard, it should be stressed once again, that Δ*E*_ZPC_ and Δ*G*_298K_ gave opposite conformational preferences when not considering explicit solvation. The fact that the Δ*G*_298K_‐based preferences were experimentally falsified for CDCl_3_ solution underlines that they should be used carefully when computing entire reaction mechanisms.

In the second part of the study, we demonstrated that VCD spectroscopy can make enormous contribution to structure elucidation of stable reaction intermediates. For the 1 : 1‐mixture of **1** with isovaleraldehyde, VCD spectroscopy gave straightforward access to the stereochemistry of the major reaction product and confirmed the structure as *endo*‐**3**. Likewise, based on kinetic profiles from ^1^H NMR spectroscopic reaction monitoring, VCD enabled assignments of the absolute configurations of the constituents present in the more complex 1 : 2‐mixture of compound **1** and aldehyde.

Concluding the study, it can be stated that the VCD spectroscopic analysis of **1** and its stable reaction intermediates has proven to be greatly complemental to common ^1^H NMR routines. Our study thus encourages future applications of VCD spectroscopy for the characterization of asymmetric catalysts and their preferred conformations and dominant interactions in solution phase.

## Conflict of interest

The authors declare no conflict of interest.

1

## Supporting information

As a service to our authors and readers, this journal provides supporting information supplied by the authors. Such materials are peer reviewed and may be re‐organized for online delivery, but are not copy‐edited or typeset. Technical support issues arising from supporting information (other than missing files) should be addressed to the authors.

Supporting Information

## Data Availability

The data that support the findings of this study are available from the corresponding author upon reasonable request.
