# Supplementary material for: Dynamic Stereochemistry of a Biphenyl‐Bisprolineamide Model Catalyst and its Imidazolidinone Intermediates
Source: Chemistry. 2022 Jun 23;28(45):e202201317. doi: 10.1002/chem.202201317 (PMC9545261; doi:10.1002/chem.202201317)
Supplement: Supplementary file 1 — Supporting Information [file CHEM-28-0-s001.pdf]

Chemistry–A European Journal

Supporting Information

Dynamic Stereochemistry of a Biphenyl-Bisprolineamide Model Catalyst and its Imidazolidinone Intermediates

Tino P. Golub, Malte Feßner, Elric Engelage, and Christian Merten*

Table of Contents

1. Reaction scheme for imidazolidinone formation 2
2. Comparison of experimental IR and VCD spectra 3
3. Computed IR and VCD spectra 4
4. Conformational analysis 5
5. Crystallographic analysis of endo-6 and exo-6 14
6. ^{1}H-NMR spectra of catalyst 1 22
   6.1 Pure catalyst 22
   6.2 300 MHz ^{1}H-NMR reaction monitoring of 1:1 mixture in DMSO-d_6 23
   6.3 300 MHz ^{1}H-NMR reaction monitoring of 1:2 mixture in DMSO-d_6 25
7. Cartesian coordinates 27

1. Reaction scheme for imidazolidinone formation

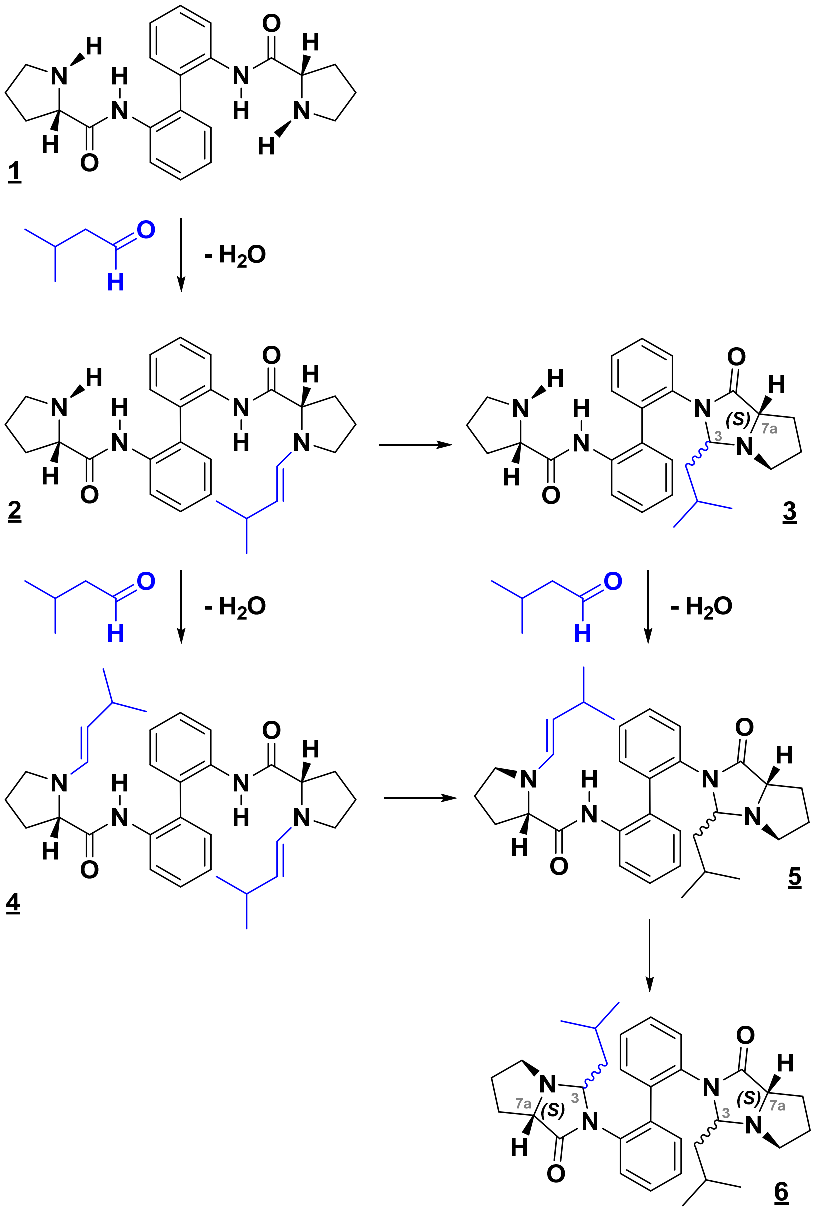

Scheme S1. Possible reaction routes occurring for the 1:2 mixtures of catalyst **1** and isovaleraldehyde.

2. Comparison of experimental IR and VCD spectra

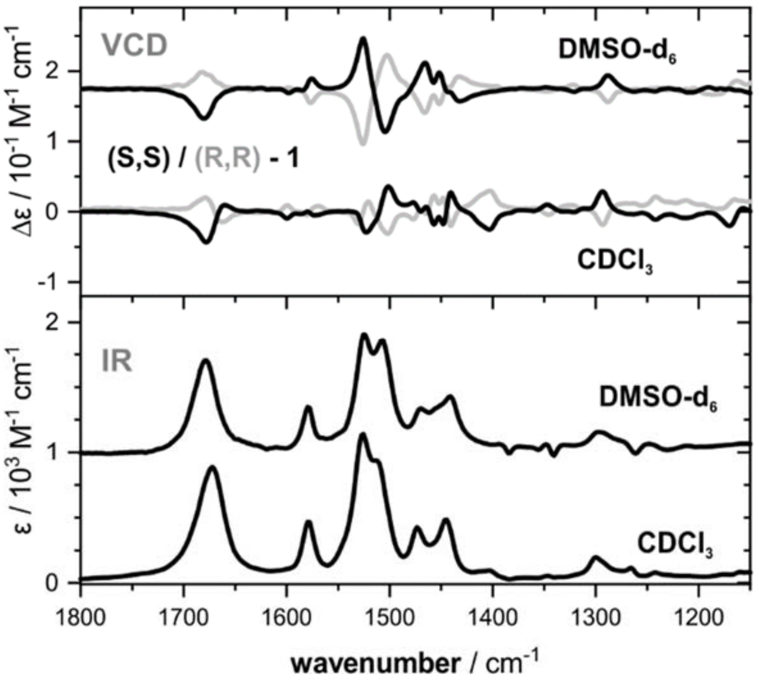

Figure S1. Comparison of the experimental IR and VCD spectra of **1** recorded in $CDCl_3$ and DMSO-d_6.

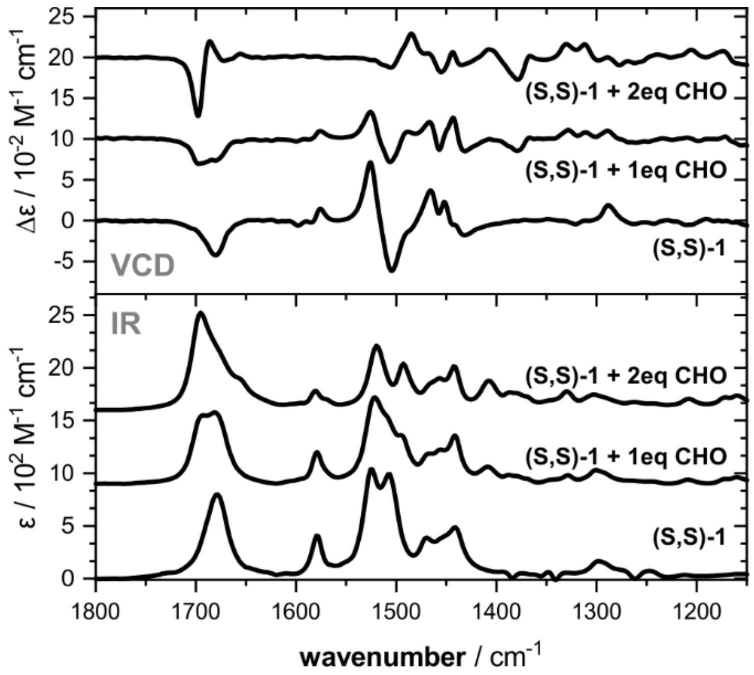

Figure S2. Comparison of the experimental spectra recorded for **1** and its equilibrated 1:1 and 1:2 mixtures with isovaleraldehyde in DMSO-d_6

3. Computed IR and VCD spectra

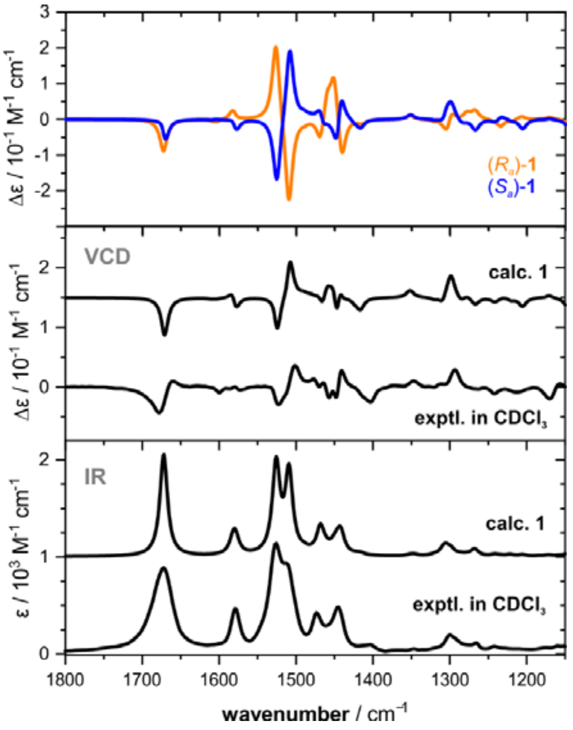

Figure S3. Comparison of the computed VCD signatures of the (R_a)- and (S_a)-conformers of **1** with the experimental spectra. Note that the spectra are essentially identical for calculations using IEFPCM($CHCl_3$) and IEFPCM(DMSO).

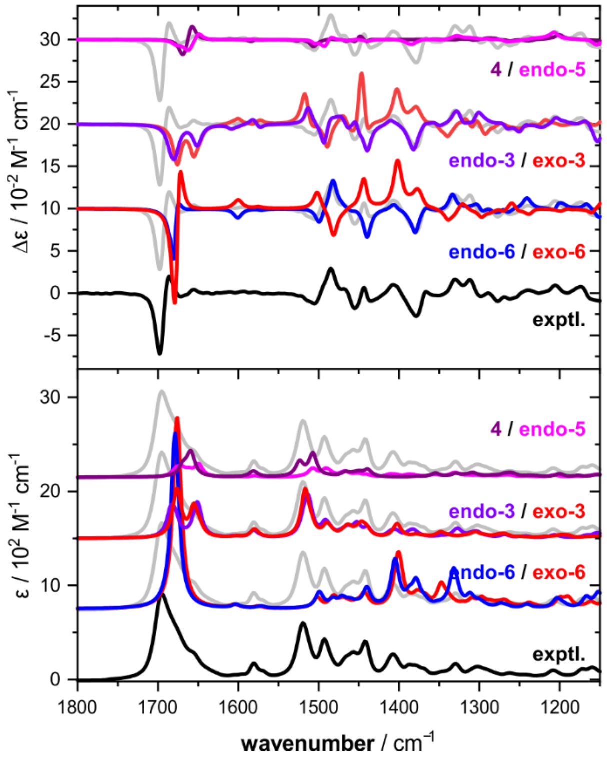

Figure S4. The experimental IR and VCD spectra of the 1:2-mixture compared to the intensity-scaled computed spectra of all possible species present in solution

4. Conformational analysis

The conformational analysis for catalyst **1** was carried out systematically by manually generating conformers through rotation about the bonds indicated in **Scheme S2**. Note that the angle α and the amide bond were kept in trans orientation. After geometry optimizations, we obtained about 80 conformers of which the first six lowest energy conformers already accounted for >99% of the overall Boltzmann distribution. Table S1 shows the geometries and Table S2 the relative energies of the first six conformers of each (S_a)- and (R_a)-axial chirality. All conformers were solvated by placing molecules of DMSO-d_6 near the N-H bonds. Subsequent re-optimization of the structures gave another set of conformers, **1**·(DMSO-d_6)$_2$, which are summarized in Tables S3 (geometries) and S4 (energies and populations).

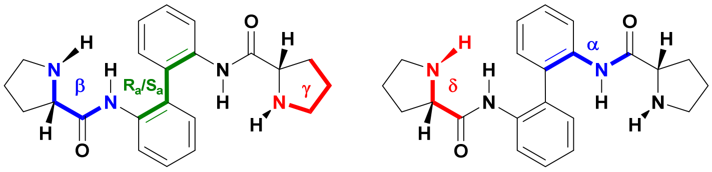

Scheme S2. Torsional angle definitions for catalyst **1**.

Table S1. Torsional angles and definitions of side group conformations for the first six lowest energy conformers of **1** with (S_a) and (R_a) axial chirality. The corresponding energies are given in Table S2.

	α_1	β_1	γ_1	δ_1		α_2	β_2	γ_2	δ_2	
S$_a$-**1**_c1	-165.9	-4.8	38.9	-106.4	= c1^S	-165.9	-4.8	38.9	-106.4	= c1^S
S$_a$-**1**_c2	-166.1	-5.6	-34.9	-111.5	= c2^S	-167.0	-4.5	38.9	-106.7	= c1^S
S$_a$-**1**_c3	-166.2	-4.4	-35.3	-112.7	= c2^S	-165.7	-4.7	-35.2	-112.5	= c2^S
S$_a$-**1**_c4	-171.4	-4.6	-35.4	-113.2	= c2^S	-174.7	157.1	-34.8	8.7	= c3^S
S$_a$-**1**_c5	-171.6	-4.5	38.8	-107.1	= c1^S	-172.4	159.3	-35.8	6.5	= c3^S
S$_a$-**1**_c6	-173.6	-4.9	-35.5	-113.3	= c2^S	-172.5	151.8	36.0	24.3	= c4^S
R$_a$-**1**_c1	179.7	-3.7	-35.7	-114.2	= c2^S	179.7	-3.7	-35.7	-114.2	= c2^R
R$_a$-**1**_c2	179.1	-3.7	38.7	-106.9	= c1^R	179.1	-3.7	38.7	-106.9	= c1^R
R$_a$-**1**_c3	178.7	-3.6	-35.7	-114.3	= c2^R	179.3	-4.3	38.9	-106.4	= c1^R
R$_a$-**1**_c4	177.5	-4.2	38.7	-107.4	= c1^R	175.6	154.0	36.4	22.3	= c4^R
R$_a$-**1**_c5	176.6	-4.7	-35.3	-113.2	= c2^R	174.6	159.8	-35.4	7.3	= c3^R
R$_a$-**1**_c6	176.2	-3.6	-35.9	-115.4	= c2^R	174.4	153.8	36.4	22.6	= c4^R

Table S2. Relative zero-point corrected electronic energies (ΔE_{ZPC}) and Gibbs free energies (ΔG_{298K}) of the first six lowest energy conformers of **1** with (S_a) and (R_a) axial chirality as well as the corresponding Boltzmann populations.

	IEFPCM($CHCl_3$)				IEFPCM(DMSO)			
	ΔE_{ZPC}[a)] [kcal mol^{-1}]	ΔG_{298K}[a)] [kcal mol^{-1}]	pop(ΔE_{ZPC}) [%]	pop(ΔG_{298K}) [%]	ΔE_{ZPC}[b)] [kcal mol^{-1}]	ΔG_{298K}[b)] [kcal mol^{-1}]	pop(ΔE_{ZPC}) [%]	pop(ΔG_{298K}) [%]
S_a-**1**_c1	0.00	0.83	24.5	15.5	0.00	0.22	19.8	18.1
S_a-**1**_c2	0.06	1.02	22.0	11.2	0.04	0.27	18.5	16.7
S_a-**1**_c3	0.12	2.46	19.9	1.0	0.04	0.00	18.4	26.6
S_a-**1**_c4	3.24	2.85	0.1	0.5	3.07	2.21	0.1	0.6
S_a-**1**_c5	3.31	3.95	0.1	0.1	3.21	1.81	0.1	1.2
S_a-**1**_c6	3.32	3.41	0.1	0.2	3.26	1.77	0.1	1.3
R_a-**1**_c1	0.48	0.00	10.7	65.1	0.15	0.28	15.2	16.4
R_a-**1**_c2	0.44	1.98	11.4	2.2	0.17	0.41	14.8	13.2
R_a-**1**_c3	0.48	1.69	10.8	3.6	0.27	1.08	12.5	4.2
R_a-**1**_c4	3.25	3.78	0.1	0.1	3.10	2.67	0.1	0.3
R_a-**1**_c5	3.32	3.14	0.1	0.3	3.10	2.43	0.1	0.4
R_a-**1**_c6	3.33	3.68	0.1	0.1	3.20	2.33	0.1	0.5

[a)] referenced to E_{ZPC}(S_a-**1**_c1) = -1223.159916 hartree and G(R_a-**1**_c1) = -1223.220718 hartree
[b)] referenced to E_{ZPC}(S_a-**1**_c1) = -1223.165143 hartree and G(S_a-**1**_c3) = -1223.225035 hartree

Table S3. Torsional angles and definitions of side group conformations for the first six lowest energy conformers of **1**·(DMSO-d_6)$_2$ with (S_a) and (R_a) axial chirality. The corresponding energies are given in Table S4.

	α_1	β_1	γ_1	δ_1		α_2	β_2	γ_2	δ_2	
S_a-**1**_c1·(DMSO-d_6)$_2$	-173.8	-3.2	-36.5	-118.6	= c2^S	-173.8	-5.6	38.4	-109.1	= c1^S
S_a-**1**_c2·(DMSO-d_6)$_2$	179.9	-4.0	-35.9	-115.9	= c2^S	-177.9	-3.5	-36.2	-116.8	= c2^S
S_a-**1**_c3·(DMSO-d_6)$_2$	178.9	-5.0	38.6	-107.7	= c1^S	-179.8	-6.2	39.1	-104.6	= c1^S
S_a-**1**_c4·(DMSO-d_6)$_2$	-176.8	-3.9	-36.4	-117.8	= c2^S	-175.8	30.5	16.1	55.4	= c5^S
S_a-**1**_c5·(DMSO-d_6)$_2$	-173.2	-4.7	38.2	-109.8	= c1^S	-173.7	30.1	16.4	55.3	= c5^S
S_a-**1**_c6·(DMSO-d_6)$_2$	-175.1	-4.0	37.8	-111.5	= c1^S	-177.4	113.8	-36.8	-121.9	= c6^S
R_a-**1**_c1·(DMSO-d_6)$_2$	178.6	-3.7	-36.3	-117.3	= c2^R	179.2	-3.3	-36.5	-118.0	= c2^R
R_a-**1**_c2·(DMSO-d_6)$_2$	177.7	-4.5	-35.7	-114.5	= c2^R	178.0	-4.9	38.4	-108.7	= c1^R
R_a-**1**_c3·(DMSO-d_6)$_2$	177.5	-4.9	38.5	-108.1	= c1^R	177.1	-5.5	38.7	-107.3	= c1^R
R_a-**1**_c4·(DMSO-d_6)$_2$	178.9	-3.8	-36.4	-117.6	= c2^R	-178.1	120.4	-36.7	-121.5	= c6^S
R_a-**1**_c5·(DMSO-d_6)$_2$	174.5	-4.5	38.3	-109.0	= c1^R	175.1	148.4	27.3	39.7	= c7^R
R_a-**1**_c6·(DMSO-d_6)$_2$	173.3	-4.4	-35.7	-114.6	= c2^R	172.8	148.0	28.1	39.1	= c7^R

Table S4. Relative zero-point corrected electronic energies (ΔE_{ZPC}) and Gibbs free energies (ΔG_{298K}) of the first six lowest energy conformers of **1**·(DMSO-d$_6$)$_2$ with (S$_a$) and (R$_a$) axial chirality as well as the corresponding Boltzmann populations.

	ΔE_{ZPC} [kcal mol^{-1}]	ΔG_{298K} [kcal mol^{-1}]	pop(ΔE_{ZPC}) [%]	pop(ΔG_{298K}) [%]
S$_a$-**1**_c1·(DMSO-d$_6$)$_2$	0.16	2.68	17.4	0.5
S$_a$-**1**_c2·(DMSO-d$_6$)$_2$	0.16	1.46	17.4	3.9
S$_a$-**1**_c3·(DMSO-d$_6$)$_2$	0.58	2.94	8.4	0.3
S$_a$-**1**_c4·(DMSO-d$_6$)$_2$	4.05	4.92	0.0	0.0
S$_a$-**1**_c5·(DMSO-d$_6$)$_2$	4.49	4.32	0.0	0.0
S$_a$-**1**_c6·(DMSO-d$_6$)$_2$	4.58	4.90	0.0	0.0
R$_a$-**1**_c1·(DMSO-d$_6$)$_2$	0.00	0.00	22.9	47.9
R$_a$-**1**_c2·(DMSO-d$_6$)$_2$	0.15	0.30	17.6	28.6
R$_a$-**1**_c3·(DMSO-d$_6$)$_2$	0.21	0.56	16.1	18.4
R$_a$-**1**_c4·(DMSO-d$_6$)$_2$	4.40	3.97	0.0	0.1
R$_a$-**1**_c5·(DMSO-d$_6$)$_2$	4.64	5.33	0.0	0.0
R$_a$-**1**_c6·(DMSO-d$_6$)$_2$	4.66	4.78	0.0	0.0

referenced to E_{ZPC} = -2329.518774 hartree and G = - 2329.609873 hartree

Another comprehensive conformational analysis was carried out for the dienamine **4**. We manually generated conformers through systematic rotation about the bonds indicated in **Scheme S3**, while keeping the angle α and the amide bond in trans orientation. After geometry optimizations, we obtained more than 170 conformers. Table S5 shows the geometries and Table S6 the relative energies of the first six conformers of each (S$_a$)- and (R$_a$)-axial chirality.

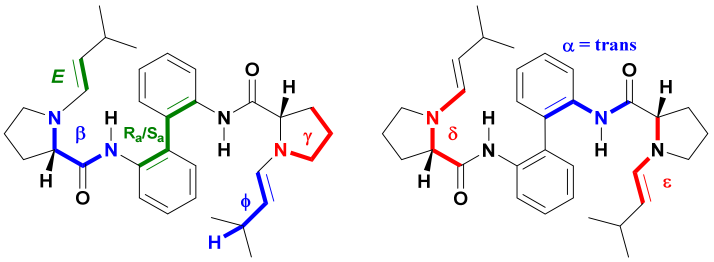

Scheme S3. Torsional angle definitions of the dienamine **4**.

Table S5. Torsional angles and definitions of side group conformations for the first six lowest energy conformers of the dienamine **4** with (S_a) and (R_a) axial chirality. The corresponding energies are given in Table S6.

	β_1	γ_1	δ_1	ε1	ϕ1		β_2	γ_2	δ_2	ε_2	ϕ_2	
**S_a-4_c1**	-10.0	35.0	-94.1	-155.9	-0.2	= $c1^S$	-10.0	35.0	-94.1	-155.9	-0.2	= $c1^S$
**S_a-4_c2**	-14.7	36.6	-91.5	15.8	-0.2	= $c2^S$	-9.8	34.9	-95.7	-155.5	0.0	= $c1^S$
**S_a-4_c3**	-11.0	35.1	-94.0	-155.4	0.2	= $c1^S$	-9.2	34.6	-93.7	-158.1	118.3	= $c4^S$
S_a-**4**_c4	-9.7	34.9	-93.7	-156.1	-0.2	= $c1^S$	-23.6	-25.7	-74.7	-164.5	1.2	= $c5^S$
S_a-**4**_c5	-18.6	-33.4	-87.6	17.1	-0.3	= $c3^S$	-9.2	34.8	-96.3	-155.6	0.0	= $c1^S$
S_a-**4**_c6	-10.7	35.0	-95.0	-155.4	0.2	= $c1^S$	-14.8	36.8	-89.2	15.5	-118.6	= $c6^S$
R_a-**4**_c1	-16.2	37.0	-87.5	14.8	0.8	= $c1^R$	-16.2	37.0	-87.5	14.8	0.8	= $c1^R$
R_a-**4**_c2	-15.6	37.0	-87.8	15.0	0.6	= $c1^R$	-11.0	35.1	-93.0	-156.0	-0.7	= $c2^R$
R_a-**4**_c3	-10.7	35.0	-93.2	-156.2	-0.9	= $c2^R$	-10.7	35.0	-93.2	-156.2	-0.9	= $c2^R$
R_a-**4**_c4	-20.1	-32.1	-83.3	15.7	0.5	= $c3^R$	-10.6	35.0	-93.0	-155.9	-0.6	= $c2^R$
R_a-**4**_c5	-20.2	-32.0	-82.9	15.6	0.1	= $c3^R$	-16.0	37.1	-87.6	14.8	0.4	= $c1^R$
R_a-**4**_c6	-11.1	35.1	-93.2	-156.0	-0.7	= $c2^R$	-24.4	-25.2	-73.4	-164.4	0.1	= $c4^R$

Table S6. Relative zero-point corrected electronic energies (ΔE_{ZPC}) and Gibbs free energies (ΔG_{298K}) of the first six lowest energy conformers of dienamine **4** with (S_a) and (R_a) axial chirality as well as the corresponding Boltzmann populations.

	**ΔE_{ZPC} [kcal mol^{-1}]**	**ΔG_{298K} [kcal mol^{-1}]**	**pop(ΔE_{ZPC}) [%]**	**pop(ΔG_{298K}) [%]**
**S_a-4_c1**	0.00	0.00	14.7	13.4
**S_a-4_c2**	0.53	0.47	6.0	6.0
**S_a-4_c3**	1.08	0.98	2.3	2.5
S_a-**4**_c4	1.11	1.19	2.2	1.7
S_a-**4**_c5	1.20	1.16	1.9	1.8
S_a-**4**_c6	1.26	1.17	1.7	1.8
R_a-**4**_c1	0.09	0.17	12.6	10.0
R_a-**4**_c2	0.13	0.16	11.8	10.2
R_a-**4**_c3	0.22	0.17	10.2	10.0
R_a-**4**_c4	0.64	0.14	4.9	10.5
R_a-**4**_c5	0.74	0.80	4.1	3.4
R_a-**4**_c6	1.12	1.22	2.2	1.6

referenced to E_{ZPC} = -1613.648978 hartree and G = -1613.725971 hartree

Finally, starting from its crystal structure we systematically evaluated the torsional angles shown in Scheme S4 for the conformational analysis of the *endo*-**6**. More than 90 unique conformers were obtained. Table S7 and Table S8 show the geometries and the relative energies of the first six conformers of each (S_a)- and (R_a)-axial chirality, which together account or >99% of the Boltzmann population.

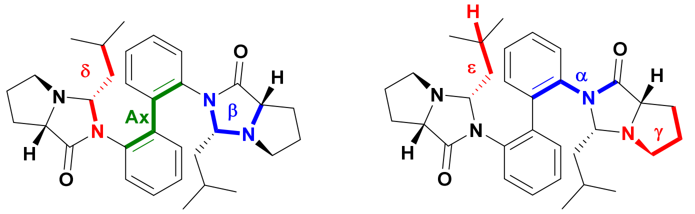

Scheme S4. Torsional angle definitions of **6**.

Table S7. Torsional angles and definitions of side group conformations for the first six lowest energy conformers of *endo*-**6** with (S_a) and (R_a) axial chirality. The corresponding energies are given in Table S8.

	α_1	β_1	γ_1	δ_1	ε_1		α_2	β_2	γ_2	δ_2	ε_2	
R_a-**6**_c1	-101.4	33.0	-40.5	-175.0	-54.5	= c1^R	-101.4	33.0	-40.5	-175.0	-54.5	= c1^R
R_a-**6**_c2	-101.4	33.1	-40.5	-175.0	-54.5	= c1^R	-102.2	33.6	-40.0	-159.2	49.3	= c2^R
R_a-**6**_c3	-103.0	33.2	-40.6	-174.9	-54.6	= c1^R	-103.4	32.7	23.1	-179.3	-58.0	= c3^R
R_a-**6**_c4	-106.1	32.9	-40.4	-82.4	61.0	= c4^R	-102.1	33.0	-40.5	-174.8	-54.8	= c1^R
R_a-**6**_c5	-102.1	33.7	-40.0	-159.2	49.2	= c2^R	-102.1	33.7	-40.0	-159.2	49.2	= c2^R
R_a-**6**_c6	-101.2	32.9	-40.4	-175.0	-54.4	= c1^R	-101.3	34.0	-40.1	-161.7	173.6	=c5^R
S_a-**6**_c1	66.5	30.9	-39.1	-178.8	-55.7	= c1^S	66.5	30.9	-39.1	-178.8	-55.7	= c1^S
S_a-**6**_c2	66.3	31.0	-39.0	-178.9	-55.7	= c1^S	63.4	30.8	-39.1	-158.2	51.6	= c2^S
S_a-**6**_c3	66.3	31.0	-39.1	-178.9	-55.8	= c1^S	65.1	29.6	21.7	177.6	-59.1	= c3^S
S_a-**6**_c4	63.0	30.8	-39.1	-158.2	51.6	= c2^S	63.0	30.8	-39.1	-158.2	51.6	= c2^S
S_a-**6**_c5	65.0	29.6	21.7	177.4	-59.2	= c3^S	63.0	30.8	-39.1	-158.2	51.4	= c2^S
S_a-**6**_c6	66.2	30.9	-39.0	-179.0	-55.7	= c1^S	63.1	31.8	17.7	-161.6	51.0	= c6^S

Table S8. Relative zero-point corrected electronic energies (ΔE_{ZPC}) and Gibbs free energies (ΔG_{298K}) of the first six lowest energy conformers of *endo*-**6** with (S_a) and (R_a) axial chirality as well as the corresponding Boltzmann populations.

	**ΔE_{ZPC} [kcal mol^{-1}]**	**ΔG_{298K} [kcal mol^{-1}]**	**pop(ΔE_{ZPC}) [%]**	**pop(ΔG_{298K}) [%]**
R_a-**6**_c1	2.90	2.62	0.5	0.8
R_a-**6**_c2	4.28	4.03	0.0	0.1
R_a-**6**_c3	4.85	4.50	0.0	0.0
R_a-**6**_c4	5.13	5.27	0.0	0.0
R_a-**6**_c5	5.54	5.25	0.0	0.0
R_a-**6**_c6	5.69	5.65	0.0	0.0
S_a-**6**_c1	0.00	0.00	74.1	69.6
S_a-**6**_c2	0.88	0.81	16.4	17.4
S_a-**6**_c3	1.66	1.45	4.3	5.8
S_a-**6**_c4	1.85	1.92	3.1	2.6
S_a-**6**_c5	2.57	2.28	0.9	1.4
S_a-**6**_c6	2.78	1.98	0.6	2.3

referenced to E_{ZPC} = -1613.642621 hartree and G = -1613.711031 hartree

In order to accelerate the computations of the intermediates *endo*-**3** and *endo*-**5**, starting structures were build using the favored side group conformations found in the symmetric compounds **1**, **4** and *endo*-**6**. In order to build conformers of *endo*-**3**, for instance, we used side group conformer c1 of solvated proline amide taken from Table S3 and side group conformer c1 of the imidazolidinone taken from Table S7.

Table S9. Torsional angles and definitions of side group conformations for the first six lowest energy conformers of endo-**3**·DMSO-d_6 with (S_a) and (R_a) axial chirality. The corresponding energies are given in Table S10.

		**Imidazolidinone** (from endo-**6**, Tab. S7)						**Proline amide** (from 1, Tab. S3)				
	biphenyl	α	β	γ	δ	ε		α	β	γ	δ	
endo-R_a-**3**_c1	-112.5	-132.0	31.7	-40.2	-174.9	-53.7	= $c1^R$	-177.7	-7.6	-36.0	-116.6	= $c2^R$
endo-R_a-**3**_c2	-112.0	-131.4	31.7	-40.2	-174.8	-53.9	= $c1^R$	-178.9	-7.9	38.3	-109.3	= $c1^R$
endo-R_a-**3**_c3	-112.7	-131.3	31.5	-40.0	-156.0	51.9	= $c2^R$	-177.8	-7.1	-36.1	-117.0	= $c2^R$
endo-R_a-**3**_c4	-112.6	-130.9	31.5	-40.0	-155.8	51.7	= $c2^R$	-179.2	-7.1	38.2	-109.6	= $c1^R$
endo-R_a-**3**_c5	-111.7	-132.3	30.8	24.7	-179.5	-57.4	= $c3^R$	-178.1	-6.2	-36.0	-116.0	= $c2^R$
endo-R_a-**3**_c6	-111.1	-132.0	30.8	24.7	-179.6	-57.6	= $c3^R$	-178.2	-8.0	38.3	-109.3	= $c1^R$
endo-S_a-**3**_c1	107.7	92.8	31.5	-39.6	-175.8	-54.6	= $c1^S$	-178.5	-2.3	-36.1	-116.3	= $c2^S$
endo-S_a-**3**_c2	109.6	95.9	31.8	-39.6	-175.9	-54.9	= $c1^S$	-179.4	-3.2	38.2	-108.9	= $c1^S$
endo-S_a-**3**_c3	103.7	87.6	31.6	-39.1	-158.9	50.0	= $c2^S$	-178.2	-0.7	-37.1	-121.7	= $c2^S$
endo-S_a-**3**_c4	108.7	95.3	30.8	-39.4	-85.4	47.5	= $c4^S$	-178.3	-0.6	-36.8	-119.6	= $c2^S$
endo-S_a-**3**_c5	109.5	93.8	32.1	-39.1	-158.9	50.0	= $c2^S$	-178.7	-2.2	37.5	-112.5	= $c1^S$
endo-S_a-**3**_c6	110.0	97.5	31.0	-39.5	-84.8	47.7	= $c4^S$	-179.4	-1.6	37.4	-112.5	= $c1^S$

Table S10. Relative zero-point corrected electronic energies (ΔE_{ZPC}) and Gibbs free energies (ΔG_{298K}) of the first six lowest energy conformers of *endo*-**3**·DMSO-d_6 with (S_a) and (R_a) axial chirality as well as the corresponding Boltzmann populations.

	**ΔE_{ZPC} [kcal mol^{-1}]**	**ΔG_{298K} [kcal mol^{-1}]**	**pop(ΔE_{ZPC}) [%]**	**pop(ΔG_{298K}) [%]**
endo-R_a-**3**_c1	0.00	0.00	27.5	46.4
endo-R_a-**3**_c2	0.16	0.52	20.9	18.9
endo-R_a-**3**_c3	1.22	1.18	3.4	6.1
endo-R_a-**3**_c4	1.43	2.56	2.4	0.6
endo-R_a-**3**_c5	1.53	2.02	2.0	1.5
endo-R_a-**3**_c6	1.91	2.74	1.0	0.4
endo-S_a-**3**_c1	0.24	1.28	18.1	5.2
endo-S_a-**3**_c2	0.39	0.81	14.2	11.6
endo-S_a-**3**_c3	1.66	3.25	1.6	0.2
endo-S_a-**3**_c4	1.69	2.89	1.5	0.3
endo-S_a-**3**_c5	1.73	2.17	1.4	1.1
endo-S_a-**3**_c6	1.84	2.65	1.2	0.5

referenced to E_{ZPC} = -1971.581673 hartree and G = -1971.662015 hartree

Table S11. Torsional angles and definitions of side group conformations for the first six lowest energy conformers of endo-**5** with (S$_a$) and (R$_a$) axial chirality. The corresponding energies are given in Table S12.

		**enamine** (from **4**, Tab S5.)						**imidazolidinone** (from endo-**6**, Tab. S7)					
	biphenyl	β	γ	δ	ε	ϕ		α	β	γ	δ	ε	
endo-S$_a$-**5**_c1	101.8	-14.7	34.4	-90.9	-157.8	-1.4	= c1^S	94.3	31.5	-39.9	-175.6	-54.7	= c1^S
endo-S$_a$-**5**_c2	100.6	-28.3	-25.0	-70.9	-165.1	0.0	= c5^S	93.4	31.5	-39.9	-175.4	-54.4	= c1^S
endo-S$_a$-**5**_c3	101.9	-13.9	34.3	-90.6	-158.5	118.2	= c4^S	94.3	31.5	-39.9	-175.7	-54.8	= c1^S
endo-S$_a$-**5**_c4	101.8	-14.5	34.4	-91.0	-157.2	-121.2	= c7^S	94.1	31.6	-39.8	-175.8	-55.0	= c1^S
endo-S$_a$-**5**_c5	102.0	-19.2	36.4	-86.3	13.6	0.8	= c2^S	93.9	31.5	-39.8	-175.6	-54.9	= c1^S
endo-S$_a$-**5**_c6	101.7	-23.4	-32.1	-81.6	14.7	0.6	= c3^S	93.5	31.5	-39.9	-175.6	-54.8	= c1^S
endo-R$_a$-**5**_c1	-105.5	-10.3	33.7	-95.8	-157.7	1.5	= c1^R	-128.7	31.6	-40.2	-174.9	-54.1	= c1^R
endo-R$_a$-**5**_c2	-104.2	-23.6	-25.8	-75.8	-161.9	6.6	= c1^R	-127.5	31.6	-40.1	-175.0	-54.1	= c1^R
endo-R$_a$-**5**_c3	-104.4	-11.8	32.8	-95.4	-160.5	116.3	= c9^R	-127.3	31.7	-40.1	-174.9	-54.0	= c1^R
endo-R$_a$-**5**_c4	-105.5	-11.0	33.8	-95.5	-157.5	1.4	= c1^R	-127.9	31.4	-40.0	-156.6	51.5	= c2^R
endo-R$_a$-**5**_c5	-105.3	-11.2	33.5	-96.5	-157.9	-121.6	= c11^R	-128.4	31.7	-40.1	-175.1	-54.1	= c1^R
endo-R$_a$-**5**_c6	-104.2	-26.5	36.1	-91.4	14.0	-4.2	= c2^R	-126.9	31.7	-40.2	-174.8	-53.8	= c1^R

Table S12. Relative zero-point corrected electronic energies (ΔE_{ZPC}) and Gibbs free energies (ΔG_{298K}) of the first six lowest energy conformers of *endo*-**5** with (S$_a$) and (R$_a$) axial chirality as well as the corresponding Boltzmann populations.

	**ΔE_{ZPC} [kcal mol^{-1}]**	**ΔG_{298K} [kcal mol^{-1}]**	**pop(ΔE_{ZPC}) [%]**	**pop(ΔG_{298K}) [%]**
endo-S$_a$-**5**_c1	0.24	0.10	15.4	22.6
endo-S$_a$-**5**_c2	1.08	1.10	3.6	4.1
endo-S$_a$-**5**_c3	1.33	1.53	2.4	1.9
endo-S$_a$-**5**_c4	1.44	1.52	2.0	2.0
endo-S$_a$-**5**_c5	0.31	0.00	13.7	26.9
endo-S$_a$-**5**_c6	0.87	0.98	5.2	5.0
endo-R$_a$-**5**_c1	0.00	0.48	23.3	11.9
endo-R$_a$-**5**_c2	1.41	1.47	2.1	2.1
endo-R$_a$-**5**_c3	1.09	1.67	3.6	1.5
endo-R$_a$-**5**_c4	1.15	1.45	3.2	2.2
endo-R$_a$-**5**_c5	1.25	1.79	2.7	1.2
endo-R$_a$-**5**_c6	1.36	1.90	2.2	1.0

referenced to E_{ZPC} = -1613.648239 hartree and G = -1613.720526 hartree

For exo-**3** and exo-**6**, we carried out a systematic analysis starting from the structure of the corresponding endo-isomers and inverting the stereocenters of the most populated conformers.

Table S13. Torsional angles, relative zero-point corrected electronic energies (ΔE_{ZPC}) and Gibbs free energies (ΔG_{298K}) of the first six lowest energy conformers of *exo*-**3** with (S_a) and (R_a) axial chirality as well as the corresponding Boltzmann populations.

	exo-imidazolidinone					proline-amide							
	biphenyl	β	γ	δ	ε	α	β	γ	δ	ΔE_{ZPC}	ΔG_{298K}	pop(ΔE)	pop(ΔG)
exo-R$_a$-**3**_c1	-85.5	23.0	-40.4	175.6	50.0	176.8	-5.2	-34.7	-110.4	0.00	0.37	32.3	20.9
exo-R$_a$-**3**_c2	-90.4	23.0	-40.4	175.9	50.6	-179.0	-4.6	38.9	-104.9	0.35	1.04	17.8	6.6
exo-R$_a$-**3**_c3	-87.3	20.0	30.5	176.4	50.6	178.2	-4.3	-35.2	-111.7	0.67	1.09	10.2	6.0
exo-R$_a$-**3**_c4	-90.0	19.7	30.8	176.5	50.8	-179.9	-5.1	39.0	-103.5	1.07	1.65	5.1	2.3
exo-R$_a$-**3**_c5	-83.8	23.9	-40.2	162.5	-50.3	175.1	-5.2	-34.7	-110.3	1.24	2.03	3.8	1.2
exo-R$_a$-**3**_c6	-88.6	24.0	-40.3	163.4	-49.2	-179.2	-5.1	39.0	-104.2	1.50	2.16	2.4	1.0
exo-S$_a$-**3**_c1	112.7	17.5	-40.3	174.5	49.2	147.8	-4.6	38.7	-107.1	0.68	1.29	10.0	4.3
exo-S$_a$-**3**_c2	112.5	10.1	32.9	174.3	48.6	147.3	-1.6	-36.6	-118.5	0.96	1.00	6.2	7.0
exo-S$_a$-**3**_c3	112.7	10.2	33.0	174.4	49.0	147.3	-4.9	38.9	-106.2	1.11	0.92	4.8	8.1
exo-S$_a$-**3**_c4	113.3	18.3	-40.3	165.8	-45.5	154.2	-2.1	-35.4	-112.9	1.79	2.50	1.5	0.5
exo-S$_a$-**3**_c5	113.3	11.4	32.4	166.4	-44.3	148.7	-4.2	38.5	-107.5	2.50	3.31	0.4	0.1
exo-S$_a$-**3**_c6	112.4	19.3	-40.2	166.5	-175.6	151.9	-2.2	-35.3	-112.3	3.17	4.07	0.1	0.0

referenced to E_{ZPC} = -1971.585084 hartree and G = -1971.666191 hartree

Table S14. Torsional angles, relative zero-point corrected electronic energies (ΔE_{ZPC}) and Gibbs free energies (ΔG_{298K}) of the first six lowest energy conformers of *exo*-**6** with (S_a) and (R_a) axial chirality as well as the corresponding Boltzmann populations.

	β	γ	δ	ε	β	γ	δ	ε	ΔE_{ZPC}	ΔG_{298K}	pop(ΔE)	pop(ΔG)
exo-R$_a$-**6**_c1	21.5	-40.5	176.4	50.6	19.5	29.6	177.1	50.8	0.0	0.0	62.5	60.9
exo-R$_a$-**6**_c2	19.8	29.6	177.1	50.9	19.8	29.6	177.1	50.9	0.9	1.1	12.4	8.8
exo-R$_a$-**6**_c3	22.9	-40.4	162.5	-50.2	19.4	29.8	177.0	50.8	1.2	1.3	8.6	6.4
exo-R$_a$-**6**_c4	21.8	-40.5	176.2	50.6	20.6	28.4	166.4	-45.9	1.3	0.9	7.2	13.8
exo-R$_a$-**6**_c5	22.7	-40.5	85.5	-47.4	20.9	-40.3	176.4	50.4	1.7	2.5	3.6	0.9
exo-R$_a$-**6**_c6	19.5	30.2	177.0	50.8	20.9	28.5	165.7	-46.5	2.1	1.9	1.7	2.5
exo-S$_a$-**6**_c1	21.2	-39.2	163.5	-48.2	19.0	-39.9	176.2	49.8	7.5	7.6	0.0	0.0
exo-S$_a$-**6**_c2	19.3	-39.8	176.1	49.4	1.1	34.6	174.1	-35.1	8.0	8.0	0.0	0.0
exo-S$_a$-**6**_c3	21.1	-39.3	164.0	-48.6	21.1	-39.3	164.0	-48.6	8.9	8.5	0.0	0.0
exo-S$_a$-**6**_c4	18.2	-39.7	169.0	-41.5	-0.3	35.0	174.9	-34.5	9.5	8.9	0.0	0.0
exo-S$_a$-**6**_c5	20.9	-39.2	163.9	-48.3	21.2	-39.3	166.0	-175.4	10.2	10.8	0.0	0.0
exo-S$_a$-**6**_c6	20.4	-39.5	166.1	-174.9	-0.6	35.0	174.2	-35.1	10.8	11.2	0.0	0.0

referenced to E_{ZPC} = -1613.650376 hartree and G = -1613.720979 hartree

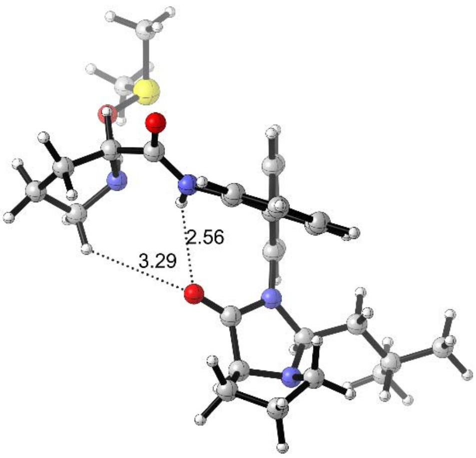

R$_a$-*endo*-**3**_c1

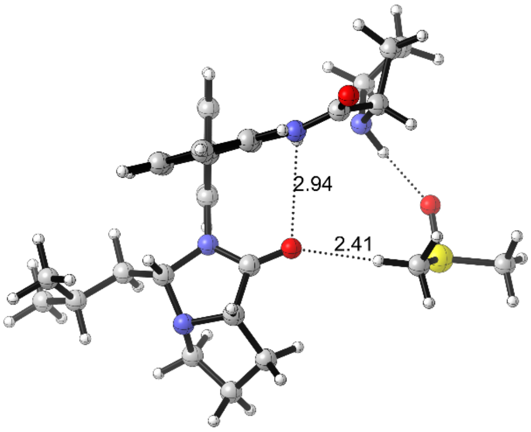

S$_a$-*endo*-**3**_c1

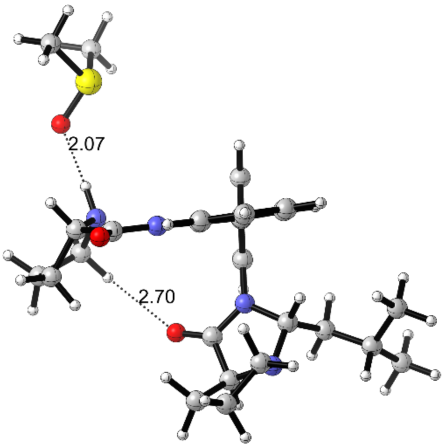

R$_a$-*exo*-**3**_c1

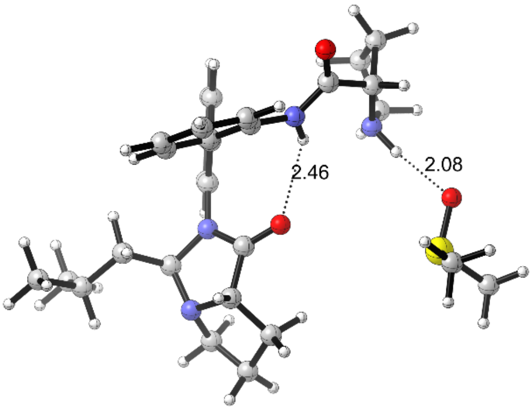

S$_a$-*exo*-**3**_c1

Figure S5. Comparison of the lowest energy (*R*$_a$)- and (*S*$_a$)-conformers of endo- and exo-**3** views along the biphenyl axis. Note that the opening angle in (*R*$_a$)-exo-**3**_c1 is significantly reduced compared to the other conformers.

5. Crystallographic analysis of endo-6 and exo-6

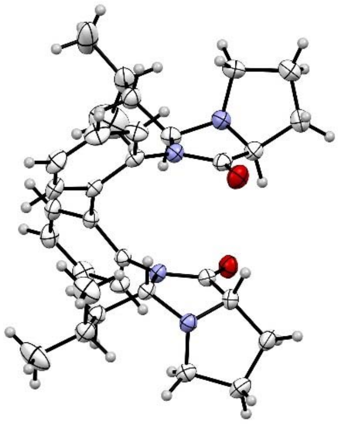

Table S15. Crystal data and structure refinement for endo-**6**.

CCDC-Nr	2166106
Empirical formula	C32 H42 N4 O2
Formula weight [g/mol]	514.7
Crystal system	Orthorhombic
Space group	$P2_12_12_1$ (19)
Lattice parameters [Å]	
a	9.65900(10)
b	9.87320(10)
c	29.9476(3)
α	90
β	90
γ	90
Density [g/cm3]	1.197
Crystal size [mm3]	0.253 x 0.218 x 0.159
Volume [Å3]	2855.96(5)
Z	4
Temperature [K]	169.99(10)
Diffraction Device	XtaLAB Synergy, Dualflex, HyPix
Radiation Type	1.54184 Å (Cu K/ micro-focus sealed X-ray tube)
F(000)	1112
Absorption coefficient [mm-1]	0.589
Absorption correction	Gaussian
Measurement range	3.0 - 66.5
Index range	-11 < h < 10
	-11 < k < 11
	-35 < l < 28
Measured reflexes	16297
Independent	5025
Observed	4884
R(int)	0.0305
Completeness (%) / theta (°)	100.0 / 66.500
Transmission (min / max)	0.484 / 1.000
R1 (observed/all)	0.0327 / 0.0335
wR2 (observed/all)	0.0833 / 0.0839
GooF = S	1.061
Rest electron density max./min. [e-/Å³]	-0.177 / 0.135

Table S16. Atomic coordinates and equivalent isotropic displacement parameters [Å^2] for endo-**6**

	x	y	z	U(eq)
O(1)	0.28448(14)	0.34554(15)	0.35063(5)	0.0362(3)
O(2)	0.45604(16)	0.67893(14)	0.36627(5)	0.0371(3)
N(1)	0.46903(15)	0.46076(16)	0.25571(5)	0.0254(3)
N(2)	0.50777(15)	0.34520(16)	0.32245(5)	0.0248(3)
N(3)	0.55973(16)	0.51946(16)	0.41155(5)	0.0257(3)
N(4)	0.39779(16)	0.51373(17)	0.46914(5)	0.0307(4)
C(1)	0.8674(3)	0.3272(3)	0.20080(9)	0.0515(6)
C(2)	0.7638(2)	0.4182(2)	0.22469(7)	0.0335(4)
C(3)	0.8337(3)	0.5474(2)	0.23992(10)	0.0480(6)
C(4)	0.69500(19)	0.3402(2)	0.26348(6)	0.0277(4)
C(5)	0.58111(18)	0.42102(19)	0.28593(6)	0.0246(4)
C(6)	0.4193(2)	0.3530(2)	0.22519(6)	0.0300(4)
C(7)	0.2733(2)	0.4007(2)	0.21395(7)	0.0352(4)
C(8)	0.2187(2)	0.4562(2)	0.25845(7)	0.0332(4)
C(9)	0.34994(18)	0.4831(2)	0.28577(6)	0.0272(4)
C(10)	0.37178(19)	0.38320(19)	0.32366(6)	0.0262(4)
C(11)	0.5648(2)	0.24690(18)	0.35183(6)	0.0258(4)
C(12)	0.4973(2)	0.1228(2)	0.35577(7)	0.0342(4)
C(13)	0.5487(3)	0.0223(2)	0.38296(7)	0.0423(5)
C(14)	0.6708(3)	0.0430(2)	0.40598(8)	0.0477(6)
C(15)	0.7381(3)	0.1651(2)	0.40256(7)	0.0407(5)
C(16)	0.6859(2)	0.27109(19)	0.37648(6)	0.0284(4)
C(17)	0.75936(19)	0.4040(2)	0.37716(6)	0.0274(4)
C(18)	0.8981(2)	0.4119(2)	0.36390(7)	0.0365(5)
C(19)	0.9688(2)	0.5328(3)	0.36387(7)	0.0417(5)
C(20)	0.9028(2)	0.6501(3)	0.37722(7)	0.0420(5)
C(21)	0.7663(2)	0.6447(2)	0.39181(6)	0.0339(4)
C(22)	0.69588(19)	0.5221(2)	0.39282(6)	0.0260(4)
C(23)	0.4541(2)	0.59963(19)	0.39764(6)	0.0279(4)
C(24)	0.3319(2)	0.5715(2)	0.42895(7)	0.0325(4)
C(25)	0.2558(3)	0.6986(3)	0.44397(8)	0.0462(6)
C(26)	0.3057(3)	0.7211(3)	0.49169(9)	0.0556(7)
C(27)	0.4343(2)	0.6343(2)	0.49575(7)	0.0404(5)
C(28)	0.5162(2)	0.4371(2)	0.45144(6)	0.0290(4)
C(29)	0.6318(2)	0.4158(2)	0.48491(7)	0.0343(5)
C(30)	0.5854(2)	0.3383(3)	0.52675(7)	0.0429(5)
C(31)	0.5646(3)	0.1880(3)	0.51761(10)	0.0621(8)
C(32)	0.6894(4)	0.3584(4)	0.56421(10)	0.0770(10)
H(1A)	0.818816	0.249343	0.187878	0.077
H(1B)	0.936901	0.295189	0.22218	0.077
H(1C)	0.913122	0.378445	0.176976	0.077
H(2)	0.689784	0.443173	0.202836	0.04
H(3A)	0.881709	0.589219	0.214614	0.072
H(3B)	0.900707	0.526363	0.263488	0.072

H(3C)	0.763839	0.610193	0.251526	0.072
H(4A)	0.655711	0.254556	0.251893	0.033
H(4B)	0.766393	0.316917	0.285921	0.033
H(5)	0.622745	0.504869	0.298988	0.03
H(6A)	0.417922	0.263673	0.240219	0.036
H(6B)	0.477631	0.347123	0.198072	0.036
H(7A)	0.215478	0.324618	0.203227	0.042
H(7B)	0.275163	0.472387	0.190833	0.042
H(8A)	0.165554	0.540757	0.253826	0.04
H(8B)	0.158656	0.388973	0.273501	0.04
H(9)	0.350156	0.578055	0.297353	0.033
H(12)	0.414539	0.107271	0.339429	0.041
H(13)	0.500334	-0.060967	0.385894	0.051
H(14)	0.708089	-0.027017	0.424108	0.057
H(15)	0.822399	0.178019	0.418341	0.049
H(18)	0.944462	0.331746	0.354695	0.044
H(19)	1.062942	0.535603	0.354679	0.05
H(20)	0.950554	0.734235	0.37643	0.05
H(21)	0.72099	0.72529	0.401144	0.041
H(24)	0.266278	0.50509	0.415232	0.039
H(25A)	0.154298	0.684783	0.443128	0.055
H(25B)	0.27983	0.776692	0.424744	0.055
H(26A)	0.327939	0.817731	0.496858	0.067
H(26B)	0.234435	0.69239	0.513469	0.067
H(27A)	0.516326	0.680935	0.483256	0.048
H(27B)	0.452859	0.60994	0.527259	0.048
H(28)	0.482814	0.346713	0.440918	0.035
H(29A)	0.66876	0.505175	0.494034	0.041
H(29B)	0.707812	0.365156	0.470319	0.041
H(30)	0.494805	0.376784	0.536703	0.051
H(31A)	0.498346	0.1766	0.493125	0.093
H(31B)	0.528836	0.143772	0.544523	0.093
H(31C)	0.653348	0.147007	0.509284	0.093
H(32A)	0.781043	0.328383	0.554261	0.115
H(32B)	0.660957	0.305209	0.590277	0.115
H(32C)	0.693193	0.454507	0.572255	0.115

Table S17. Anisotropic displacement parameters [Å^2] for endo-**6**

	U^{11}	U^{22}	U^{33}	U^{23}	U^{13}	U^{12}
O(1)	0.0294(7)	0.0453(8)	0.0339(7)	0.0043(6)	0.0080(6)	0.0000(6)
O(2)	0.0457(8)	0.0332(7)	0.0325(7)	0.0056(6)	-0.0042(7)	0.0042(7)
N(1)	0.0226(7)	0.0278(8)	0.0259(7)	0.0000(6)	-0.0010(6)	0.0008(6)
N(2)	0.0245(7)	0.0265(8)	0.0235(7)	0.0007(6)	-0.0003(6)	0.0002(6)
N(3)	0.0267(8)	0.0255(8)	0.0248(7)	0.0019(6)	0.0009(6)	0.0001(6)
N(4)	0.0265(7)	0.0367(9)	0.0289(8)	0.0018(7)	0.0003(6)	0.0024(7)
C(1)	0.0520(14)	0.0476(14)	0.0550(14)	-0.0096(12)	0.0257(12)	-0.0054(11)
C(2)	0.0258(9)	0.0412(11)	0.0336(10)	0.0050(9)	0.0043(8)	0.0019(8)
C(3)	0.0422(12)	0.0351(12)	0.0668(16)	0.0009(11)	0.0197(11)	-0.0024(10)
C(4)	0.0257(9)	0.0288(9)	0.0288(9)	0.0010(8)	0.0018(8)	0.0015(8)
C(5)	0.0237(8)	0.0250(9)	0.0251(8)	0.0019(7)	-0.0008(7)	-0.0010(7)
C(6)	0.0277(9)	0.0357(10)	0.0264(9)	-0.0025(8)	-0.0003(8)	-0.0028(8)
C(7)	0.0289(10)	0.0468(12)	0.0299(9)	0.0006(9)	-0.0049(8)	-0.0020(9)
C(8)	0.0230(9)	0.0409(11)	0.0359(10)	0.0024(9)	-0.0030(8)	0.0016(8)
C(9)	0.0231(8)	0.0295(9)	0.0290(9)	-0.0011(8)	0.0010(8)	0.0025(7)
C(10)	0.0256(9)	0.0268(9)	0.0263(9)	-0.0037(7)	0.0011(8)	-0.0007(7)
C(11)	0.0307(10)	0.0227(9)	0.0240(9)	-0.0006(7)	0.0030(8)	0.0034(7)
C(12)	0.0416(11)	0.0295(10)	0.0315(10)	-0.0047(8)	0.0021(9)	-0.0027(9)
C(13)	0.0640(15)	0.0250(10)	0.0379(11)	0.0014(8)	0.0049(11)	-0.0027(10)
C(14)	0.0737(16)	0.0291(11)	0.0403(12)	0.0071(9)	-0.0078(11)	0.0124(11)
C(15)	0.0484(12)	0.0365(11)	0.0373(11)	0.0022(9)	-0.0109(10)	0.0127(10)
C(16)	0.0306(10)	0.0278(9)	0.0268(9)	-0.0014(8)	-0.0002(8)	0.0062(8)
C(17)	0.0257(9)	0.0340(10)	0.0226(8)	0.0016(7)	-0.0047(7)	0.0018(8)
C(18)	0.0276(10)	0.0514(12)	0.0304(10)	-0.0018(9)	-0.0044(8)	0.0037(9)
C(19)	0.0274(10)	0.0660(15)	0.0316(10)	0.0037(10)	-0.0007(8)	-0.0073(10)
C(20)	0.0407(12)	0.0523(13)	0.0329(10)	0.0031(10)	-0.0012(9)	-0.0207(10)
C(21)	0.0384(11)	0.0355(10)	0.0278(9)	-0.0005(8)	0.0001(9)	-0.0077(9)
C(22)	0.0269(9)	0.0307(9)	0.0203(8)	0.0024(7)	-0.0018(7)	-0.0035(8)
C(23)	0.0325(10)	0.0257(9)	0.0254(9)	-0.0040(8)	-0.0044(8)	0.0021(8)
C(24)	0.0266(9)	0.0398(11)	0.0310(10)	-0.0030(9)	-0.0029(8)	0.0039(8)
C(25)	0.0437(13)	0.0545(14)	0.0404(12)	-0.0010(11)	0.0015(10)	0.0201(11)
C(26)	0.0620(16)	0.0600(16)	0.0447(13)	-0.0110(12)	-0.0030(12)	0.0245(14)
C(27)	0.0426(12)	0.0456(13)	0.0329(10)	-0.0071(9)	-0.0033(9)	0.0050(10)
C(28)	0.0280(9)	0.0299(10)	0.0291(9)	0.0047(8)	0.0019(8)	-0.0010(8)
C(29)	0.0269(9)	0.0430(11)	0.0330(10)	0.0087(9)	0.0023(8)	0.0035(9)
C(30)	0.0381(11)	0.0543(14)	0.0363(11)	0.0170(10)	0.0066(9)	0.0133(10)
C(31)	0.0695(18)	0.0583(17)	0.0583(16)	0.0255(13)	0.0118(14)	0.0036(14)
C(32)	0.086(2)	0.100(3)	0.0451(15)	0.0269(16)	-0.0145(16)	0.005(2)

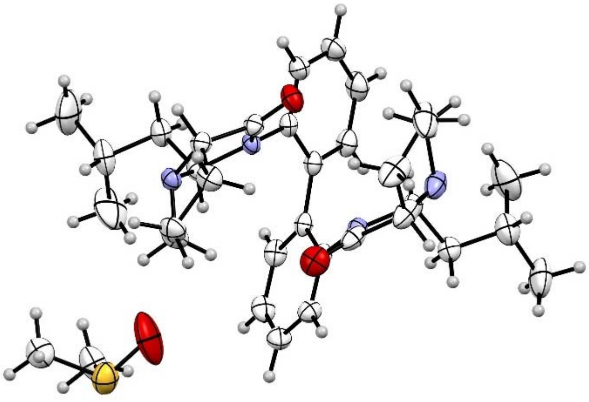

Table S18. Crystal data and structure refinement for exo-**6**.

CCDC-Nr	2168830
Empirical formula	C34 H48 N4 O4 S1
Formula weight [g/mol]	592.82
Crystal system	Orthorhombic
Space group	$P2_12_12_1$ (19)
Lattice parameters [Å]	
a	9.98620(10)
b	15.0653(2)
c	21.9578(3)
α	90
β	90
γ	90
Density [g/cm3]	1.192
Crystal size [mm3]	0.165 x 0.095 x 0.065
Volume [Å3]	3303.44(7)
Z	4
Temperature [K]	169.99(10)
Diffraction Device	XtaLAB Synergy, Dualflex, HyPix
Radiation Type	1.54184 Å (Cu K/ micro-focus sealed X-ray tube)
F(000)	1280
Absorption coefficient [mm-1]	1.17
Absorption correction	Gaussian
Measurement range	3.6 - 66.4
Index range	-10 < h < 11
	-14 < k < 17
	-25 < l < 26
Measured reflexes	18467
Independent	5809
Observed	5402
R(int)	0.0377
Completeness (%) / theta (°)	100.0 / 66.436
Transmission (min / max)	0.700 / 1.000
R1 (observed/all)	0.0388 / 0.0420
wR2 (observed/all)	0.1013 / 0.1034
GooF = S	1.047
Rest electron density max./min. [e-/Å³]	-0.252 / 0.437

Table S19. Atomic coordinates and equivalent isotropic displacement parameters [Å^2] for exo-**6**

	x	y	z	U(eq)
O(3)	0.7546(2)	0.56219(14)	0.55868(10)	0.0458(5)
N(1)	1.0402(2)	0.69583(18)	0.59063(12)	0.0432(6)
N(2)	0.8106(2)	0.69121(14)	0.60932(10)	0.0291(5)
O(2)	0.93473(18)	0.51037(12)	0.71513(10)	0.0376(5)
N(3)	0.7391(2)	0.58577(13)	0.73596(9)	0.0254(4)
N(4)	0.5822(2)	0.47393(13)	0.71948(10)	0.0284(4)
C(3)	0.8128(3)	0.51287(16)	0.72132(11)	0.0273(5)
C(4)	0.7179(3)	0.43569(16)	0.71444(12)	0.0292(5)
C(5)	0.7253(3)	0.38705(19)	0.65283(14)	0.0369(6)
C(6)	0.5793(3)	0.3851(2)	0.63180(14)	0.0418(7)
C(7)	0.5226(3)	0.4701(2)	0.65819(14)	0.0384(6)
C(8)	0.5955(2)	0.56328(16)	0.74519(12)	0.0277(5)
C(9)	0.5588(3)	0.56633(19)	0.81225(13)	0.0356(6)
C(10)	0.4112(3)	0.5461(2)	0.82568(16)	0.0469(8)
C(11)	0.3930(5)	0.5330(3)	0.8938(2)	0.0815(14)
C(12)	0.3182(4)	0.6168(3)	0.8001(3)	0.0860(15)
C(13)	0.7967(2)	0.66671(16)	0.75746(11)	0.0261(5)
C(14)	0.8926(3)	0.66394(18)	0.80337(13)	0.0340(6)
C(15)	0.9464(3)	0.7417(2)	0.82678(13)	0.0396(6)
C(16)	0.9039(3)	0.82269(19)	0.80453(14)	0.0408(7)
C(17)	0.8088(3)	0.82616(17)	0.75880(12)	0.0336(6)
C(18)	0.7549(2)	0.74872(16)	0.73392(11)	0.0266(5)
C(19)	0.6546(2)	0.75411(15)	0.68411(11)	0.0257(5)
C(20)	0.5303(3)	0.79382(17)	0.69557(13)	0.0321(6)
C(21)	0.4323(3)	0.79598(18)	0.65169(14)	0.0367(6)
C(22)	0.4551(3)	0.75967(19)	0.59483(13)	0.0361(6)
C(23)	0.5796(3)	0.72413(17)	0.58131(12)	0.0320(6)
C(24)	0.6799(3)	0.72200(15)	0.62525(11)	0.0266(5)
C(25)	0.9294(2)	0.75098(19)	0.61210(12)	0.0326(5)
C(26)	0.8347(3)	0.61887(19)	0.57366(12)	0.0350(6)
C(27)	0.9072(3)	0.83337(17)	0.57353(13)	0.0337(6)
C(28)	1.0251(3)	0.8961(2)	0.57232(17)	0.0490(8)
C(29)	1.0645(5)	0.9275(3)	0.6343(2)	0.0725(12)
C(30)	0.9917(4)	0.9746(3)	0.5301(2)	0.0687(11)
C(31)	0.9815(3)	0.6221(2)	0.55571(14)	0.0431(7)
C(32)	1.0613(4)	0.5378(2)	0.57171(17)	0.0557(9)
C(33)	1.1742(4)	0.5718(3)	0.6108(2)	0.0694(12)
C(34)	1.1166(3)	0.6539(2)	0.6407(2)	0.0560(10)
S(1)	0.32477(9)	0.25224(6)	0.48576(4)	0.0523(2)
O(1)	0.4731(3)	0.2522(3)	0.48908(19)	0.0973(12)
C(1)	0.2682(3)	0.3284(2)	0.54150(19)	0.0556(9)
C(2)	0.2684(4)	0.1542(2)	0.52266(18)	0.0515(8)
H(4)	0.732765	0.392384	0.748282	0.035
H(5A)	0.782226	0.419864	0.623555	0.044

H(5B)	0.761174	0.326217	0.657827	0.044
H(6A)	0.57309	0.384916	0.586801	0.05
H(6B)	0.53202	0.332457	0.648053	0.05
H(7A)	0.549335	0.52219	0.633535	0.046
H(7B)	0.423662	0.467599	0.660424	0.046
H(8)	0.537968	0.605719	0.721897	0.033
H(9A)	0.580267	0.626084	0.828272	0.043
H(9B)	0.615175	0.522966	0.834418	0.043
H(10)	0.388632	0.488663	0.805247	0.056
H(11A)	0.299443	0.517738	0.902395	0.122
H(11B)	0.416338	0.587961	0.915208	0.122
H(11C)	0.451357	0.48486	0.907839	0.122
H(12A)	0.325947	0.618028	0.75565	0.129
H(12B)	0.343102	0.674838	0.816738	0.129
H(12C)	0.225655	0.602963	0.811548	0.129
H(14)	0.921621	0.60827	0.818827	0.041
H(15)	1.012154	0.739216	0.857991	0.048
H(16)	0.940099	0.876079	0.820643	0.049
H(17)	0.779568	0.882206	0.744077	0.04
H(20)	0.513486	0.81965	0.734291	0.039
H(21)	0.348209	0.822592	0.660494	0.044
H(22)	0.385891	0.759086	0.565164	0.043
H(23)	0.596614	0.701077	0.541791	0.038
H(25)	0.946033	0.768843	0.655309	0.039
H(27A)	0.886761	0.814782	0.531301	0.04
H(27B)	0.828126	0.865582	0.589368	0.04
H(28)	1.103107	0.86367	0.554412	0.059
H(29A)	1.139586	0.969101	0.63087	0.109
H(29B)	1.091534	0.876572	0.659223	0.109
H(29C)	0.988307	0.9574	0.653538	0.109
H(30A)	1.071754	1.01122	0.524366	0.103
H(30B)	0.920564	1.01049	0.548524	0.103
H(30C)	0.961395	0.951961	0.490607	0.103
H(31)	0.989441	0.63455	0.511118	0.052
H(32A)	1.005058	0.494994	0.594427	0.067
H(32B)	1.096044	0.508677	0.534517	0.067
H(33A)	1.253352	0.586778	0.585725	0.083
H(33B)	1.200347	0.527218	0.641732	0.083
H(34A)	1.057235	0.638083	0.675097	0.067
H(34B)	1.188509	0.693595	0.655552	0.067
H(1A)	0.299889	0.388168	0.531246	0.083
H(1B)	0.303282	0.311229	0.581474	0.083
H(1C)	0.170089	0.328122	0.542639	0.083
H(2A)	0.170822	0.156387	0.527237	0.077
H(2B)	0.310184	0.149709	0.562926	0.077
H(2C)	0.293041	0.102286	0.498178	0.077

Table S20. Anisotropic displacement parameters [Å^2] for exo-**6**

	U11	U22	U33	U23	U13	U12
O(3)	0.0542(13)	0.0327(10)	0.0506(12)	-0.0062(9)	0.0039(10)	0.0062(10)
N(1)	0.0336(12)	0.0448(14)	0.0513(15)	0.0179(12)	0.0140(11)	0.0106(11)
N(2)	0.0265(10)	0.0313(11)	0.0294(11)	0.0033(9)	0.0055(9)	0.0033(9)
O(2)	0.0265(10)	0.0313(9)	0.0551(12)	-0.0029(9)	0.0030(9)	0.0003(7)
N(3)	0.0248(10)	0.0206(9)	0.0307(11)	0.0007(8)	0.0002(8)	-0.0024(8)
N(4)	0.0277(10)	0.0240(10)	0.0336(11)	-0.0008(9)	-0.0012(9)	-0.0043(9)
C(3)	0.0297(13)	0.0231(12)	0.0292(13)	0.0022(9)	0.0004(10)	0.0007(10)
C(4)	0.0327(13)	0.0228(12)	0.0321(13)	0.0017(10)	-0.0017(10)	-0.0013(10)
C(5)	0.0405(15)	0.0284(13)	0.0420(15)	-0.0083(11)	-0.0026(12)	0.0009(12)
C(6)	0.0423(15)	0.0423(15)	0.0408(16)	-0.0080(13)	-0.0058(13)	-0.0082(14)
C(7)	0.0331(14)	0.0428(15)	0.0394(15)	-0.0047(12)	-0.0071(12)	0.0002(12)
C(8)	0.0231(11)	0.0223(11)	0.0377(14)	0.0009(10)	-0.0009(10)	-0.0030(9)
C(9)	0.0334(15)	0.0329(14)	0.0406(15)	-0.0054(11)	0.0067(12)	-0.0066(11)
C(10)	0.0375(15)	0.0384(15)	0.065(2)	-0.0110(14)	0.0191(15)	-0.0112(13)
C(11)	0.076(3)	0.094(3)	0.074(3)	-0.007(2)	0.044(2)	-0.020(3)
C(12)	0.0373(19)	0.087(3)	0.133(4)	0.005(3)	0.020(2)	0.017(2)
C(13)	0.0257(12)	0.0240(12)	0.0288(12)	-0.0010(9)	0.0043(10)	-0.0046(10)
C(14)	0.0341(14)	0.0314(13)	0.0365(15)	0.0016(11)	-0.0036(11)	-0.0059(11)
C(15)	0.0375(15)	0.0435(15)	0.0379(14)	-0.0046(13)	-0.0054(11)	-0.0095(13)
C(16)	0.0476(16)	0.0326(14)	0.0423(16)	-0.0095(12)	0.0033(13)	-0.0145(13)
C(17)	0.0414(15)	0.0233(12)	0.0362(14)	-0.0037(10)	0.0064(12)	-0.0044(11)
C(18)	0.0278(11)	0.0236(11)	0.0284(11)	-0.0018(10)	0.0070(9)	-0.0018(10)
C(19)	0.0297(12)	0.0172(10)	0.0301(12)	0.0006(9)	0.0045(9)	-0.0018(10)
C(20)	0.0348(13)	0.0242(12)	0.0373(14)	-0.0033(10)	0.0084(11)	0.0022(11)
C(21)	0.0297(13)	0.0307(13)	0.0495(17)	0.0010(12)	0.0053(12)	0.0056(11)
C(22)	0.0325(14)	0.0355(14)	0.0404(14)	0.0048(12)	-0.0026(11)	0.0026(11)
C(23)	0.0335(13)	0.0327(13)	0.0297(13)	0.0011(10)	0.0013(11)	0.0028(11)
C(24)	0.0282(12)	0.0206(11)	0.0309(12)	0.0041(9)	0.0042(10)	0.0006(9)
C(25)	0.0248(12)	0.0411(14)	0.0318(12)	0.0040(12)	0.0044(10)	-0.0013(12)
C(26)	0.0438(16)	0.0316(14)	0.0297(13)	0.0067(11)	0.0079(12)	0.0122(13)
C(27)	0.0333(13)	0.0297(13)	0.0382(15)	-0.0019(11)	0.0021(12)	0.0005(11)
C(28)	0.0410(16)	0.0432(17)	0.063(2)	-0.0016(15)	0.0071(15)	-0.0071(14)
C(29)	0.066(2)	0.060(2)	0.092(3)	-0.030(2)	0.003(2)	-0.025(2)
C(30)	0.067(2)	0.0453(19)	0.094(3)	0.0155(19)	0.020(2)	-0.0155(18)
C(31)	0.0474(17)	0.0420(16)	0.0397(16)	0.0095(13)	0.0173(14)	0.0175(14)
C(32)	0.060(2)	0.0490(18)	0.059(2)	0.0126(16)	0.0181(17)	0.0286(16)
C(33)	0.0329(16)	0.060(2)	0.116(3)	0.037(2)	0.014(2)	0.0102(16)
C(34)	0.0259(15)	0.056(2)	0.086(3)	0.0277(18)	-0.0110(15)	-0.0044(13)
S(1)	0.0540(5)	0.0568(5)	0.0460(4)	-0.0028(4)	0.0118(3)	-0.0053(4)
O(1)	0.0490(14)	0.096(2)	0.146(3)	-0.035(2)	0.0487(18)	-0.0176(16)
C(1)	0.0417(17)	0.0501(19)	0.075(2)	-0.0127(17)	0.0064(16)	-0.0063(15)
C(2)	0.0474(18)	0.0450(17)	0.062(2)	-0.0025(15)	-0.0001(16)	0.0008(14)

6. ^{1}H-NMR spectra of catalyst 1

6.1 Pure catalyst

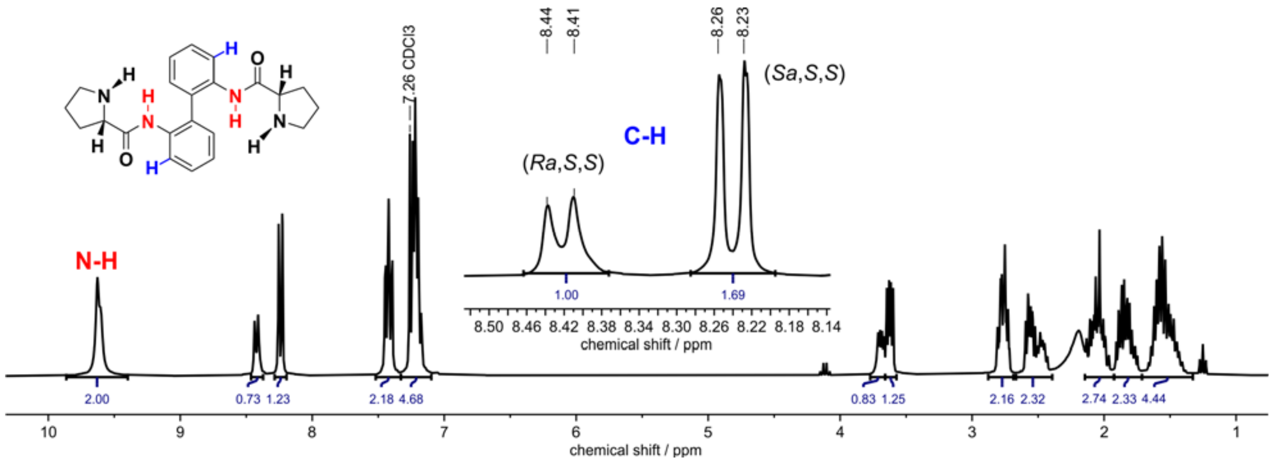

Figure S6. 300 MHz ^{1}H-NMR of **1** in $CDCl_3$

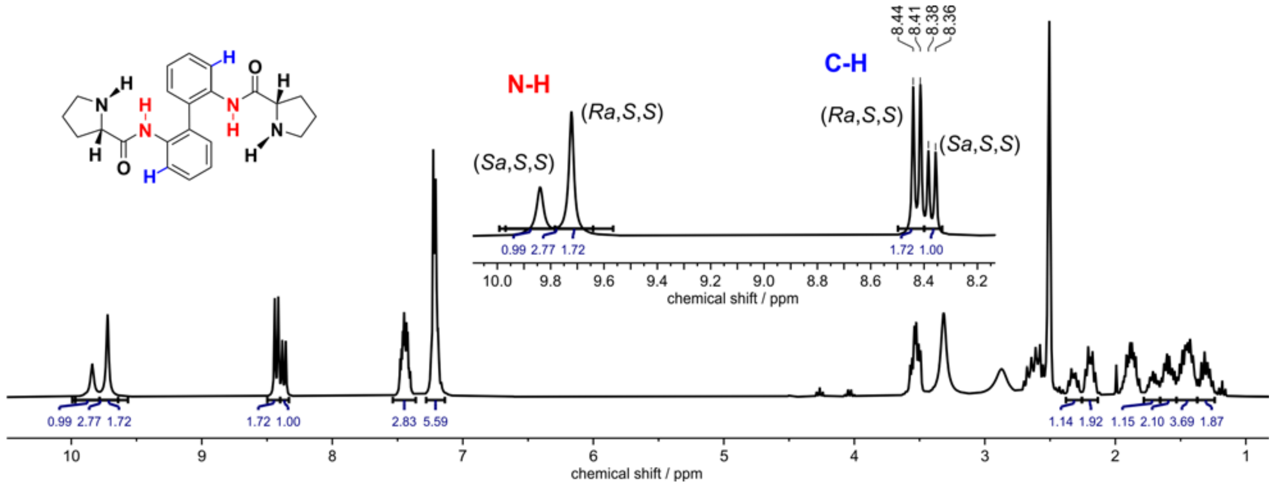

Figure S7. 300 MHz ^{1}H-NMR of **1** in DMSO-d_6

6.2 300 MHz ^{1}H-NMR reaction monitoring of 1:1 mixture in DMSO-d_6

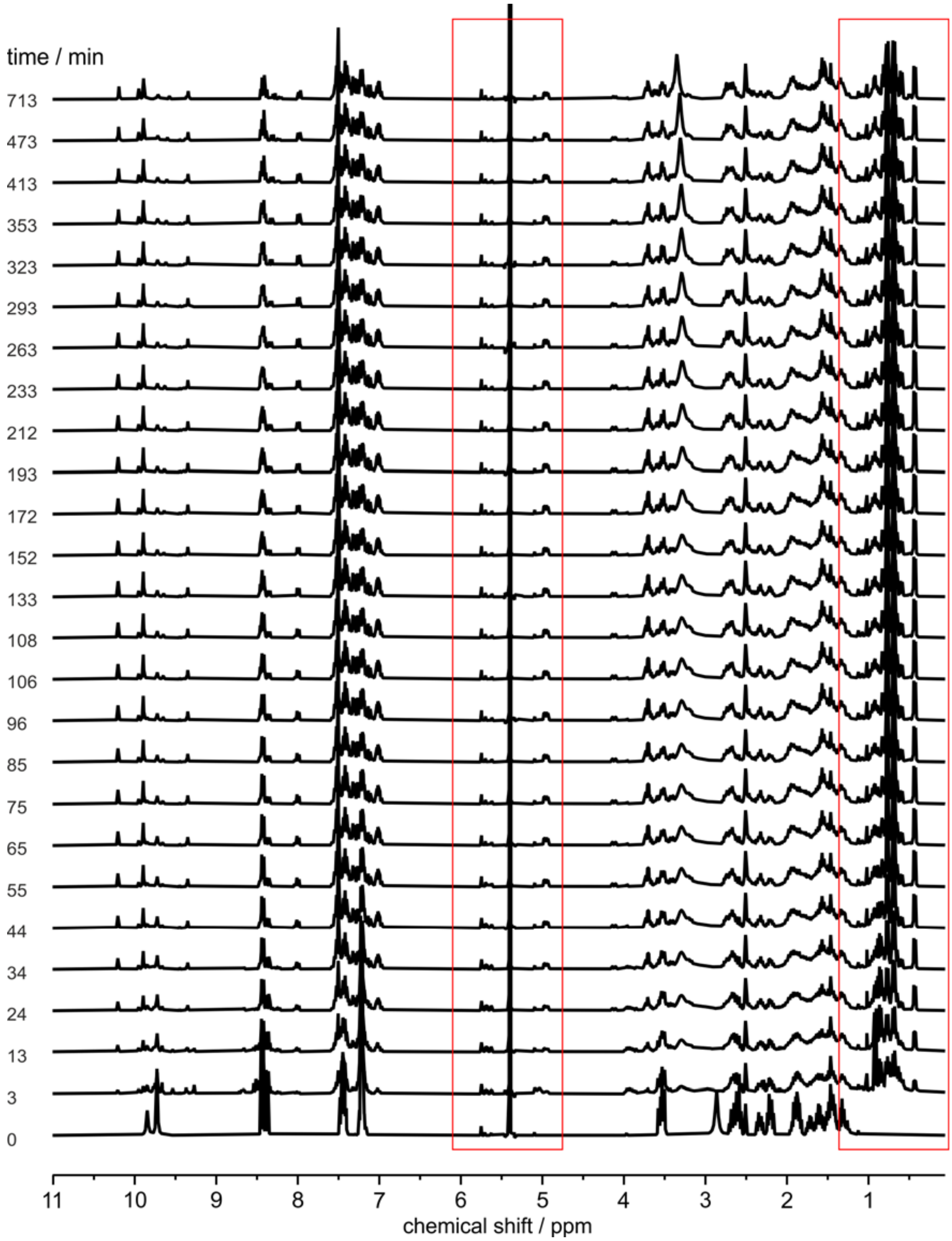

Figure S8. Overview of the ^{1}H-NMR spectra recorded during the reaction monitoring of the 1:1 mixture.

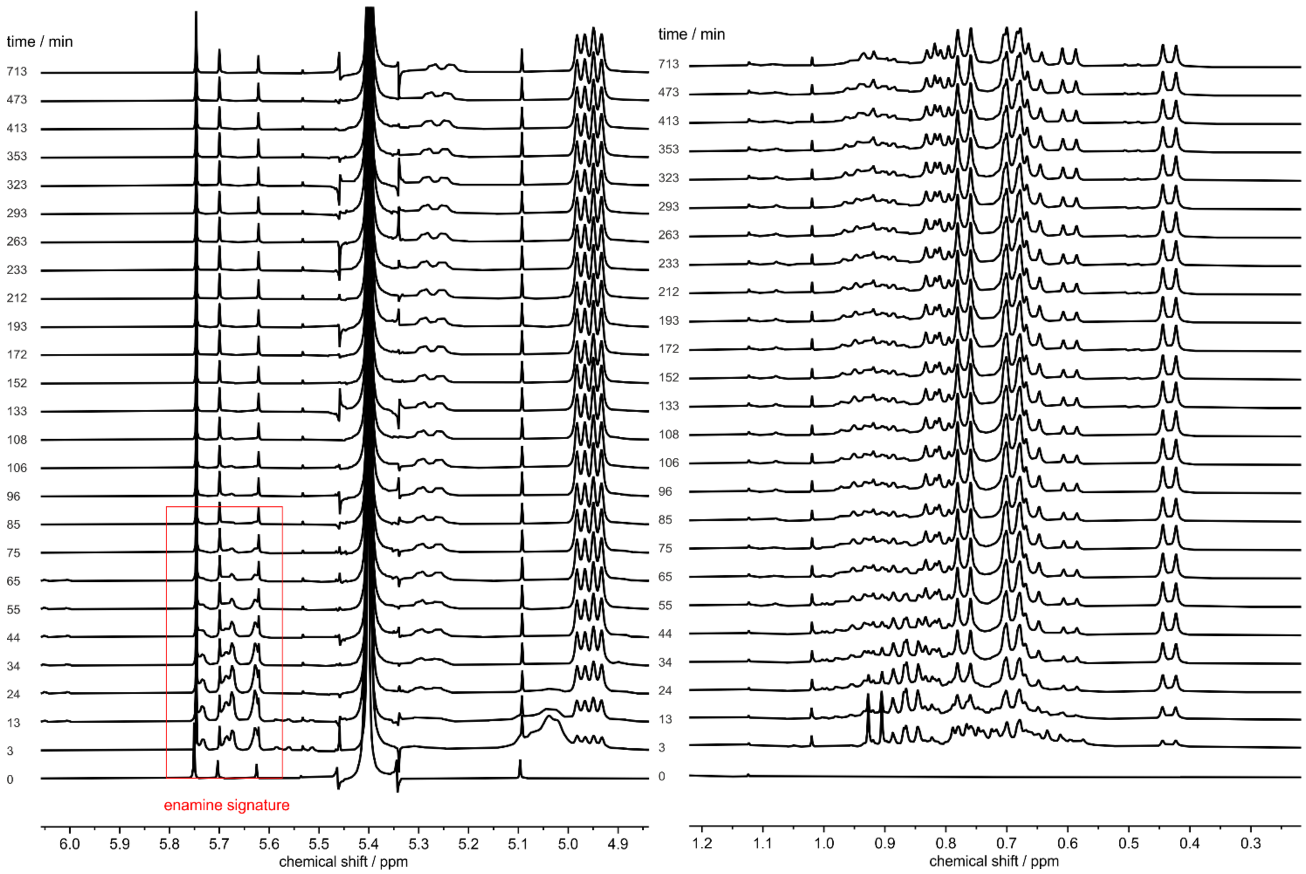

Figure S9. Enlarged views of the spectra shown in Figure S8 for the enamine and reference signal region (left) and of the methyl group region (right).

6.3 300 MHz ^{1}H-NMR reaction monitoring of 1:2 mixture in DMSO-d_6

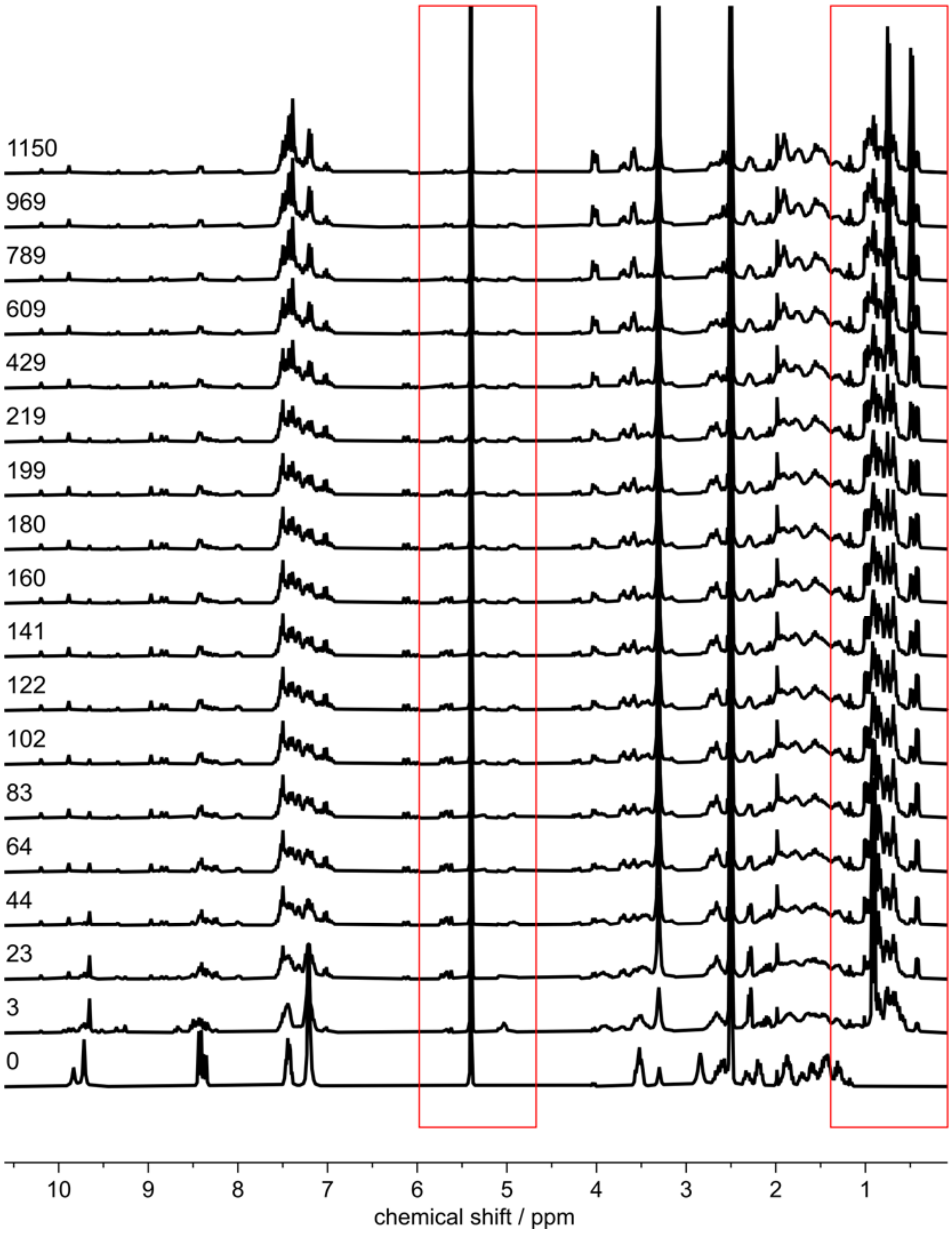

Figure S10. Overview of the ^{1}H-NMR spectra recorded during the reaction monitoring of the 1:2 mixture.

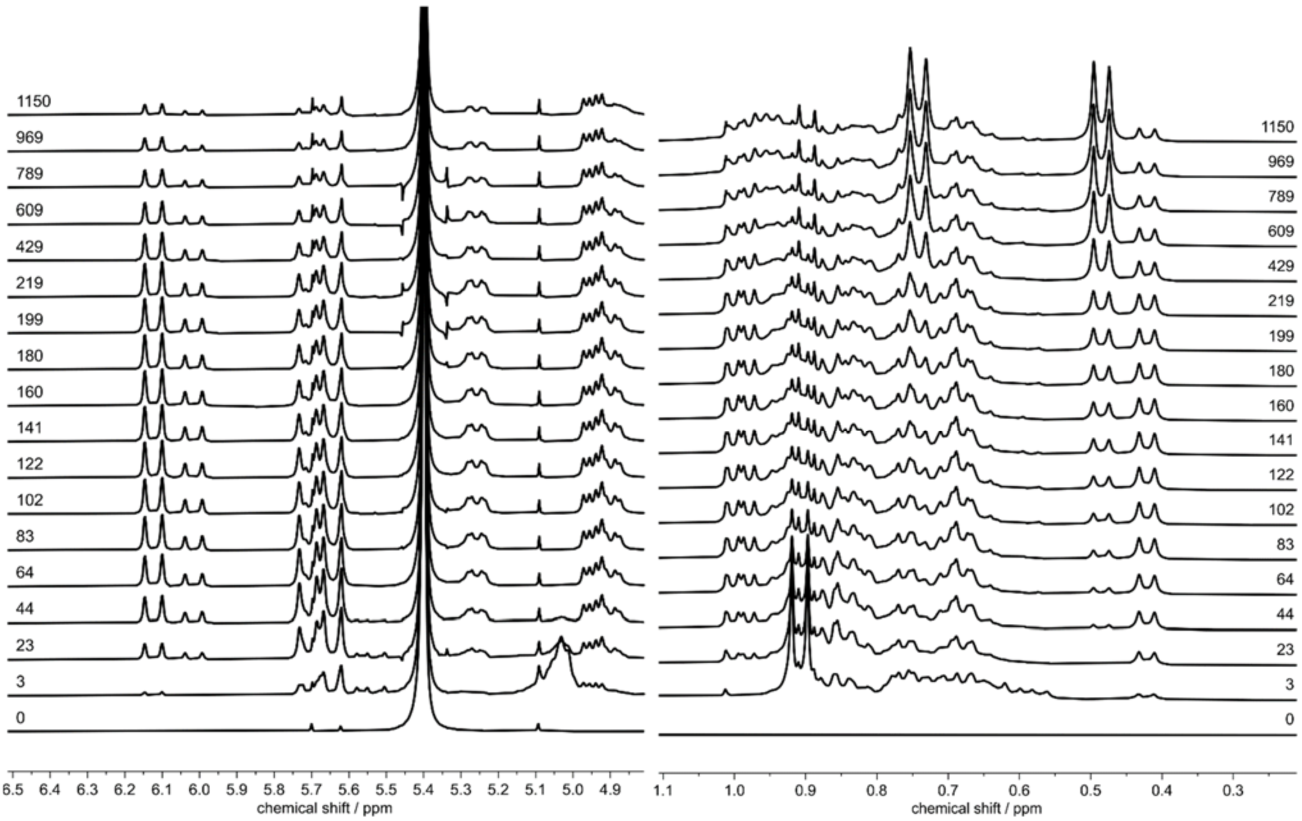

Figure S11. Enlarged views of the spectra shown in Figure S10 for the enamine and reference signal region (left) and of the methyl group region (right).

7. Cartesian coordinates

S_a-1_c1

C	0.09624900	-0.81593500	-1.93438200
C	-0.53283500	-1.61098700	-0.94675700
C	-0.33498000	-2.99989400	-0.94091800
C	0.47187300	-3.59648700	-1.90757300
C	1.08754300	-2.82630300	-2.89319800
C	0.89243700	-1.44573000	-2.89796500
C	-0.09431500	0.66695400	-1.98960700
C	-0.88905400	1.22344700	-2.99845100
C	-1.08472700	2.60047000	-3.09575500
C	-0.47115700	3.44163600	-2.16851500
C	0.33454500	2.91845000	-1.15933700
C	0.53304800	1.53305900	-1.06262500
H	-0.81778200	-3.59583900	-0.18020800
H	0.61220000	-4.67267600	-1.88803600
H	1.71332800	-3.29056200	-3.64803800
H	1.37069400	-0.83234900	-3.65526900
H	-1.36575300	0.55555200	-3.70919600
H	-1.70947600	3.00751300	-3.88374800
H	-0.61210100	4.51623700	-2.22866000
H	0.81579700	3.56947300	-0.44409100
N	1.37171400	0.96404600	-0.08936200
H	1.63326800	-0.01732400	-0.18932600
N	-1.37143200	-0.97120800	-0.01853200
H	-1.62549100	0.00280800	-0.18667300
C	1.87604300	1.50922500	1.05051700
C	-1.88505400	-1.43348100	1.15343900
O	-1.68618500	-2.54729200	1.63300200
O	1.66438200	2.65081700	1.45300800
C	-2.78386400	-0.42305100	1.87681500
C	-4.25207000	-0.92782400	1.95310100
H	-2.37197300	-0.33482000	2.88865500
C	-4.26092600	1.24907000	0.97167700
C	-5.07183300	0.36999600	1.93345200
H	-4.42122500	-1.54550100	2.83656100
H	-4.48720500	-1.53662200	1.07260900
H	-4.41534200	2.32141800	1.11529300
H	-4.52089100	1.01315800	-0.06823400
H	-5.09474000	0.82287400	2.93200100
H	-6.10400700	0.21542400	1.60590600
C	2.78254600	0.55919000	1.84278100
C	4.24675400	1.07954500	1.88142200
H	2.37213100	0.53949300	2.85889000
C	4.27308600	-1.16264900	1.05996300
C	5.07637000	-0.21000300	1.95581200
H	4.41136500	1.76107700	2.71752600
H	4.47727100	1.62427200	0.95870300
H	4.43462500	-2.22046600	1.28202500
H	4.53332000	-1.00180400	0.00591900
H	5.10194600	-0.58916200	2.98454500
H	6.10758900	-0.07162200	1.61817600
N	2.85150000	-0.80563500	1.27763000
H	2.41125500	-1.46838400	1.90401600
N	-2.84211300	0.89941400	1.21748200
H	-2.40011700	1.60110800	1.79867300

R_a-1_c1

C	0.08651500	-2.05311900	-0.74396400
C	-0.69947300	-1.22517900	-1.58057400
C	-0.51863000	-1.27277500	-2.97183800
C	0.42809000	-2.13247300	-3.52549300
C	1.20644600	-2.95428800	-2.71221600
C	1.02817200	-2.90552600	-1.32998400
C	-0.08652000	-2.05311700	0.74396900
C	-1.02817900	-2.90552000	1.32999300
C	-1.20645300	-2.95427700	2.71222400
C	-0.42809600	-2.13246100	3.52549800
C	0.51862600	-1.27276700	2.97184100
C	0.69946900	-1.22517600	1.58057700
H	-1.12391600	-0.63740100	-3.60116800
H	0.55150500	-2.15637800	-4.60372800
H	1.94098100	-3.62585100	-3.14386800
H	1.62535500	-3.54035500	-0.68247900

H	-1.62536300	-3.54035000	0.68248900
H	-1.94098900	-3.62583800	3.14387800
H	-0.55151100	-2.15636300	4.60373400
H	1.12391300	-0.63739200	3.60116900
N	1.64558200	-0.38372900	0.97175700
H	1.75133100	-0.43505100	-0.04203400
N	-1.64558400	-0.38372900	-0.97175700
H	-1.75133300	-0.43504700	0.04203500
C	2.51513500	0.50377700	1.52700600
C	-2.51513600	0.50377700	-1.52700800
O	-2.63088600	0.74118000	-2.72706600
O	2.63088600	0.74118300	2.72706400
C	-3.38830600	1.24064200	-0.50346700
C	-3.09701800	2.76814200	-0.50757100
H	-4.42231700	1.06161800	-0.81935500
C	-2.84027900	1.96271900	1.72572700
C	-3.35488700	3.18165500	0.94839500
H	-3.71750000	3.29570200	-1.23375800
H	-2.04945200	2.95094100	-0.77233200
H	-3.27561200	1.85575100	2.72233900
H	-1.75108100	2.02005900	1.84517700
H	-4.42914200	3.31595400	1.12360200
H	-2.84668300	4.11079500	1.22231600
C	3.38830900	1.24063600	0.50346400
C	3.09702800	2.76813700	0.50756400
H	4.42231900	1.06160700	0.81935300
C	2.84028500	1.96270900	-1.72573200
C	3.35489800	3.18164500	-0.94840400
H	3.71751200	3.29569600	1.23374900
H	2.04946300	2.95094100	0.77232500
H	3.27561700	1.85573600	-2.72234400
H	1.75108700	2.02005300	-1.84518200
H	4.42915300	3.31593900	-1.12361100
H	2.84669800	4.11078600	-1.22232700
N	3.18338200	0.78808600	-0.88993000
H	4.02342000	0.34274700	-1.23866600
N	-3.18338000	0.78809600	0.88992800
H	-4.02342100	0.34276000	1.23866500

S_a-1_c1-2dmso

C	1.28457800	2.62541900	-0.67883400
C	0.83046500	1.88298800	-1.79513100
C	1.66990500	1.72447200	-2.90963700
C	2.94182400	2.29405700	-2.91712000
C	3.40013100	3.02641300	-1.82310100
C	2.56697400	3.18381300	-0.71577700
C	0.41650900	2.85341300	0.52077700
C	-0.48429300	3.92404600	0.52463800
C	-1.28960500	4.19636300	1.63022500
C	-1.19216900	3.38099700	2.75675600
C	-0.30549900	2.30606900	2.78257600
C	0.50485100	2.03072700	1.66926000
H	1.31454800	1.15658800	-3.75655800
H	3.57474600	2.16064300	-3.78886000
H	4.38913600	3.47195500	-1.82909400
H	2.90825800	3.75459200	0.14245200
H	-0.54529600	4.55279200	-0.35840100
H	-1.97796000	5.03449700	1.61016000
H	-1.80851200	3.57785100	3.62836300
H	-0.22992300	1.67410400	3.65488100
N	1.41667100	0.96329700	1.64760800
H	1.98874400	0.82995400	0.81136300
N	-0.45995700	1.33205100	-1.74066000
H	-1.01442500	1.47734700	-0.89486200
C	1.67660800	0.00935900	2.58003000
C	-1.13781200	0.58860800	-2.65429700
O	-0.70836300	0.23575700	-3.75344500
O	1.11587300	-0.09934300	3.67135100
C	-2.55382900	0.20488100	-2.21035400
C	-3.62527500	0.84494100	-3.14056500
H	-2.59936100	-0.88746600	-2.29033500
C	-4.10666300	1.46714300	-0.88609000
C	-4.81123600	1.08843300	-2.19644500
H	-3.86308900	0.20077900	-3.98907100
H	-3.25861400	1.79806200	-3.53856200
H	-4.71434900	1.29457200	0.00638000

H	-3.83177900	2.53012000	-0.89974800
H	-5.38762700	0.16552100	-2.06145700
H	-5.49011500	1.86546300	-2.56012000
C	2.77908800	-0.97239600	2.16689600
C	4.00340600	-0.87614600	3.12414200
H	2.32654700	-1.96809200	2.24309500
C	4.75705700	-0.53117600	0.88473600
C	5.19498300	-1.18094700	2.20490300
H	3.91316700	-1.56144400	3.96898000
H	4.08835000	0.13940700	3.52739300
H	5.24783400	-0.95071600	0.00249000
H	4.97372900	0.54518400	0.90503000
H	5.30831500	-2.26344400	2.07216200
H	6.13902400	-0.78282400	2.58868700
N	3.29588100	-0.74828000	0.80204900
H	3.09597300	-1.56321400	0.22306400
N	-2.88519300	0.63322500	-0.83629700
H	-3.04461700	-0.18695100	-0.25372900
C	-3.33809900	-1.36190800	3.26982600
H(Iso=2)	-3.79150000	-1.66637000	4.21631800
H(Iso=2)	-3.67944600	-0.36945100	2.96738700
H(Iso=2)	-2.25023300	-1.38972900	3.34969200
S	-3.80102300	-2.57087000	1.99244200
C	-5.59124300	-2.24861200	1.98545000
H(Iso=2)	-6.02251300	-2.88489000	1.21080000
H(Iso=2)	-5.99867500	-2.51958600	2.96249200
H(Iso=2)	-5.76397000	-1.19336600	1.76172100
O	-3.26204400	-2.03057700	0.66822900
C	1.99881300	-3.80287200	-3.37080400
H(Iso=2)	1.11006600	-3.93928700	-3.99167300
H(Iso=2)	2.57959800	-4.72536600	-3.29963700
H(Iso=2)	2.60949000	-2.98927500	-3.76563300
S	1.47206600	-3.30128700	-1.70445000
C	0.58841200	-4.82561300	-1.25524700
H(Iso=2)	0.24529300	-4.69898200	-0.22712300
H(Iso=2)	-0.26764200	-4.94397300	-1.92398600
H(Iso=2)	1.27454700	-5.67197400	-1.33428400
O	2.73909700	-3.27776300	-0.84706300

R_a-1-_c1-2dmso

C	0.31605800	1.73745500	0.41123400
C	-0.14061800	1.02665600	1.54702900
C	0.47223100	1.24196700	2.79192800
C	1.52215200	2.15115400	2.90899900
C	1.97838600	2.85862800	1.79794600
C	1.37074500	2.64419500	0.56094900
C	-0.32012600	1.56196000	-0.93361500
C	-1.37990300	2.39456800	-1.30859000
C	-1.98639400	2.28474100	-2.55983200
C	-1.52410300	1.32131900	-3.45494900
C	-0.46905300	0.47804600	-3.11080200
C	0.14300500	0.58938900	-1.85204200
H	0.11650300	0.69588000	3.65296400
H	1.98163700	2.30433400	3.88045100
H	2.79321300	3.56882700	1.88920200
H	1.71486300	3.18744100	-0.31377200
H	-1.72875600	3.13987700	-0.60043400
H	-2.80531300	2.94343200	-2.82842400
H	-1.98296900	1.22000600	-4.43346300
H	-0.10861200	-0.26639700	-3.80499000
N	1.21517800	-0.22842300	-1.46036400
H	1.64535000	-0.06173000	-0.54846100
N	-1.20806200	0.13015100	1.37768800
H	-1.64457500	0.06236500	0.45608300
C	1.84145100	-1.23610400	-2.12361100
C	-1.82100200	-0.68719500	2.27413100
O	-1.53404400	-0.78631700	3.46774200
O	1.56510500	-1.62916700	-3.25764800
C	-2.94843600	-1.53112000	1.66891600
C	-2.61716700	-3.05059500	1.75138800
H	-3.83508400	-1.31145400	2.27504800
C	-3.06346100	-2.48859300	-0.52645100
C	-3.27090200	-3.62403200	0.48576100
H	-2.98543500	-3.49655000	2.67714800
H	-1.53243500	-3.20290700	1.71751700
H	-3.77377100	-2.50305800	-1.35736000

H	-2.05292200	-2.54343500	-0.95228400
H	-4.34209700	-3.79021800	0.65042500
H	-2.82276700	-4.57055700	0.16917500
C	2.97140200	-1.89335700	-1.32253000
C	2.65900900	-3.39291800	-1.03861000
H	3.86246300	-1.81344200	-1.95605100
C	3.05622900	-2.28660400	1.04042500
C	3.29216600	-3.63204200	0.33992000
H	3.05189200	-4.04432400	-1.82145400
H	1.57567300	-3.55006400	-0.98855100
H	3.74811500	-2.09001200	1.86365300
H	2.03677200	-2.24687900	1.44629900
H	4.36769000	-3.82015700	0.23967200
H	2.84512800	-4.47819700	0.87020000
N	3.21819700	-1.25933700	-0.01188200
H	4.16522800	-0.88323400	0.01366500
N	-3.21553800	-1.23621800	0.24683400
H	-4.16437000	-0.87766600	0.14571600
C	-7.77569500	1.14825200	-1.50260700
H(Iso=2)	-8.12532700	2.17094500	-1.66304900
H(Iso=2)	-8.58738100	0.49919000	-1.16614200
H(Iso=2)	-7.32239800	0.76023800	-2.41627900
S	-6.46802600	1.17071300	-0.23942800
C	-7.48223300	1.73670500	1.15978000
H(Iso=2)	-6.83035300	1.75007000	2.03487300
H(Iso=2)	-7.84286800	2.74580000	0.94575600
H(Iso=2)	-8.31149700	1.04042600	1.30437600
O	-6.12962700	-0.29339700	0.04641700
C	7.14554000	1.70493600	1.56701500
H(Iso=2)	7.50767300	2.73319100	1.49224400
H(Iso=2)	7.94972200	1.01938300	1.84397400
H(Iso=2)	6.32414200	1.64841400	2.28324500
S	6.46886600	1.20803800	-0.04606600
C	8.01378500	1.29580300	-1.00070600
H(Iso=2)	7.77654000	0.96824500	-2.01422800
H(Iso=2)	8.35904200	2.33250400	-1.01280000
H(Iso=2)	8.75437500	0.63591500	-0.54301900
O	6.12889900	-0.27845900	0.07406300

S_a-2_c1

C	-0.34308300	0.89619200	2.50592200
C	-0.37115500	-0.50440400	2.31135400
C	-1.40868500	-1.26338200	2.87500700
C	-2.40617100	-0.64076900	3.62289500
C	-2.38967900	0.73867000	3.82186100
C	-1.35889600	1.49217000	3.26172800
C	0.75186600	1.75272700	1.94779300
C	1.92616100	1.94224500	2.68451100
C	2.95037300	2.76914700	2.22282200
C	2.79546000	3.42243500	1.00114400
C	1.63781200	3.25187900	0.24409700
C	0.60861300	2.41575100	0.70509900
H	-1.42275900	-2.33183800	2.72203500
H	-3.19879100	-1.24558500	4.05184700
H	-3.16458900	1.22393700	4.40551300
H	-1.33101400	2.56728700	3.40958400
H	2.02776800	1.43437900	3.63875300
H	3.85055900	2.90314800	2.81300300
H	3.57942100	4.07335100	0.62704500
H	1.51755900	3.75737900	-0.70255500
N	-0.57551900	2.20883700	-0.01848700
H	-1.28950100	1.59177300	0.37624300
N	0.67172400	-1.08968000	1.56691800
H	1.42524600	-0.47560500	1.26975000
C	-0.96217400	2.68237300	-1.23117200
C	0.83346000	-2.37614000	1.16059900
O	0.02973500	-3.28768600	1.35103000
O	-0.30823600	3.43998700	-1.94848400
C	2.15676600	-2.67634200	0.43524500
C	3.12477500	-3.46285200	1.35797900
H	1.87614400	-3.28496800	-0.43075700
C	4.24938100	-1.47086000	0.65448000
C	4.09506500	-2.38708300	1.86961300
H	3.66447000	-4.20108500	0.75707700
H	2.59796400	-3.99675700	2.15100900

H	5.00464700	-1.86694600	-0.04339100
H	4.54039600	-0.44871900	0.91629800
H	5.05078800	-2.80198900	2.19736400
H	3.65887400	-1.83842100	2.71126400
C	-2.34662700	2.17828500	-1.68018300
C	-3.37073800	3.34216500	-1.78447100
H	-2.17454600	1.73613100	-2.66878700
C	-4.29884100	1.64877800	-0.37471200
C	-4.24177500	3.17044200	-0.52972200
H	-3.97945100	3.20661900	-2.68393700
H	-2.88481100	4.31733400	-1.85568000
H	-5.05765100	1.22832500	-1.05269200
H	-4.54556700	1.33189900	0.64365900
H	-5.23209300	3.62041000	-0.63473100
H	-3.75518500	3.62123900	0.34287300
N	-2.93692300	1.21019900	-0.73951700
N	2.91992300	-1.48656400	0.03046800
C	2.76802500	-1.01136600	-1.27195300
H	1.79568000	-1.23893100	-1.70484600
C	3.66362000	-0.30901800	-1.98737300
H	4.63561700	-0.06559300	-1.56137600
C	3.40808500	0.19042300	-3.38913200
H	2.38424800	-0.09237800	-3.66978500
C	4.37256900	-0.46530800	-4.39683700
H	5.41501700	-0.23254200	-4.14496200
H	4.26296500	-1.55491400	-4.39726300
H	4.18533000	-0.10105700	-5.41402400
C	3.51531300	1.72523100	-3.46804000
H	3.32531000	2.08009100	-4.48792600
H	2.79653800	2.20772600	-2.79825900
H	4.52031400	2.05911300	-3.18015600
H	-2.92802200	0.26258600	-1.11534700
C	-4.77534400	-3.49415400	-2.37961300
H(Iso=2)	-5.22989300	-4.37273700	-1.91519800
H(Iso=2)	-4.24212700	-3.76041000	-3.29513400
H(Iso=2)	-5.53841800	-2.74030800	-2.58055000
S	-3.60413400	-2.75455700	-1.20155100
C	-2.42102500	-4.13191100	-1.12445000
H(Iso=2)	-1.61547500	-3.82206500	-0.45534800
H(Iso=2)	-2.93349800	-5.00586300	-0.71429500
H(Iso=2)	-2.04426600	-4.33117100	-2.13057400
O	-2.89829700	-1.62623000	-1.95673900

R_a-2_c1

C	-0.30074600	-1.76742500	0.37584100
C	-0.83007900	-1.44952000	-0.89747100
C	-0.41311300	-2.17368700	-2.02475200
C	0.51637900	-3.20374900	-1.89000500
C	1.04253600	-3.52882600	-0.64102200
C	0.62909700	-2.80737100	0.47867600
C	-0.72813500	-1.03336100	1.61012700
C	-1.83100700	-1.49113600	2.33928600
C	-2.24720700	-0.85470100	3.50863300
C	-1.54699500	0.26385200	3.95753200
C	-0.44389500	0.74338000	3.25329600
C	-0.02291100	0.10226500	2.07716800
H	-0.82178600	-1.92348300	-2.99223800
H	0.82578500	-3.75367100	-2.77313300
H	1.76420600	-4.33172800	-0.53565500
H	1.03105500	-3.04812200	1.45795600
H	-2.36565600	-2.36381200	1.97726500
H	-3.10396100	-1.22979800	4.05800800
H	-1.85514600	0.77282500	4.86541600
H	0.10037800	1.60822600	3.60239500
N	1.08717000	0.53888400	1.33712400
H	1.37261800	-0.00548600	0.52035400
N	-1.77282200	-0.40741600	-0.97394500
H	-2.07875900	-0.00414500	-0.09254000
C	1.91917600	1.59478600	1.53912800
C	-2.38917200	0.13922800	-2.05513000
O	-2.21158200	-0.20139400	-3.22299600
O	1.84281000	2.40151900	2.46643500
C	-3.34518400	1.30158400	-1.73419700
C	-2.69455700	2.67650400	-2.05662900
H	-4.22115800	1.13759600	-2.37471200
C	-3.47416200	2.74493900	0.20515700

C	-2.33807200	3.25745600	-0.67812200
H	-3.43555700	3.31040900	-2.55234100
H	-1.83797800	2.58188200	-2.72686700
H	-4.36564900	3.38296100	0.10243200
H	-3.21195300	2.69225800	1.26576700
H	-2.27630000	4.34789600	-0.68517600
H	-1.37809500	2.86456900	-0.32701100
C	3.01338300	1.72843900	0.47386500
C	2.85327800	3.04822700	-0.33465200
H	3.95926800	1.74734900	1.02829200
C	2.91473300	1.22059700	-1.87019400
C	3.38645700	2.67435400	-1.72495200
H	3.38593000	3.87831300	0.13294300
H	1.79477100	3.32532900	-0.39896300
H	3.50644100	0.63439400	-2.57837800
H	1.87091700	1.19569300	-2.20987600
H	4.48197400	2.71851900	-1.73806900
H	3.00849800	3.33031100	-2.51474000
N	3.01720700	0.63295000	-0.51571900
N	-3.72697000	1.39352400	-0.32639900
C	-4.82493500	0.68357600	0.15367900
H	-5.13080900	1.00288500	1.14818700
C	-5.47665200	-0.32899900	-0.44489600
H	-5.17416500	-0.67490100	-1.43231800
C	-6.63301800	-1.06625900	0.18675700
H	-6.81967300	-0.62246900	1.17426500
C	-6.30201800	-2.55730100	0.39205500
H	-6.08858200	-3.04374700	-0.56815500
H	-5.42404400	-2.68322800	1.03418200
H	-7.14364700	-3.08638400	0.85460100
C	-7.91698100	-0.91301100	-0.65135700
H	-8.75404800	-1.44967800	-0.18927700
H	-8.19943300	0.14015400	-0.75298100
H	-7.77393500	-1.32148500	-1.65976800
H	3.87706600	0.09278200	-0.42880000
C	7.80068900	-1.03286300	1.29501700
H(Iso=2)	8.84244000	-0.70458400	1.26291100
H(Iso=2)	7.72837600	-2.12221400	1.33351900
H(Iso=2)	7.29168000	-0.58022500	2.14759600
S	6.95403300	-0.43408400	-0.19863600
C	7.90930800	-1.38055000	-1.42251800
H(Iso=2)	7.46885800	-1.16707800	-2.39784500
H(Iso=2)	8.94668300	-1.03836000	-1.39694900
H(Iso=2)	7.83606200	-2.44502000	-1.18826100
O	5.55796300	-1.05857000	-0.17383900

R_a-endo-3

O	0.74810900	-1.11781600	-1.59349500
O	-2.78406000	-3.18220500	1.77389800
N	4.14856400	-0.04328900	-1.53146700
N	2.08535100	0.48817200	-0.59953100
C	6.14978800	2.75628800	1.35075300
H	6.32689500	1.87041300	1.97092200
H	5.53332600	3.45448800	1.93127900
H	7.11621400	3.23825200	1.16699800
C	5.46081700	2.38675000	0.02875200
H	6.11420500	1.68517300	-0.50558700
C	5.28604300	3.63329800	-0.85257700
H	6.25198900	4.12252200	-1.01913400
H	4.62191500	4.36185600	-0.37040900
H	4.86726900	3.39481900	-1.83531300
C	4.12301200	1.67551900	0.32394400
H	4.27990400	0.89696700	1.07731500
H	3.43341200	2.40253100	0.76931500
C	3.44163400	1.06603100	-0.89666600
H	3.30228100	1.84239800	-1.65974300
C	4.81560200	-1.02630600	-0.64628700
H	4.21688600	-1.25999800	0.24783700
H	5.78819200	-0.65114500	-0.31994700
C	4.91749600	-2.26269000	-1.54020200
H	5.09636500	-3.17467500	-0.96572000
H	5.74172500	-2.14085800	-2.25026600
C	3.56343900	-2.28031900	-2.28383700
H	3.65486600	-2.65268900	-3.30584200
H	2.83333200	-2.91110400	-1.76897300
C	3.10460900	-0.80653500	-2.25050200

H	2.94839800	-0.39500000	-3.25577100
C	1.82064900	-0.53996200	-1.45834700
C	1.05837900	1.17258300	0.11197700
C	0.72910200	2.48042100	-0.26656300
H	1.26209800	2.94712300	-1.08770500
C	-0.66634600	1.25761500	1.79467800
H	-1.20906900	0.77912400	2.60365700
C	0.36383300	0.54397700	1.16266500
C	0.71317600	-0.81807200	1.67152600
C	1.89265600	-0.99663000	2.40479900
H	2.55026000	-0.14571600	2.54814000
C	1.35486300	-3.30910200	2.80244300
H	1.59485900	-4.27521000	3.23495400
C	0.18048200	-3.15956700	2.06900700
H	-0.48672600	-3.99870200	1.92679000
C	-0.14932300	-1.92139700	1.50053800
C	-2.51050300	-2.38458500	0.87407100
C	-3.55220900	-2.02730300	-0.18966200
H	-4.45073200	-1.73806200	0.36766700
C	-3.86855800	-3.25880200	-1.08909700
H	-4.67817300	-3.86435100	-0.67739600
H	-2.98365400	-3.90000600	-1.17604800
C	-4.19632000	-2.62651500	-2.44910600
H	-4.06389300	-3.32222700	-3.28310900
H	-5.23231400	-2.26753800	-2.46344700
C	-3.22446400	-1.43876700	-2.49271800
H	-2.23470600	-1.77454600	-2.82857500
H	-3.54402300	-0.63309600	-3.15934600
C	-0.27997300	3.17831400	0.39095800
H	-0.51952100	4.19236000	0.08861100
C	-0.98674600	2.56105400	1.42370700
H	-1.77928100	3.09178400	1.94051400
C	2.21937000	-2.22704100	2.97172400
H	3.13497400	-2.33584900	3.54351300
N	-1.31297400	-1.76783000	0.71924100
H	-1.29713300	-1.09665600	-0.04783400
N	-3.14101100	-0.94105300	-1.10130700
H	-3.76920900	-0.14814900	-0.98264700
C	-6.23578700	3.77270700	-0.84854400
H(Iso=2)	-6.33232200	4.65205400	-0.20717900
H(Iso=2)	-7.21317500	3.35068100	-1.09379200
H(Iso=2)	-5.68570400	4.02921400	-1.75541800
S	-5.24934400	2.52058600	0.02638300
C	-6.40134200	2.21642000	1.40050200
H(Iso=2)	-5.96380700	1.42262100	2.00818300
H(Iso=2)	-6.49239100	3.13236700	1.98946100
H(Iso=2)	-7.36738200	1.90687200	0.99514600
O	-5.28610400	1.26580500	-0.84657800

S_a-endo-3

O	0.19209100	1.25487600	-0.40997700
O	3.29136100	-2.45372300	-2.26264700
N	-3.30913900	1.76993100	-0.79920800
N	-1.81846100	0.37448900	0.31654800
C	-6.64753800	0.27504100	1.80445900
H	-6.44816200	1.13624000	2.45206900
H	-6.49216100	-0.63455700	2.39862200
H	-7.70358400	0.30787000	1.51497700
C	-5.73885800	0.28228800	0.56624300
H	-5.93170000	1.21100800	0.01425100
C	-6.08198300	-0.89498100	-0.35955800
H	-7.14459000	-0.87501500	-0.62525500
H	-5.88002400	-1.85328000	0.13568400
H	-5.51119600	-0.87152800	-1.29308200
C	-4.26044400	0.27947700	1.01102400
H	-4.10736900	1.04572900	1.77844800
H	-4.04326000	-0.68331700	1.48923300
C	-3.25649700	0.47954900	-0.11842700
H	-3.40867000	-0.29660100	-0.87752100
C	-3.51182300	2.97188100	0.04125300
H	-2.94634100	2.92167200	0.98620300
H	-4.56925000	3.09894800	0.28316200
C	-2.95583800	4.09288400	-0.83828700
H	-2.72746500	4.99367400	-0.26372000
H	-3.68756600	4.35786900	-1.60802300
C	-1.70111600	3.45902100	-1.48223900

H	-1.55577100	3.78684400	-2.51329500
H	-0.79309400	3.70980100	-0.92704300
C	-1.96571100	1.94193100	-1.39781300
H	-1.91414700	1.44972500	-2.37706300
C	-1.03164000	1.17099400	-0.45536900
C	-1.29429500	-0.58165800	1.23849600
C	-1.30462200	-0.26589900	2.60118100
H	-1.71341100	0.68907300	2.91256800
C	-0.23317700	-2.68403200	1.76214700
H	0.18453800	-3.63052100	1.43375900
C	-0.75204100	-1.80528200	0.79888500
C	-0.74267200	-2.21212200	-0.64115700
C	-1.93003300	-2.64924300	-1.23832200
H	-2.83663800	-2.66501700	-0.64218200
C	-0.78881800	-3.08935100	-3.30740800
H	-0.79547200	-3.42387300	-4.34000100
C	0.41024400	-2.66511800	-2.73829500
H	1.32407100	-2.66991900	-3.31362300
C	0.44890400	-2.22799300	-1.40458600
C	2.92539900	-1.91555200	-1.21594700
C	3.95482800	-1.32535500	-0.24496300
H	4.52198800	-0.59291100	-0.83179800
C	4.91723600	-2.42625400	0.28949900
H	5.79549600	-2.54269900	-0.34810500
H	4.40188100	-3.39281500	0.32521800
C	5.24444100	-1.94876500	1.71163900
H	5.57426200	-2.76108100	2.36620000
H	6.03266200	-1.18689600	1.68738400
C	3.91568400	-1.31883400	2.15265300
H	3.22996100	-2.09801800	2.51093900
H	4.02148500	-0.57922600	2.95076900
C	-0.79259500	-1.15538700	3.54250800
H	-0.80954500	-0.89715400	4.59604700
C	-0.25457000	-2.37111100	3.11978900
H	0.14656700	-3.07416400	3.84231500
C	-1.96627700	-3.08713700	-2.56176500
H	-2.90004800	-3.42584400	-2.99772300
N	1.63903000	-1.78893600	-0.79993800
H	1.57884100	-1.28753800	0.08737900
N	3.35845900	-0.67946500	0.94062500
H	3.57755600	0.31481700	0.93808100
C	5.27671600	4.29699700	-0.33893200
H(Iso=2)	5.02634300	5.13159200	-0.99834600
H(Iso=2)	5.86713400	3.54102200	-0.86186900
H(Iso=2)	5.80773400	4.66277200	0.54154200
S	3.73281500	3.54103100	0.25555600
C	3.11435300	2.93579200	-1.34263600
H(Iso=2)	2.18441100	2.40034800	-1.13851800
H(Iso=2)	2.92127400	3.79818700	-1.98607500
H(Iso=2)	3.86301400	2.27435100	-1.78534600
O	4.14935200	2.30523800	1.05564900

R_a-exo-3

O	-1.03254600	1.39450700	-2.27711700
O	1.77429400	2.82102400	2.02200100
N	-3.96431600	1.00740100	-0.26904600
N	-2.01590200	-0.12963300	-0.83801600
C	-5.42727800	-2.48604900	0.90244600
H	-4.87802000	-1.93185600	1.67041600
H	-4.90608500	-3.43810100	0.73921200
H	-6.41800500	-2.71485500	1.31072900
C	-5.55245900	-1.70041900	-0.41240300
H	-6.06537900	-0.75339800	-0.19950500
C	-6.40418900	-2.48152900	-1.42398900
H	-7.39269500	-2.71133400	-1.01060500
H	-5.92471000	-3.43271500	-1.68901800
H	-6.54925600	-1.91104200	-2.34833100
C	-4.18216900	-1.35824300	-1.02702700
H	-3.59080000	-2.27828300	-1.12246400
H	-4.32489300	-0.96759800	-2.04272800
C	-3.36909900	-0.32588800	-0.23444700
H	-3.24926900	-0.64774100	0.80176500
C	-3.83751200	1.78718800	0.98048400
H	-2.86769600	1.61312200	1.47511900
H	-4.63133500	1.50977800	1.67990200
C	-3.93090700	3.23371000	0.49753500

H	-3.54866300	3.94534700	1.23326900
H	-4.97393900	3.49050500	0.28414500
C	-3.10192500	3.21724900	-0.80256900
H	-3.44015800	3.95711300	-1.53038800
H	-2.04744500	3.42008800	-0.59266000
C	-3.26955600	1.77173400	-1.32929300
H	-3.83473600	1.75057300	-2.26884500
C	-1.95932000	1.02270000	-1.56451200
C	-1.02392500	-1.15431500	-0.90302300
C	-0.78798200	-1.79132000	-2.12642500
H	-1.35850600	-1.48535400	-2.99598000
C	0.64095900	-2.56841000	0.12301000
H	1.19997100	-2.86891500	1.00349300
C	-0.29705100	-1.53220100	0.24280400
C	-0.50299100	-0.88282800	1.57628500
C	-1.34491300	-1.49268000	2.51199200
H	-1.83965600	-2.41933800	2.23808100
C	-0.92291600	0.25654700	4.10445700
H	-1.08282000	0.71047700	5.07739700
C	-0.06191800	0.87605300	3.20050300
H	0.44623700	1.79181300	3.46314200
C	0.16848900	0.30886000	1.93645000
C	1.83134800	1.99328800	1.11149000
C	2.80689600	2.18569300	-0.05435300
H	3.78603200	2.33817200	0.41720300
C	2.44678200	3.43315300	-0.90614400
H	2.88008100	4.34607200	-0.49332500
H	1.35921700	3.56395000	-0.94266800
C	2.98184000	3.06448100	-2.29689100
H	2.50998000	3.63924700	-3.09937000
H	4.06408700	3.23500100	-2.34446600
C	2.67396000	1.56162800	-2.37280500
H	1.63763600	1.40562500	-2.69351100
H	3.32637700	1.01755400	-3.06159700
C	0.15874300	-2.80740300	-2.22794100
H	0.32983400	-3.29199400	-3.18344100
C	0.87255200	-3.20260100	-1.09642600
H	1.60658600	-3.99896500	-1.16081300
C	-1.56239200	-0.93629400	3.77227500
H	-2.22320700	-1.42757900	4.47839200
N	1.08195700	0.86217100	1.02298400
H	1.31949300	0.32688900	0.18578700
N	2.85294700	1.04807900	-0.99304500
H	3.74806300	0.56872400	-0.90788000
C	6.69377300	-2.75516800	-0.65983900
H(Iso=2)	6.93974600	-3.55492300	0.04299500
H(Iso=2)	7.59469200	-2.32344600	-1.10182700
H(Iso=2)	6.02342600	-3.13061900	-1.43471800
S	5.78778000	-1.45520900	0.23199400
C	7.12123100	-0.95588100	1.36288900
H(Iso=2)	6.74035200	-0.11615000	1.94654300
H(Iso=2)	7.35323100	-1.79593400	2.02205700
H(Iso=2)	7.99388600	-0.65762200	0.77726000
O	5.61483300	-0.30195500	-0.75763400

S_a-exo-3

O	-0.14953100	0.90636600	0.39015400
O	-3.19475600	-3.15882800	1.75051000
N	3.11699400	2.26275300	-0.03087400
N	1.90860900	0.31790900	-0.47820000
C	6.30795400	0.29830100	-1.35855100
H	5.72725100	0.80493100	-2.13649200
H	6.30658200	-0.77538800	-1.58602200
H	7.34099200	0.65435500	-1.43916500
C	5.75505600	0.55692300	0.05200900
H	5.76777300	1.63966300	0.23410600
C	6.64976200	-0.10877300	1.10800400
H	7.68466600	0.24134700	1.02320200
H	6.65635200	-1.19954700	0.98540400
H	6.30069000	0.11191400	2.12298800
C	4.30009100	0.07596100	0.21106700
H	4.23019700	-0.96864200	-0.12032100
H	4.01910600	0.09714200	1.27067300
C	3.27383100	0.90869500	-0.56481300
H	3.56547100	0.96877600	-1.61717000
C	3.04429400	3.33230100	-1.04736400

H	2.47999900	3.01051900	-1.93999700
H	4.05000400	3.61863400	-1.36801500
C	2.29290400	4.44943000	-0.32535500
H	1.87856400	5.18766800	-1.01605600
H	2.96667800	4.96803100	0.36479700
C	1.20463500	3.68378300	0.45413400
H	0.91177800	4.18782600	1.37696400
H	0.30364300	3.56022100	-0.15395900
C	1.84709400	2.30488500	0.73045000
H	2.02391600	2.15128400	1.80210600
C	1.04737900	1.11097400	0.21731600
C	1.52250000	-0.87238600	-1.16078000
C	1.71965900	-0.92972700	-2.54706600
H	2.15647200	-0.07721700	-3.05544900
C	0.54729100	-3.07543200	-1.24713300
H	0.09345600	-3.91798000	-0.73536200
C	0.93710300	-1.96186600	-0.48540700
C	0.76400500	-2.03261000	0.99927400
C	1.88357700	-2.22714800	1.81744800
H	2.86519800	-2.26413100	1.35904100
C	0.49044100	-2.38914500	3.77553700
H	0.37560100	-2.52544300	4.84614600
C	-0.63752900	-2.18862200	2.98339800
H	-1.62287100	-2.16316500	3.42951500
C	-0.51195800	-2.00832900	1.60019800
C	-2.87925000	-2.30501400	0.91809000
C	-3.91505800	-1.78535200	-0.09353200
H	-4.76098600	-1.45650600	0.52269000
C	-4.37249000	-2.90619100	-1.06881700
H	-5.45733400	-2.84826500	-1.20218100
H	-4.13729000	-3.90421500	-0.69340600
C	-3.66156800	-2.55422000	-2.38549500
H	-2.63233400	-2.93018300	-2.38094400
H	-4.16794800	-2.96204200	-3.26407400
C	-3.66239400	-1.02397600	-2.36137200
H	-2.89160800	-0.58662000	-3.00455400
H	-4.63881400	-0.64135600	-2.69820800
C	1.34704200	-2.05392900	-3.27674000
H	1.51027500	-2.07961900	-4.34895800
C	0.75081500	-3.13293700	-2.62298900
H	0.45091000	-4.01527600	-3.17849900
C	1.75857800	-2.40496900	3.19455100
H	2.64276800	-2.56265100	3.80327900
N	-1.64720300	-1.75622200	0.80031200
H	-1.57181100	-1.06839400	0.05079900
N	-3.40414100	-0.69845700	-0.94518200
H	-3.80684300	0.20021300	-0.68384400
C	-4.89500700	4.59688000	-0.49152400
H(iso=2)	-4.40163300	5.51283300	-0.15719900
H(iso=2)	-5.93667200	4.56454500	-0.16387100
H(iso=2)	-4.82721000	4.50990000	-1.57721700
S	-4.00543200	3.17232300	0.20541400
C	-4.34527500	3.50474200	1.96070400
H(iso=2)	-3.89994000	2.68588700	2.52811800
H(iso=2)	-3.87298000	4.45128600	2.23456500
H(iso=2)	-5.42620600	3.54091600	2.11474900
O	-4.82691900	1.93574100	-0.16174200

R_a-4_c1

C	0.10554600	-1.59340700	-0.74164300
C	-0.62095100	-0.72644000	-1.59251400
C	-0.40478900	-0.77023300	-2.97814600
C	0.51827400	-1.66688700	-3.51415000
C	1.23572900	-2.52961600	-2.68744600
C	1.02249300	-2.48421200	-1.30979000
C	-0.10555000	-1.59342200	0.74160200
C	-1.02249700	-2.48423800	1.30973100
C	-1.23573300	-2.52967000	2.68738700
C	-0.51827800	-1.66695700	3.51410700
C	0.40478500	-0.77029200	2.97812200
C	0.62094700	-0.72647200	1.59249000
H	-0.96326400	-0.10545100	-3.61986700
H	0.67014000	-1.68793600	-4.58862200
H	1.95174900	-3.22925800	-3.10494700
H	1.57509800	-3.14858000	-0.65290400
H	-1.57510100	-3.14859400	0.65283200

H	-1.95175200	-3.22932100	3.10487400
H	-0.67014400	-1.68802800	4.58857900
H	0.96325900	-0.10552200	3.61985600
N	1.55527200	0.14251400	0.99943600
H	1.73948200	0.00762500	0.00895500
N	-1.55527600	0.14253300	-0.99944300
H	-1.73948100	0.00763000	-0.00896200
C	2.30598900	1.13127800	1.55409800
C	-2.30599700	1.13130500	-1.55408600
O	-2.28070000	1.46402900	-2.73704200
O	2.28068600	1.46398300	2.73706000
C	-3.21194900	1.88584700	-0.56541100
C	-2.61050000	3.26823400	-0.18419200
H	-4.16379200	2.02051600	-1.09507200
C	-3.09701100	2.09310100	1.84336500
C	-2.08500600	3.06969900	1.24742200
H	-3.40945700	4.01534800	-0.19185800
H	-1.84467600	3.59498300	-0.89032900
H	-3.99793000	2.62580100	2.18524500
H	-2.70226200	1.51512800	2.68385700
H	-2.03019300	4.00545900	1.80800000
H	-1.08633800	2.62069200	1.23682100
C	3.21194500	1.88583600	0.56544000
C	2.61049500	3.26822600	0.18423500
H	4.16378400	2.02050100	1.09510900
C	3.09702200	2.09312200	-1.84333500
C	2.08501000	3.06970900	-1.24738400
H	3.40945000	4.01534200	0.19191600
H	1.84466500	3.59496400	0.89037300
H	3.99794200	2.62582900	-2.18520200
H	2.70228000	1.51515800	-2.68383600
H	2.03019700	4.00547600	-1.80795100
H	1.08634400	2.62069800	-1.23679600
N	3.40733100	1.20150800	-0.71122200
N	-3.40732400	1.20150100	0.71124300
C	4.43065100	0.26807100	-0.86041100
H	4.60663800	-0.00150800	-1.90011700
C	5.14966500	-0.32717400	0.10752300
H	4.97619100	-0.08402200	1.15487700
C	6.20978600	-1.36820200	-0.16062300
H	6.27444500	-1.51475300	-1.24748500
C	5.84062600	-2.72097500	0.47876200
H	5.74429700	-2.62292600	1.56747700
H	4.88778700	-3.09530000	0.09015400
H	6.61210400	-3.47383600	0.27814200
C	7.59052300	-0.89899600	0.33694300
H	7.57209500	-0.71560900	1.41871400
H	8.35752600	-1.65727400	0.13977400
H	7.89332300	0.03091000	-0.15586300
C	-4.43064300	0.26806100	0.86042600
H	-4.60662300	-0.00153100	1.90013000
C	-5.14966200	-0.32717200	-0.10751100
H	-4.97619400	-0.08400700	-1.15486300
C	-6.20977900	-1.36820600	0.16062900
H	-6.27443400	-1.51476800	1.24748900
C	-5.84061700	-2.72097200	-0.47877000
H	-5.74429200	-2.62291200	-1.56748500
H	-4.88777600	-3.09529800	-0.09016900
H	-6.61209300	-3.47383600	-0.27815600
C	-7.59051900	-0.89899900	-0.33692900
H	-8.35751900	-1.65728100	-0.13976400
H	-7.89332000	0.03090100	0.15588600
H	-7.57209500	-0.71560200	-1.41869800

S_a-4_c1

C	0.37175400	-0.64994200	-2.38941500
C	-0.09632100	-1.77941600	-1.67607600
C	0.61008300	-2.98990100	-1.75106500
C	1.76888500	-3.07943400	-2.51978200
C	2.24440800	-1.97282700	-3.22122500
C	1.54199300	-0.77084200	-3.14846100
C	-0.37176400	0.64992800	-2.38941800
C	-1.54200500	0.77082400	-3.14846000
C	-2.24441900	1.97280900	-3.22122900
C	-1.76889300	3.07942000	-2.51979600
C	-0.61008900	2.98989100	-1.75108100

C	0.09631400	1.77940600	-1.67608700
H	0.24438100	-3.84654700	-1.20540800
H	2.30013700	-4.02477500	-2.56498300
H	3.14472200	-2.04208000	-3.82213200
H	1.89450900	0.09612700	-3.69864000
H	-1.89452400	-0.09614900	-3.69863300
H	-3.14473600	2.04205800	-3.82213300
H	-2.30014600	4.02476100	-2.56500100
H	-0.24438500	3.84654100	-1.20543200
N	1.26376400	1.63660400	-0.90260000
H	1.66757100	0.70569700	-0.84793200
N	-1.26376800	-1.63661000	-0.90258500
H	-1.66757200	-0.70570100	-0.84791800
C	1.96701600	2.56595300	-0.20284700
C	-1.96702200	-2.56595600	-0.20283200
O	-1.68185200	-3.75867900	-0.11736200
O	1.68184400	3.75867500	-0.11737800
C	-3.22550600	-2.03378000	0.50816600
C	-4.51702700	-2.52849900	-0.19245800
H	-3.16626700	-2.43417200	1.52616400
C	-4.58212400	-0.13419100	-0.15220000
C	-4.95727400	-1.32360500	-1.03841600
H	-5.27109200	-2.74901000	0.56913100
H	-4.34951800	-3.43821100	-0.77185200
H	-5.36874100	0.06908200	0.59230600
H	-4.41055800	0.78749000	-0.71709100
H	-6.02312300	-1.34124900	-1.27625200
H	-4.40119300	-1.28736700	-1.98136000
C	3.22550600	2.03378100	0.50814500
C	4.51702200	2.52849900	-0.19249000
H	3.16627300	2.43417800	1.52614100
C	4.58212600	0.13419200	-0.15221300
C	4.95727000	1.32360000	-1.03844000
H	5.27109000	2.74902000	0.56909300
H	4.34950700	3.43820600	-0.77189100
H	5.36874600	-0.06906900	0.59229300
H	4.41056500	-0.78749500	-0.71709500
H	6.02311800	1.34124500	-1.27628100
H	4.40118500	1.28735200	-1.98138200
N	3.34911600	0.56979600	0.51477700
N	-3.34911500	-0.56979500	0.51479300
C	2.86720300	-0.14107200	1.61189400
H	2.07206900	0.37518100	2.14667300
C	3.25944200	-1.35945500	2.02365000
H	4.04551900	-1.89130500	1.48994300
C	2.65400700	-2.07647600	3.20627800
H	1.87553600	-1.42960700	3.63368300
C	3.70790200	-2.33446800	4.30054700
H	4.52330200	-2.95951200	3.91487100
H	4.14491200	-1.39593200	4.65756700
H	3.26510800	-2.85494000	5.15806300
C	1.98926800	-3.39985600	2.78057100
H	1.19864200	-3.22676300	2.04325900
H	2.72596400	-4.07707300	2.33014400
H	1.54760100	-3.91312300	3.64301900
C	-2.86719900	0.14107800	1.61190500
H	-2.07206900	-0.37517500	2.14668900
C	-3.25943000	1.35946800	2.02365000
H	-4.04550200	1.89131900	1.48993700
C	-2.65399300	2.07649500	3.20627400
H	-1.87552400	1.42962500	3.63368300
C	-3.70788600	2.33449800	4.30054100
H	-4.52328400	2.95954200	3.91486000
H	-4.14490000	1.39596600	4.65756700
H	-3.26509100	2.85497400	5.15805300
C	-1.98924800	3.39986900	2.78055800
H	-1.54758000	3.91314000	3.64300300
H	-1.19862200	3.22676800	2.04324700
H	-2.72594100	4.07708600	2.33012600

S_a-endo-5

O	0.37583300	1.10096600	0.03340700
N	-2.97837800	2.18416100	-0.50111800
N	-1.79504700	0.39580500	0.39946800
C	-6.75351800	0.61501000	1.36069200
H	-6.54490100	1.28368400	2.20337600

H	-6.76848800	-0.41326300	1.74401300
H	-7.75689500	0.84623100	0.98676900
C	-5.70506900	0.76970300	0.24904400
H	-5.72937500	1.81250200	-0.09211200
C	-6.05577300	-0.12609200	-0.94904500
H	-7.06942100	0.09013100	-1.30380700
H	-6.01996400	-1.18630300	-0.66789000
H	-5.37562800	0.02148200	-1.79370800
C	-4.30076400	0.47787400	0.82038100
H	-4.16215300	1.03414000	1.75350500
H	-4.24702200	-0.58545000	1.08316200
C	-3.15126100	0.78273000	-0.13265400
H	-3.29136100	0.21246700	-1.05902300
C	-3.15568700	3.19287400	0.56818000
H	-2.72555600	2.86475000	1.52854100
H	-4.21545400	3.40593200	0.72406900
C	-2.37237500	4.39106600	0.03046600
H	-2.12794900	5.11097900	0.81509200
H	-2.96256000	4.90775600	-0.73302100
C	-1.11807800	3.74652100	-0.60186700
H	-0.78979100	4.27232200	-1.50027900
H	-0.27746800	3.73505600	0.09738300
C	-1.56120600	2.30201600	-0.91487900
H	-1.44616700	2.04734900	-1.97593700
C	-0.84037900	1.21422900	-0.11132100
C	-1.49141300	-0.81189000	1.09783700
C	-1.67119000	-0.84754100	2.48456700
H	-2.03703000	0.04024600	2.98841700
C	-0.69845400	-3.08798100	1.16341200
H	-0.31698700	-3.96268200	0.64649200
C	-0.99645100	-1.93882700	0.41739200
C	-0.80827400	-1.96374800	-1.06632200
C	-1.90474100	-2.21375800	-1.90365300
H	-2.88495400	-2.34946900	-1.45818400
C	-0.48601600	-2.16693600	-3.85354700
H	-0.35399200	-2.24333600	-4.92781100
C	0.61263500	-1.90650000	-3.03856700
H	1.59781900	-1.77309800	-3.46995700
C	0.46033400	-1.80546100	-1.65211800
C	-1.37810100	-2.00070400	3.20819100
H	-1.52486500	-2.01666400	4.28293200
C	-0.88982000	-3.12613200	2.54327400
H	-0.65714400	-4.03064800	3.09556600
C	-1.75175500	-2.31755300	-3.28513400
H	-2.61473900	-2.51874000	-3.91116400
N	1.59047300	-1.50885300	-0.83921400
H	1.54267600	-0.67363800	-0.25770400
C	2.71565800	-2.25945700	-0.78863000
O	2.87738400	-3.30450300	-1.42107200
C	3.83027500	-1.76455600	0.14821700
C	3.88667800	-2.60080700	1.45336300
H	4.75525300	-1.89465500	-0.42487300
C	3.18732800	-1.70977000	2.49250600
H	4.93402700	-2.75284800	1.73150700
H	3.42623600	-3.58359100	1.33519700
C	3.60717700	-0.30003900	2.07001400
H	2.09999900	-1.81936300	2.42709200
H	3.48871000	-1.94105600	3.51672800
H	2.89454900	0.47162200	2.37976000
H	4.59100300	-0.03342700	2.49069900
N	3.67935100	-0.38190800	0.60967200
C	4.25698400	0.64662500	-0.12173300
H	4.42180300	0.39458200	-1.16794800
C	4.58581100	1.87427800	0.32129100
H	4.41461900	2.14461200	1.36220300
C	5.17431800	2.94986200	-0.55977200
H	5.29439200	2.53327700	-1.56945600
C	4.23793100	4.16995100	-0.66123400
H	4.06682200	4.61169700	0.32870600
H	4.67101300	4.94656600	-1.30308500
H	3.26429600	3.88743700	-1.07489600
C	6.56551300	3.38428800	-0.05925800
H	7.26140800	2.53879200	-0.04243500
H	6.98961900	4.16483900	-0.70218900
H	6.50319900	3.78799500	0.95936400

Ra-endo-5

O	-0.17865900	-0.25471800	-1.50546500
N	3.32627700	0.26116400	-1.91276300
N	1.64516100	0.55564000	-0.33332100
C	6.39956900	1.17214600	1.23800200
H	6.47950900	0.08754500	1.37214900
H	6.07052100	1.60491500	2.19139500
H	7.40118700	1.56047600	1.02352200
C	5.42039700	1.52689400	0.10917100
H	5.79298900	1.06693100	-0.81506500
C	5.37625600	3.04774200	-0.10557500
H	6.38227400	3.43584900	-0.29896800
H	4.98810600	3.55660100	0.78590800
H	4.74802000	3.33164500	-0.95574000
C	4.03193100	0.93018200	0.42317800
H	4.14016500	-0.11899100	0.71611700
H	3.61706000	1.45342400	1.29310300
C	3.02058500	1.03462600	-0.71292200
H	2.92241300	2.08471600	-1.01612200
C	3.90671800	-1.08936100	-1.72801100
H	3.44918300	-1.63141700	-0.88565800
H	4.98230900	-1.02352200	-1.54963900
C	3.55711000	-1.78391600	-3.04456100
H	3.62904300	-2.87155400	-2.97017600
H	4.23977700	-1.45084700	-3.83283600
C	2.11972600	-1.29842800	-3.33592900
H	1.93114200	-1.17259500	-4.40362800
H	1.37530400	-2.00139100	-2.95154100
C	2.02167100	0.04169600	-2.57566400
H	1.77792500	0.88162500	-3.23855400
C	1.00356600	0.07005600	-1.43224900
C	0.95103200	0.96117400	0.84424400
C	0.81187700	2.32896800	1.10984400
H	1.23495800	3.05088000	0.41982000
C	-0.30984300	0.47188900	2.83920200
H	-0.74419300	-0.25617700	3.51686900
C	0.39631000	0.00998600	1.71839500
C	0.58139300	-1.46445800	1.54496400
C	1.78789200	-2.05549600	1.94534100
H	2.58244500	-1.41866600	2.32012800
C	0.93641100	-4.25238700	1.43661500
H	1.06530200	-5.32893700	1.39218600
C	-0.26407400	-3.68361900	1.02082800
H	-1.06629200	-4.30809400	0.64735900
C	-0.44841900	-2.29682700	1.07082800
C	0.12870600	2.76602000	2.24126100
H	0.03364700	3.82882500	2.43694200
C	-0.44211500	1.83243400	3.10705100
H	-0.98344800	2.16030500	3.98828600
C	1.97110300	-3.43569300	1.89638700
H	2.91154300	-3.86926000	2.22016300
N	-1.65796900	-1.72384000	0.59721400
H	-1.57359100	-0.95497300	-0.06590000
C	-2.90399500	-2.12874100	0.93654500
O	-3.14715400	-3.01064000	1.76207300
C	-4.06956100	-1.43594200	0.20917200
C	-4.72235400	-2.39692600	-0.81691000
H	-4.78389400	-1.17254900	0.99658300
C	-4.07904100	-1.98865300	-2.15115500
H	-5.79991400	-2.20685900	-0.84476200
H	-4.57220500	-3.44586300	-0.55523400
C	-3.95465200	-0.46903900	-2.01448300
H	-3.08937500	-2.44515300	-2.25918600
H	-4.67954800	-2.27714700	-3.01678600
H	-3.14568100	-0.04727500	-2.61983600
H	-4.89068900	0.03298500	-2.30929200
N	-3.69601400	-0.26231700	-0.58639500
C	-3.81373000	1.00706600	-0.03710100
H	-3.78705900	1.01431400	1.05103400
C	-3.92491400	2.17127500	-0.70405700
H	-3.93929600	2.17869900	-1.79283300
C	-4.02327000	3.51764400	-0.02795500
H	-3.96054600	3.35856600	1.05741400
C	-2.85985000	4.44013900	-0.44016900
H	-2.86864700	4.61418200	-1.52372200
H	-2.93584400	5.41570800	0.05490700

H	-1.89313200	3.99778900	-0.17888900
C	-5.37421400	4.19662900	-0.32777300
H	-6.21113900	3.58082100	0.01816700
H	-5.44149500	5.17465700	0.16381800
H	-5.49783500	4.35568000	-1.40659400

S_a-endo-6_c1

O	-0.52369500	2.14278800	1.67939100
O	0.52368900	2.14278500	-1.67939100
N	-3.45235600	1.43842200	-0.19296400
N	-1.63200300	0.33300000	0.75498300
N	1.63200100	0.33299900	-0.75498200
N	3.45235300	1.43842500	0.19296200
C	-5.70207100	-2.43739800	-0.34190500
H	-6.09315400	-2.19852700	0.65351900
H	-5.14370700	-3.37916700	-0.26574100
H	-6.55528100	-2.60857600	-1.00739000
C	-4.80601600	-1.31078700	-0.87805800
H	-5.41304700	-0.39866300	-0.93148700
C	-4.32712900	-1.63563300	-2.30095800
H	-5.18461500	-1.81864100	-2.95785500
H	-3.70205000	-2.53669700	-2.30891600
H	-3.74564700	-0.81944200	-2.74083700
C	-3.64387400	-1.06414400	0.10822800
H	-4.04204400	-0.96183500	1.12363700
H	-3.00293700	-1.95315100	0.11967700
C	-2.76968100	0.14429100	-0.21452200
H	-2.32645300	0.02276500	-1.20896400
C	-4.44692400	1.65965800	0.88310600
H	-4.11245200	1.25281800	1.85158300
H	-5.40155700	1.19406600	0.63060900
C	-4.52351900	3.18539100	0.96084700
H	-4.95115500	3.52829500	1.90604900
H	-5.14956700	3.56685700	0.14806000
C	-3.05562500	3.63351900	0.76721800
H	-2.97898400	4.53697400	0.15904800
H	-2.56388100	3.83806600	1.72230400
C	-2.38532200	2.42871600	0.07975500
H	-1.85770000	2.69758900	-0.84162100
C	-1.38550600	1.65867300	0.95252000
C	-0.92201800	-0.70460000	1.42547600
C	-1.08207000	-0.80651700	2.81286800
H	-1.72183400	-0.08922900	3.31466200
C	0.49280600	-2.65571800	1.48745700
H	1.10325100	-3.38648300	0.96707300
C	-0.10426000	-1.62615900	0.74168100
C	0.10425900	-1.62616100	-0.74167700
C	-0.49280500	-2.65572200	-1.48745000
H	-1.10325000	-3.38648600	-0.96706600
C	0.45370600	-1.81512800	-3.53686700
H	0.59162700	-1.87649500	-4.61122100
C	1.08207200	-0.80652300	-2.81286400
H	1.72183700	-0.08923600	-3.31465900
C	0.92201700	-0.70460300	-1.42547300
C	1.38550200	1.65867200	-0.95252000
C	2.38531800	2.42871800	-0.07975900
H	1.85769800	2.69759500	0.84161700
C	3.05562000	3.63351800	-0.76722700
H	2.97897000	4.53697900	-0.15906600
H	2.56388100	3.83805400	-1.72231800
C	4.52351700	3.18539400	-0.96084500
H	4.95115900	3.52830000	-1.90604400
H	5.14955700	3.56686200	-0.14805300
C	4.44692400	1.65966200	-0.88310600
H	4.11245400	1.25282300	-1.85158400
H	5.40155600	1.19407000	-0.63060700
C	2.76968000	0.14429400	0.21452200
H	2.32645400	0.02276700	1.20896500
C	3.64387500	-1.06414100	-0.10822900
H	4.04204400	-0.96183100	-1.12363700
H	3.00293900	-1.95314900	-0.11967700
C	4.80601900	-1.31078400	0.87805700
H	5.41304500	-0.39865600	0.93149200
C	4.32713200	-1.63563900	2.30095400
H	3.74564400	-0.81945500	2.74083500
H	5.18461800	-1.81864400	2.95785200

H	3.70206000	-2.53670900	2.30890700
C	5.70208100	-2.43738500	0.34189700
H	5.14372300	-3.37915600	0.26572500
H	6.55529000	-2.60856300	1.00738300
H	6.09316500	-2.19850300	-0.65352400
C	-0.45370200	-1.81512000	3.53687200
H	-0.59162100	-1.87648300	4.61122700
C	0.33106800	-2.75345400	2.86716600
H	0.81467100	-3.55709500	3.41239400
C	-0.33106400	-2.75346100	-2.86715900
H	-0.81466600	-3.55710400	-3.41238700

R_a-endo-6_c1

O	-0.37624100	2.13630400	-1.62597400
O	0.37622800	2.13629100	1.62597900
N	-3.57959900	1.43416300	-0.21104900
N	-1.65214400	0.33121600	-0.92041500
N	1.65214000	0.33121300	0.92041200
N	3.57959500	1.43417100	0.21106300
C	-5.34670200	-2.45360200	1.14559200
H	-4.95191000	-2.21532900	2.13958400
H	-4.88461300	-3.39348200	0.81746100
H	-6.42383500	-2.62828900	1.24317300
C	-5.06231700	-1.32451400	0.14390400
H	-5.54338200	-0.41282300	0.52064000
C	-5.68081600	-1.65310300	-1.22386500
H	-6.75210600	-1.85784900	-1.12025800
H	-5.21176000	-2.54429600	-1.66025600
H	-5.57309000	-0.83185300	-1.93932300
C	-3.54094500	-1.07236200	0.06618000
H	-3.12808900	-0.99200000	1.07599100
H	-3.06834700	-1.94793600	-0.39479700
C	-3.14458700	0.15258000	-0.74512700
H	-3.55934200	0.05624400	-1.75832200
C	-3.47164600	1.64210000	1.25120300
H	-2.55181400	1.20601400	1.66829300
H	-4.32910400	1.20111200	1.76523100
C	-3.42159900	3.16616700	1.36594500
H	-3.03394300	3.49546900	2.33316200
H	-4.42724300	3.58186100	1.24302200
C	-2.51407700	3.58319700	0.18765900
H	-2.80005800	4.54685300	-0.23845500
H	-1.47082700	3.64823700	0.50427400
C	-2.68270900	2.43338500	-0.82990400
H	-3.09637400	2.78170700	-1.78541100
C	-1.40569400	1.65795700	-1.16860400
C	-0.84089700	-0.68918300	-1.51275900
C	-0.89402600	-0.82708800	-2.90663000
H	-1.51291900	-0.14016400	-3.47447200
C	0.65519100	-2.57378700	-1.42963000
H	1.25708200	-3.26665800	-0.85148800
C	-0.04204400	-1.56117300	-0.74906800
C	0.04205100	-1.56118200	0.74904600
C	-0.65517900	-2.57380800	1.42959600
H	-1.25706400	-3.26667600	0.85144600
C	0.17937500	-1.82479400	3.56170600
H	0.23576200	-1.91377700	4.64151300
C	0.89402600	-0.82711400	2.90661600
H	1.51291400	-0.14019200	3.47446600
C	0.84089800	-0.68919600	1.51274600
C	1.40568400	1.65795100	1.16860900
C	2.68269700	2.43338600	0.82991900
H	3.09635600	2.78170500	1.78543000
C	2.51406500	3.58320300	-0.18763800
H	2.80004300	4.54685800	0.23848200
H	1.47081500	3.64824400	-0.50425300
C	3.42159000	3.16618200	-1.36592400
H	3.03393500	3.49548700	-2.33314000
H	4.42723200	3.58188100	-1.24299700
C	3.47164600	1.64211500	-1.25118900
H	2.55181900	1.20602400	-1.66828500
H	4.32910900	1.20113500	-1.76521600
C	3.14458500	0.15258400	0.74513200
H	3.55933300	0.05624500	1.75833000
C	3.54095200	-1.07235600	-0.06617600
H	3.12810200	-0.99199100	-1.07599000

H	3.06835300	-1.94793200	0.39479600
C	5.06232400	-1.32450400	-0.14389100
H	5.54339200	-0.41280800	-0.52061200
C	5.68081400	-1.65311100	1.22387800
H	5.57308000	-0.83187000	1.93934600
H	6.75210400	-1.85785300	1.12027800
H	5.21175600	-2.54431100	1.66025300
C	5.34671900	-2.45358000	-1.14559100
H	4.88462600	-3.39346400	-0.81747600
H	6.42385200	-2.62826700	-1.24316500
H	4.95193500	-2.21529400	-2.13958300
C	-0.17936900	-1.82475600	-3.56173200
H	-0.23575700	-1.91372800	-4.64154000
C	0.59747900	-2.70907900	-2.81413600
H	1.15504200	-3.50135500	-3.30275200
C	-0.59746600	-2.70911400	2.81410100
H	-1.15502300	-3.50140000	3.30270800

S_a-exo-6_c1

O	-0.20974700	1.87672700	1.44986600
O	0.12376200	1.57276800	-1.81957500
N	-3.56383500	1.33674800	0.46882100
N	-1.59764400	0.12588100	0.84965400
N	1.59843900	0.06071400	-0.87778700
N	3.47204700	1.44888700	-0.66159300
C	-4.45275600	-2.16834300	-2.52551300
H	-3.76174100	-2.99686800	-2.33547400
H	-4.09464800	-1.63025500	-3.41261600
H	-5.43283400	-2.59401400	-2.76892000
C	-4.54867300	-1.22444100	-1.31597100
H	-4.88925900	-1.82229800	-0.45690500
C	-5.59876400	-0.13533900	-1.58081700
H	-6.56626300	-0.58949200	-1.82487000
H	-5.29924200	0.48975100	-2.43199400
H	-5.73436300	0.52425100	-0.72106000
C	-3.14769700	-0.67638200	-0.96931000
H	-2.44405500	-1.51252500	-1.00230100
H	-2.82079900	0.04027600	-1.73331000
C	-3.02457400	-0.02541400	0.41321700
H	-3.53786200	-0.65367100	1.15110500
C	-4.28493400	1.66865400	1.71342300
H	-3.78909500	1.24362500	2.60417100
H	-5.30467100	1.27408400	1.67685100
C	-4.21942000	3.19500800	1.75509100
H	-4.40961400	3.59250600	2.75490000
H	-4.96519500	3.61772300	1.07412700
C	-2.79133600	3.50144700	1.24939000
H	-2.74957800	4.41829600	0.65817900
H	-2.08855100	3.61431500	2.07979800
C	-2.41164300	2.26301700	0.40887600
H	-2.16259300	2.52133100	-0.62557000
C	-1.24867100	1.44110400	0.96850900
C	-0.88774000	-0.90744300	1.54445400
C	-1.07328500	-0.96059200	2.93376200
H	-1.71905200	-0.22118700	3.39564200
C	0.53739600	-2.84031500	1.72255200
H	1.16983800	-3.58269700	1.24695100
C	-0.05558300	-1.85215100	0.91304800
C	0.18626000	-1.98619900	-0.56152700
C	-0.33665800	-3.14537000	-1.16664700
H	-0.92661300	-3.82199200	-0.55717600
C	0.62389300	-2.57311600	-3.29758900
H	0.79604900	-2.78344600	-4.34783700
C	1.16690500	-1.43266900	-2.71742900
H	1.77025900	-0.75538700	-3.31267500
C	0.96376800	-1.12842300	-1.36346600
C	1.17547400	1.30408800	-1.25057900
C	2.26826700	2.29186000	-0.84328300
H	1.95826700	2.74972400	0.10161500
C	2.62056900	3.34666200	-1.91597800
H	2.44063000	4.36260200	-1.55910300
H	1.99949900	3.19600600	-2.80375400
C	4.11023100	3.08542300	-2.22708000
H	4.37222800	3.30848700	-3.26414800
H	4.74962900	3.69115300	-1.57648700
C	4.27846100	1.60497200	-1.88834800

H	3.89532400	0.98195300	-2.71583900
H	5.31551400	1.31327300	-1.69938900
C	3.01226900	0.08295300	-0.38882200
H	3.58763900	-0.62506600	-0.99451200
C	3.13021800	-0.27754900	1.09465000
H	2.63654900	-1.23920900	1.26929700
H	2.57301400	0.47307800	1.66685300
C	4.57980600	-0.35537500	1.61189500
H	5.09493500	0.56237100	1.29989700
C	4.59027600	-0.40138600	3.14716200
H	4.09373400	0.47659100	3.57554200
H	5.61572900	-0.43105100	3.53281500
H	4.06951400	-1.29391300	3.51712300
C	5.34618700	-1.55688900	1.03579500
H	4.86067900	-2.50012500	1.31749000
H	6.37152200	-1.58261400	1.42164900
H	5.40995400	-1.52827200	-0.05703100
C	-0.46074200	-1.93322300	3.71515400
H	-0.62108900	-1.94930500	4.78796300
C	0.34987600	-2.88660500	3.09977100
H	0.83331700	-3.66172700	3.68512000
C	-0.13250200	-3.44066400	-2.51032800
H	-0.56112600	-4.34166000	-2.93649600

R_a-exo-6_c1

O	1.04656600	2.20901300	1.80806100
O	-0.98608600	2.42073200	-1.49976000
N	3.50144900	1.14078700	-0.56152600
N	1.79186200	0.28084600	0.76507200
N	-1.79843000	0.41200500	-0.68457400
N	-3.60523200	1.17303600	0.58447300
C	4.94076600	-2.52139600	-0.81564800
H	4.21953600	-2.24297300	-1.59077300
H	4.52181900	-3.36802100	-0.25671000
H	5.84588600	-2.87314800	-1.32323900
C	5.26919900	-1.35340000	0.12760400
H	5.66654400	-0.52628900	-0.47510800
C	6.35278400	-1.76672800	1.13459200
H	7.25446300	-2.11697300	0.61965100
H	5.99923300	-2.58210600	1.77886900
H	6.63730400	-0.92889200	1.78111400
C	4.02753500	-0.82946100	0.87319100
H	3.53543400	-1.66677200	1.38496300
H	4.34202700	-0.12305600	1.65224900
C	3.00090400	-0.11896000	-0.01974400
H	2.69102400	-0.77233200	-0.83761400
C	3.12383600	1.43004800	-1.96067900
H	2.10694900	1.07396100	-2.19214900
H	3.82036400	0.94164100	-2.64855500
C	3.17574100	2.95601300	-2.02747500
H	2.62892200	3.35470000	-2.88544500
H	4.21547400	3.29354200	-2.09807000
C	2.55830500	3.37723800	-0.67987900
H	2.91406200	4.34896300	-0.33214000
H	1.46851800	3.42516600	-0.75948500
C	2.96795400	2.23460700	0.28012000
H	3.71284900	2.57403300	1.01046700
C	1.81195400	1.61323600	1.05702900
C	0.94332000	-0.65722500	1.42815200
C	1.04004500	-0.78563800	2.81912600
H	1.73745900	-0.15285000	3.35575700
C	-0.70523100	-2.41840700	1.41412900
H	-1.38266900	-3.06193700	0.86204500
C	0.04321400	-1.46493800	0.70787500
C	-0.10200600	-1.38792000	-0.78058900
C	0.61665900	-2.29660400	-1.57295800
H	1.25644900	-3.02238600	-1.08118500
C	-0.28287600	-1.33888000	-3.59286600
H	-0.35511700	-1.30800600	-4.67492800
C	-1.02921100	-0.45182400	-2.82320300
H	-1.69060800	0.26169300	-3.30100800
C	-0.95660600	-0.47445400	-1.42457900
C	-1.80316400	1.76374100	-0.86284000
C	-3.03284200	2.32109400	-0.14598400
H	-3.71059300	2.70149000	-0.92087500
C	-2.72612100	3.40102800	0.90398700

H	-3.60447700	4.04144000	1.03492500
H	-1.89091300	4.03263800	0.59185400
C	-2.44748300	2.58477200	2.17580700
H	-1.41144700	2.23285100	2.17597700
H	-2.61354600	3.15929600	3.09113800
C	-3.42312700	1.40439300	2.04053200
H	-3.06556400	0.49497700	2.53168500
H	-4.39463400	1.65511900	2.48453000
C	-3.03638800	-0.04730600	0.02181300
H	-2.74943800	-0.72709500	0.82629400
C	-3.99553800	-0.75106700	-0.95010000
H	-3.45994300	-1.56376400	-1.45750300
H	-4.28015900	-0.02662200	-1.72411300
C	-5.26658100	-1.32027000	-0.29275300
H	-5.71712400	-0.51790600	0.30598000
C	-6.27966800	-1.72911400	-1.37243500
H	-6.54304500	-0.88080100	-2.01427800
H	-7.20273200	-2.10987900	-0.92088800
H	-5.87177400	-2.52073100	-2.01423300
C	-4.96892200	-2.50556300	0.63905100
H	-4.49397600	-3.32656000	0.08673000
H	-5.89516700	-2.89230700	1.07854700
H	-4.30551400	-2.23108800	1.46553900
C	0.26677300	-1.71744600	3.50562000
H	0.35640700	-1.80455300	4.58329800
C	-0.60328200	-2.54688700	2.79778200
H	-1.19974400	-3.28960700	3.31709800
C	0.53482200	-2.27769800	-2.96338900
H	1.10748200	-2.98942600	-3.54866500
